# Soft tactile chip with in-situ sensing for haptic rendering and reverse feedback enhanced gross to fine teleoperation

**DOI:** 10.1038/s41467-026-73000-8

**Published:** 2026-05-11

**Authors:** Minglu Zhu, Hao Ling, Rui Wang, Zhanpeng Du, Qi Sun, Kuihan Chen, Ke Chen, Lining Sun, Cheng Fan, Chengkuo Lee, Xuan Li, Tao Chen

**Affiliations:** 1 Jiangsu Provincial Key Laboratory of Embodied Intelligence Robot Technology, School of Mechanical and Electric Engineering, Soochow University, Suzhou, China; 2 School of Future Science and Engineering, Soochow University, Suzhou, China; 3 Department of Electrical & Computer Engineering, National University of Singapore, 4 Engineering Drive 3, Singapore, Singapore

**Keywords:** Electrical and electronic engineering, Mechanical engineering

## Abstract

In-situ sensing of feedback actuator offers spatiotemporal consistency of tele-haptic interactions and self-monitoring haptic feedback. Leveraging haptic actuator for fine teleoperation is also worth for investigation. Here, we report a soft tactile chip made by in-situ fabrication of sensor in actuator structure, which is featured by both haptic and thermal sensing and feedback. This chip consists of silicone based pneumatic actuator arrays with two elastomeric membranes which contain liquid metal micro channels, and pectin-based temperature sensor. In-situ sensing of tactile chip enables self-adaptive pneumatic haptic feedback via quantitative data compared to subjective test. In addition to direct haptic feedback during gross teleoperation stage, a concept of reverse haptic feedback allows finger micro-motions from leader side to be directly projected into follower side for manipulating target object during fine teleoperation stage. In general, the proposed device can be applied as modular component to realize mutual tactile perception and micro manipulations.

## Abstract (web summary)

Soft tactile chips embed in-situ sensing in pneumatic actuators, improving teleoperation via adaptive haptic feedback. Liquid-metal pressure and pectin temperature sensors enable precise manipulation and better human-robot interaction.

## Introduction

Robotics and artificial intelligence are experiencing tremendous advancements now. Human-robot collaborative operation eventually illustrates great capabilities and potential in conducting various complex tasks^1^. Even for humanoid robots with high autonomous decision-making ability, their training stages still heavily rely on different human machine interfaces, such as teleoperation systems. The dexterous operations usually consists of gross and fine manipulations to complete the entire tasks^2^. By considering operation complexity and features of target object, tactile information become important for ensuring efficiency and safety during the operations^3^, including medical surgery, precise assembly, etc. Hence, detection of different parameters, e.g., temperature, pressure, texture, hardness, shape, dynamic motions, and reconstruction of them with high spatial consistency are crucial when establishing a tactile communication pathway during human machine interaction^4–6^. Secondly, unlike the advanced dexterous robotic hands with high cost, many of the commercial robotic grippers still lack enough motional degree of freedom (DoF) to implement fine manipulation at the final stage. Exploration of tactile sensing and feedback technique in a reverse manner, e.g., delivery tactile information of user to robot side for actuation, can be a promising way to enable gross to fine teleoperation on most of the current robotic systems, without changing their main infrastructures.

Haptic feedback technologies offer intuitive and realistic stimulus to the user without any learning cost^7,8^. Human skin perceive various stimulations via mechanoreceptors with slow adaption (SA) and fast adaption (FA) mechanisms, and thus, different types of haptic feedback working principles have been reported, including electromagnetic^9,10^, dielectric elastomer^11^, pneumatic^12,13^, hydraulic^14^, electric discharge^15^, electroosmotic^16^, piezoelectric^17^, etc. For instance, Shultz et al. presented a shape-changing display using embedded electroosmotic pump array with a thickness under 5 mm and relative low actuation voltage^18^. A soft wearable pneumatic haptic device, Haptiknit, was reported with distributed stiffness knitting technique for delivery different messages^19^. In addition, reconstruction of multi-modal and multi-dimensional tactile information remains challenging. A wireless electromechanical haptic interface integrating an array of bistable transducers with kirigami design can provide indentation, shear and vibrotactile stimuli^20^. Interestingly, to overcome the issue of wearable comfortability, a fully integrated breathable haptic textile was fabricated by electrospin, with 128 pixels of actuators over the palm^21^. Recently, an electromagnetic based full freedom-of-motion haptic device which provides pression, shear, and vibration feedbacks is further paving the way for reconstruction of multi-dimensional perception^22^. On the other hand, thermal feedback as another key function during interactive event, is also studied frequently in terms of regenerating temperature profiles^23–25^. However, many of these haptic feedback devices do not integrate sensors within their actuation structures, restricting their application of bi-directional tactile communication.

Tactile sensor, is another key role in close loop tactile communication^26,27^. Numbers of tactile sensing mechanisms have been well studied, such as piezoresistive, capacitive, piezoelectric, triboelectric, electromagnetic, optical, etc. Large and dense array of sensors is one of the popular research areas^28^. To simplify the electric connection issue, a neuro-inspired asynchronously coded electronic skin with one readout port can support scalable sensors by specific coding^29^. For better mimicking three dimensional sensing of human mechanoreceptor, Liu et al. developed a three-dimensionally architected electronic skin which is capable of decoupled sensing of normal and shear forces and strains^30^. On the other hand, temperature information is another major parameter expected during specific operations^31^. Inspired by biological membranes, a high sensitive thin film made by pectin was reported with capability of remote sensing^32^. Robotic manipulators with the abovementioned tactile sensor are showing great capabilities in perceiving multi-modal stimul. Moreover, the close loop sensing and feedback system with accurate projection of perception requires high spatial consistency between sensors and actuators, which is also essential for sending the fine manipulation command from user to robot.

As another critical issue, although haptic feedback highly depends on subjective perception, the repeated subjective tests required in the design of massive haptic feedback patterns are time and personnel consuming^12^. Thus, to develop an approach with more objective evaluation standards, establishment of quantitative databases is important to conduct the pre-adjustment of feedback parameters^33^. Most of current quantitative evaluations of haptic feedback program rely on external sensors or special instruments, which may lead to mismatch of spatiotemporal information between practical applications and standard test setup, due to unexpected mechanical distortion or displacement. In-situ sensing data about those actuators can be considered as a promising solution. In addition, the self-adaptive perceptual rendering of several main physical properties, such as hardness, relies on the real-time tuning of the actuation parameters in response to user’s activities, and thus, requires in-situ sensing of the corresponding actuators as well.

**Fig. 1:**
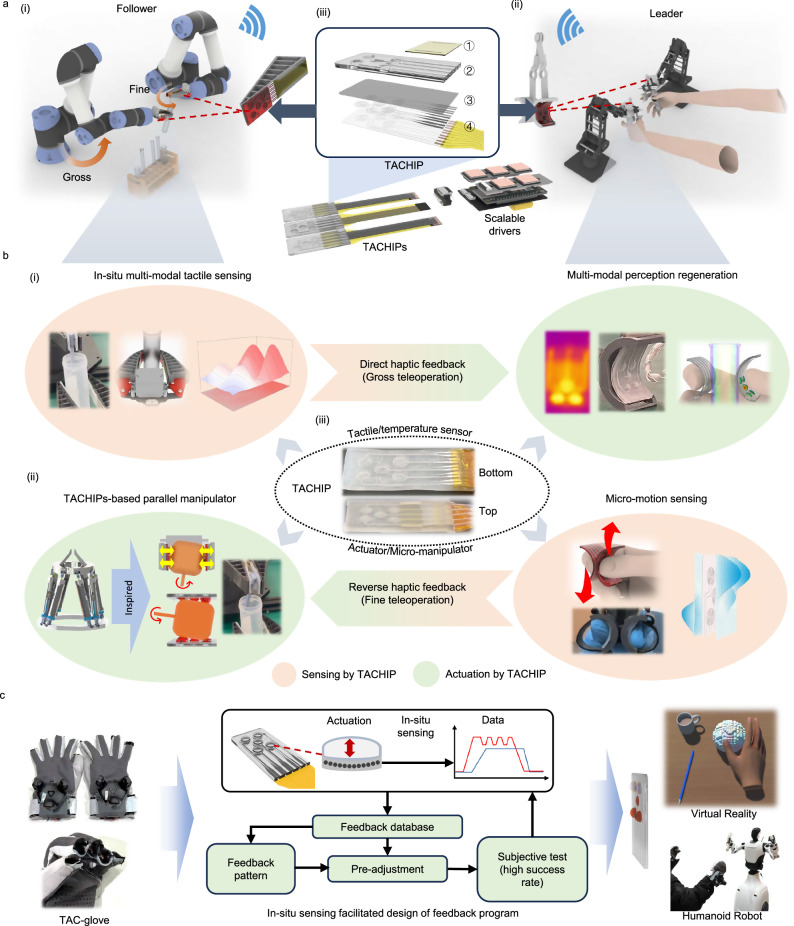
Tactile chip (TACHIP) enhanced gross to fine teleoperation. **a** Schematics of wireless modular TACHIP system on (i) leader and (ii) follower sides with multi-modal haptic rendering and reverse feedback for gross to fine teleoperation, with (iii) the exploded view of (1) pectin temperature sensing layer, (2) pneumatic actuators layer, (3) liquid metal micro-channels layer, (4) flexible print circuit. **b** Schematics of TACHIP enabled (i) mutual perception with tactile sensing and feedback, and (ii) reverse feedback-based micro manipulation via micro motion sensing and pneumatic parallel manipulator, (iii) real photos of TACHIP and modular processing system. **c** Left: photos of TACHIPs embedded data glove. Middle: TACHIP in-situ sensing facilitated design of haptic feedback pattern for human machine interactions. Right: applications of virtual and real world interactions. Photo credit: Minglu Zhu, Soochow University.

Our previous research utilizes the simple stacking strategy of tactile sensor and rigid haptic feedback actuators to develop sensing and feedback integrated devices^12^. However, without the design of in-situ sensing unit in the actuation structure, it still suffers bulky size with low flexibility, lack of temperature information, as well as low spatiotemporal consistency of tele-haptic communication. To overcome these issues, in-situ sensing and feedback mechanisms in a single structure shows good potential^34^. Here, we report a soft tactile chip (TACHIP) made by in-situ fabrication of thin film sensors in the membranes of pneumatic actuator (Fig. 1a). This chip is fabricated by flexible pneumatic actuators, featured by bottom polydimethylsiloxane (PDMS) actuation membrane (thickness: 0.3 mm) with encapsulated liquid metal micro channels, and top layer with pectin-based temperature sensing film (thickness: 0.1 mm). TACHIP with in-situ sensor possesses both haptic and thermal sensing and feedback with good spatial consistency, and is originally equipped with flexible print circuit (FPC) for quick plug-in. In a single TACHIP, the operation frequency of pneumatic feedback ranges from 0 to 1000 Hz with a maximum output force of 1.67 N. The in-situ force and temperature sensor have sensitivities of 1.59 N^−1^ and 0.325 °C^−1^. In-situ sensing function also allows the monitoring of pneumatic actuation for self-adaptive haptic rendering. As shown in Fig. 1b(i), TACHIPs in teleoperation system can regenerate tactile information from follower to leader during gross teleoperation stage. Moreover, inspired by Stewart parallel manipulator, the design of five pixels also builds a micro manipulator when grabbing the objects (Fig. 1b(ii)), enabling multiple DoFs motions to the normal gripper. Together with a concept of reverse haptic feedback, finger micro-motions from TACHIP at leader side can then be directly projected into TACHIP at follower side for fine tuning of the target object. With the portable and scalable system developed for TACHIPs, the proposed devices can be quickly integrated into those commercial human machine interfaces, such as robotic teleoperation systems, and data gloves shown in Fig. 1c. In addition, instead of traditional subjective test, a methodology of evaluating haptic feedback performance with quantitative and objective method is proposed, TACHIPs with in-situ sensing allows the establishment of haptic feedback sensing database to facilitate the design of feedback pattern through data assisted pre-adjustment. As a result, the identification accuracy of the first-round subjective test can reach about 95%, compared to 85% in our previous woek^12^. In general, the proposed device significantly improves the success rate in typical teleoperations, and can be applied as scalable add-on modular chips compatible with the current commercial human machine interfaces (HMIs) to realize mutual tactile perception and intuitive micro manipulations in robotic teleoperation, VR training and communication, etc.

## Results

### Design and working principle of TACHIP

**Fig. 2:**
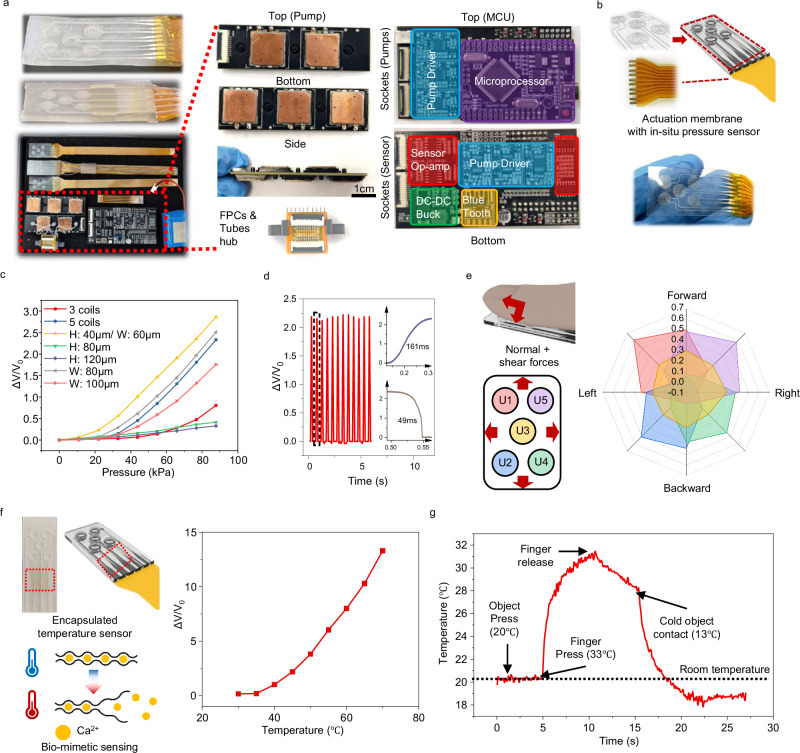
Characterization of liquid metal-based in-situ pressure sensor and pectin-based temperature sensor. **a** Photos of TACHIP system, with scalable piezo pumps module, wireless microprocessor with drivers, and customized hub for quick connection of FPCs and air tubes. **b** Schematics and real photos of pressure sensor for tactile sensing. **c** Characterization data of liquid metal-based micro-channels with different design parameters, H and W are the height and the width of the micro channel, respectively. **d** Response time of liquid metal-based pressure sensor. **e** Detection of normal and shear forces applied by finger at eight directions via sensing array, the radar plot indicate the pressure distribution when pushing TACHIP toward the corresponding direction. **f** Working principle of temperature sensor based on pectin thin film and characterization data of temperature sensing performance. **g** Temperature sensing data for monitoring the contacts of objects with three different temperatures: 20 °C (room temperature), 33 °C, and 13 °C. Photo credit: Minglu Zhu, Soochow University.

As shown in Fig. 2a, a portable and scalable TACHIP system is developed with compact size and fast deployment desig. TACHIP is fabricated by bonded silicone and PDMS double layer, and forms a soft chip with the size of 35 mm × 20 mm × 3 mm (also can be seen in Supplementary Fig. 1 and Supplementary Note 1). There are five pixels of pneumatic actuators in silicone layer, with a layout of four actuators located at the corners and one actuator located at the center. Each pneumatic actuator has a diameter of 4.5 mm and a height of 0.8 mm. To form the encapsulated pneumatic chambers, PDMS thin film (0.3 mm) was bonded to silicone layer. Five units of coil shaped liquid metal micro channels were fabricated inside the PDMS thin film, and are located exactly at the bottom of five pneumatic actuators. Consequently, PDMS film with liquid metal units forms the bottom membranes of pneumatic actuators, with in-situ pressure sensing and thermal feedback capabilities. Additionally, a transparent thin film temperature sensor made by pectin was also encapsulated in TACHIP. To ensure the compatibility of TACHIP to the commercial print circuit board (PCB), FPC connector was encapsulated within PDMS thin film (0.3 mm) for fast plug-in to the microprocessor, and hence, allow TACHIPs to be quickly integrated with most of the current robotic or VR platforms. Combination of multiple TACHIPs can achieve high scalability for distributive deployment. Detailed fabrication process can be seen in method part and supplementary information. Meanwhile, the back-end processing module contains micro-computer unit (MCU), piezo-pump, and battery, which can be quickly assembled via FPCs (Fig. 1b). The small size of piezo-pump offers better portability and higher operation frequency compared to other air pumps, while maintaining the advantages of pneumatic feedback.

In Fig. 1a and b, TACHIPs with back-end processing modules act as universal tactile interfaces to provide multi-modal sensing and feedback functions, as well as micro-manipulator for both leader and follower sides, and hence, establishing a close-loop tactile communication system without other components. Specifically, two TACHIPs are attached on fin-ray soft grippers at follower side (single arm), and two TACHIPs are placed in handle lever at leader side (single arm).

### Characterization of In-situ pressure sensor of actuator for tactile detection

Sensing and feedback share equal importance in developing a complete tactile communication system. To enable haptic feedback in teleoperation, sensing nodes with high spatial consistency are required. As a result, liquid metal possesses advantages such as stretchability, which are desirable for making in-situ sensor in membrane of pneumatic actuator. As shown in Fig. 2b, schematics of liquid metal based piezoresistive sensor is given. The photos show the good deformability and conformability with FPC. The design parameters of the microfluidic channel of liquid metal affect the performance of the as-fabricated sensor, including width, height, turns of coil structure, etc. According to characterization data in Fig. 2c (also can be seen in Supplementary Fig. 2 and Supplementary Note 2), a design of channel with five turns, 60 μm width, and 40 μm height was chosen. Temperature effect to piezoresistive sensors is also investigated, as shown in Supplementary Fig. 3. The cyclic test and the repeatability test shown in Supplementary Fig. 4 and Supplementary Fig. 5 prove the stability of the as-fabricated pressure sensor. As the main purpose of pressure sensors is to offer general pressure distribution for haptic feedback, the performance of liquid metal-based sensors still meets the requirement (also can be seen in Supplementary Fig. 6). In addition, pectin temperature sensor in silicone layer can be utilized as reference sensor for compensation. Figure 2d shows the response of the proposed sensor to varied frequencies, the inset graph of zoom-in data indicates a response time of about 49 ms. During dexterous operations, three-dimensional (3D) force sensing is essential for detecting finger micro-motion and fine tuning at follower side. The layout of five pressure sensors offers 3D force sensing or pressure mapping by analyzing signals simultaneously (also can be seen in Supplementary Fig. 7, Supplementary Fig. 8, and Supplementary Note 3). Figure 2e presents a radar plot which shows the pressure variation for applying shear force via finger at eight directions.

### Characterization of pectin-based thin film in-situ temperature sensor

Inspired by the sensing mechanism of pit membranes, biomimetic temperature sensing film with high sensitivity and responsivity was reported^32^. Pectin has the structural complex of acid-rich polysaccharides which can bind ions, such as Ca^2+^. As the temperature increasing, the ionic conduction will increase due to the exponential reduction of the cross-linking of pectin molecules. In the meantime, this kind of temperature sensing mechanism leads to a much lower sensitivity to mechanical stimuli, such as bending and pressing. Therefore, it is a favorable material for fabricating in-situ temperature sensors in the structure of TACHIP. As shown in Fig. 2f, as-fabricated temperature sensor has relative good responsivity within the working range. Figure 2g gives a continuous monitoring data for recording signal response against three contact events, including pressing by object with room temperature (20 °C), pressing of finger (33 °C), and contact of cold object (13°C). The presented data indicates that pectin temperature sensors have very low sensitivity to mechanical stimulus, and in contrast, has high sensitivity to temperature variations. Thus, this thin film sensor is favorable to be integrated into silicone layer of pneumatic actuator. It also acts as reference sensor for compensating the temperature effect of liquid metal-based pressure sensor.

### Characterization of pneumatic haptic feedback and in-situ monitoring of actuation

**Fig. 3:**
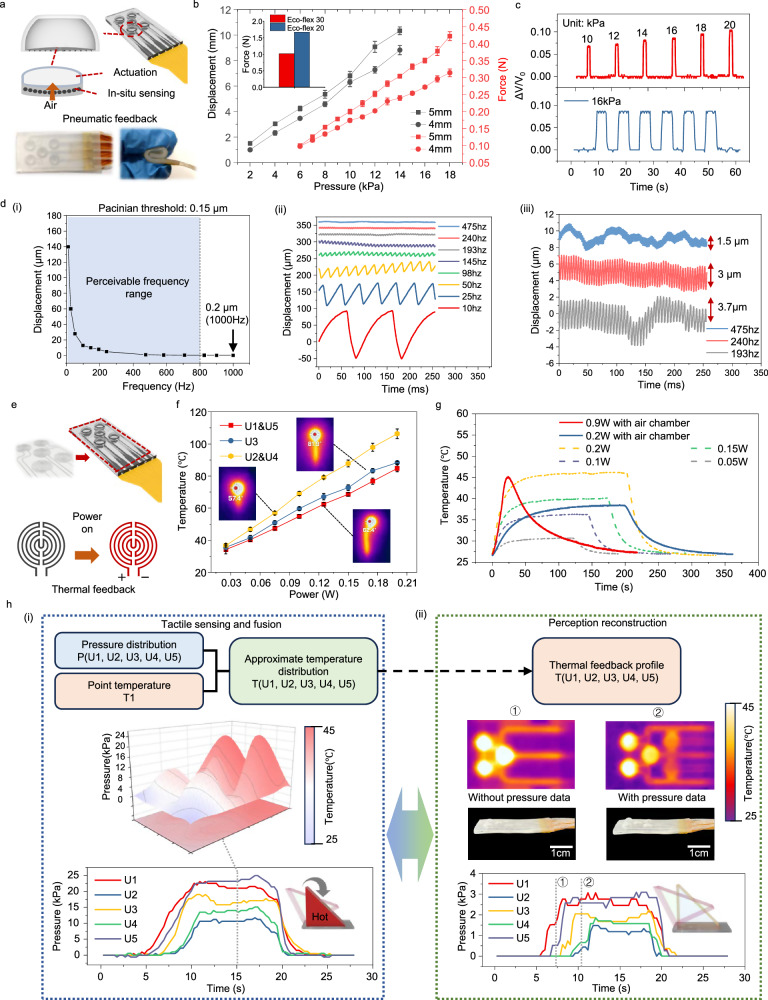
Characterization of pneumatic and thermal feedback. **a** Working principle of pneumatic feedback with in-situ pressure sensing, and the photos of soft TACHIP. **b** Characterization data of pneumatic output forces with different design parameters, the inset graph shows the maximum output force of pneumatic actuator made by different elastomeric materials. Error bars indicate the standard deviations across measurements. **c** Sensitivity and stability of in-situ monitoring of pneumatic actuation via liquid metal pressure sensor of TACHIP. **d** Characterization data of (i) operation frequency range of TACHIP with displacements above Pacinian threshold, the shaded area indicates the typical perceivable frequency range of human mechanoreceptors, (ii) actual operation frequency and displacements measured by vibrometer for the driven frequencies of 10 Hz, 25 Hz, 50 Hz, 100 Hz, 150 Hz, 200 Hz, 250 Hz, 500 Hz, respectively, (iii) magnified high frequency data of 200 Hz, 250 Hz, and 500 Hz. **e** Working principle of thermal feedback based on liquid metal pressure sensor. **f** Characterization data of thermal feedback performances under varied power supply for the corresponding liquid metal units, the inset thermal images show the status of three units with different connecting lengths. Error bars indicate the standard deviations across measurements. **g** Evaluation of response time of thermal feedback under different conditions, including changing power supply and gap by air chamber. **h** Fusion of haptic and thermal sensing and feedback, (i) top: schematics of temperature approximation approach with fusion of pressure distribution and single temperature data, bottom: pressure sensing data during sequential contact, with color map for visualization of pressure and temperature profiles at full contact stage, (ii) top: schematics of thermal feedback profile enabled by approximated temperature distribution, bottom: pressure sensing data for monitoring pneumatic haptic feedback, with photos and infrared images of thermal and haptic feedback at two corresponding stages: initial contact and full contact. Photo credit: Minglu Zhu, Soochow University.

A single TACHIP has five pneumatic actuators, four actuators at four corners and one at center. Each air chamber of pneumatic actuator is connected to a piezo-pump. This design of five actuators ensures minimal number of feedback units while still maintaining the capability of reconstructing the perception of various shapes, dynamic motions, etc. The optimized number of actuators also miniaturized the back-end processing module, and offers larger haptic feedback force from each unit, which is also important for replicating the different levels of hardness of target object. In Fig. 3a, a basic working principle of pneumatic actuation with in-situ sensing through liquid metal-based pressure sensor is given. Film with embedded liquid metal micro channels act as one membrane of silicone pneumatic actuator, and hence, the inflation of actuator will apply air pressure to liquid metal film and lead to deformation. Figure 3b shows the characterization data of pneumatic actuators with different diameters under varied input air pressure (also can be seen in Supplementary Fig. 10 and Supplementary Note 4). Larger actuator shows higher displacement and feedback force at the same input pressure. The 5 mm actuator reaches about 10 mm displacement at 12 kPa gauge pressure, and 0.4 N feedback force at 18 kPa gauge pressure, which shows advantages compared to other haptic feedback mechanisms with high frequency. To further explore its maximum output force, the as-fabricated pneumatic actuators can achieve 1 N (Eco-flex 00-30) and 1.67 N (Eco-flex 00-20) at 60 kPa input pressure. The hysteresis test (can be seen in Supplementary Fig. 11) indicates a relative good dynamic response of elastomeric actuator.

As an in-situ pressure sensor, the liquid metal unit can then monitor the real-time input air pressure during pneumatic haptic feedback. To prove the reliability of using in-situ pressure sensor for monitoring pneumatic actuation, the experimental data for detecting the increasing air pressure and the cyclic inflation are shown in Fig. 3c (also can be seen in Supplementary Fig. 9). The in-situ monitoring of stepwise increasing of input air pressure was conducted by controlling the input power of the piezo pump, and the real input pressures were also monitored via commercial manometer. The in-situ monitoring of cyclic inflation was performed by cyclically activating the piezo pump to provide a fixed pressure (16 kPa) with a fixed frequency (8 sec/cycle). The results indicate good sensitivity and repeatability of the as-fabricated pressure sensors which are comparable to force gauge. In addition, the stability tests and the cyclic test with no obvious degradation of mechanical and electrical performances shown in Supplementary Fig. 12 and Supplementary Fig. 13 prove the durability of both sensing and feedback functions. Compared to electromagnetic based vibrators, pneumatic actuator with the large deformation possesses relative better performance in generating shape information, while it still can provide vibrational stimulus with piezo pump. Meanwhile, the photos of rollable real TACHIP (Fig. 3a) also indicate its good flexibility, which can hold for repeated folding or rolling within the reasonable force range, the excessive external forces (above 30 N) applied to the TACHIP may also cause the leakage of the liquid metal and affect the sensing performance.

In terms of vibrational stimulus, the pneumatic actuator offers vibrational feedback with higher frequency depending on response time of piezo-pump. Figure 3d(i) shows the characterization data of operation frequency range of TACHIP with displacements above Pacinian threshold for mechanoreceptor of human skin. It can be seen that the TACHIP can cover the entire range of human perceivable frequency. Figure 3d(ii) depicts the actual vibrational data, including the actual frequencies and the displacements recorded by laser doppler vibrometer for pneumatic actuator operated under the driven frequency of 10 Hz, 25 Hz, 50 Hz, 100 Hz, 150 Hz, 200 Hz, 250 Hz, 500 Hz, respectively. The enlarged data in Fig. 3d(iii) indicates that the vibrational pneumatic haptic feedback at 475 Hz (driven frequency of 500 Hz) can generate 1.5 µm displacement which is still perceivable, according to the Pacinian threshold of humans. The corresponding demonstrations also can be seen in Supplementary Movie 1.

### Characterization of sensing elements enabled thermal feedback

Feedback of thermal information is another key parameter that needs to be perceived when operating hot objects or in high temperature environment. Leveraging the multiple properties of liquid metal, an in-situ heater is simply obtained by switching the connection of liquid metal from analog sensing input to digital output for controlling power supply (Fig. 3e). As shown in Fig. 3f, depending on the lengths of the liquid metal channels, the power consumption of specific liquid metal unit for a given temperature varies. Meanwhile, the average power consumption with pure liquid metal membrane ranges from 0.05 W to 0.10 W for heating from 35 °C–50 °C. However, user’s skin actually contacts with the membrane of pneumatic actuation, which is the opposite side of the liquid metal membrane. The gap of air chamber affects the thermal expansion for a given supply power (Fig. 3g). To reduce the response time of thermal feedback, a higher power is required at initial stage, as shown in red data curve of Fig. 3g. Hence, to achieve better response time while ensuring less deviation from target temperature, proportional-integral-derivative (PID) control like gradient power supply strategy for heating is adopted.

### Fusion of pneumatic and thermal haptic feedback

Mechanical and temperature information are two key tactile information which affect efficiency and safety during human machine interactions. Enhanced perception regeneration provided by fusion of mechanical and temperature feedback can offer comprehensive understanding of real-time on-site conditions (e.g., heating status of grabbed object, stiffness variation under changing temperature, etc.) during teleoperation and assist the corresponding adjustment. By properly switching the function of in-situ liquid metal micro channels between sensor and heater, multi-pixel haptic and thermal sensing and feedback can be done simultaneously without adding extra components (Fig. 3h). Currently, most pressure and temperature sensing arrays usually require multiple readouts for both signals. In practical application, the high resolution of temperature detection may not be necessary, due to thermal radiation and the limitation of human skin’s perception to temperature information. With the concept of minimalistic design and the aid of five pressure sensors on TACHIP, a single temperature sensing layer may also realize the mapping of temperature distribution via a reasonable approximation done by fusing of temperature data and pressure distribution data. Figure 3h(i) shows the experimental data recorded by sequential contact (from initial to full contact) of a hot object. Temperature and pressure distribution data extracted from full contact stage is visualized by 3D maps. Owing to the sensing fusion method, perception reconstruction via haptic and thermal feedback of TACHIP can be achieved with more details, as illustrated in Fig. 3h(ii). As a comparison, the thermal feedback of the second contact stage with pressure information (right) is showing better temperature profile compared to that of initial sage (left) when the temperature approximation method is disabled. In general, fusion of thermal and haptic feedback with varied detail information across the area can give enhanced perception recreation during precise teleoperation (also can be seen in Supplementary Fig. 17, Supplementary Fig. 18, Supplementary Fig. 19, and Supplementary Note 7).

### Slow/Fast adapting and in-situ sensing enabled multi-modal haptic rendering

**Fig. 4:**
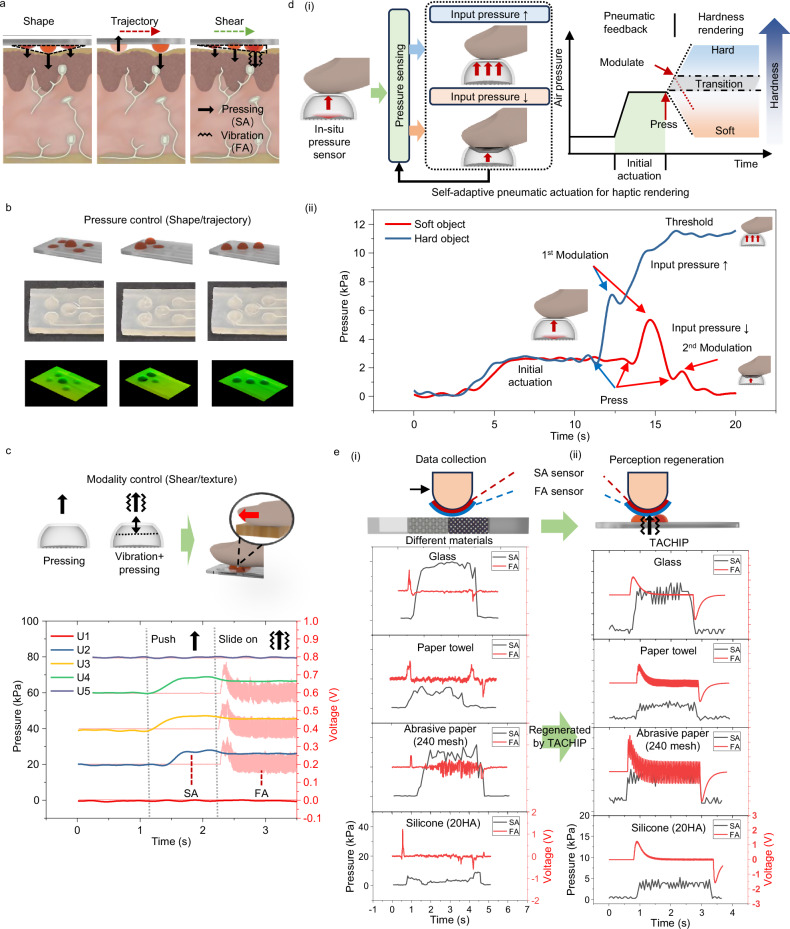
Slow-adaption (SA), Fast-adaption (FA), and in-situ sensing enabled multi-modal and self-adaptive haptic rendering. **a** Different modalities of pneumatic feedback for reconstructing the corresponding interactive perception. **b** Typical patterns for regeneration of shape and geometry via pneumatic actuator array and pressure control, the corresponding photos and laser profiles from real activated TACHIPs are shown below. **c** Modality control for generating single or fused feedback on each actuator to mimic process of sliding motion, the right *y*-axis shows the pressure data, the left *y*-axis shows the vibrational signal recorded by piezoelectric sensor. **d** (i) Schematics of in-situ sensing enabled self-adaptive pneumatic actuation for haptic rendering of hardness, including theoretical trend of pressure variation of the actuator for mimicking hard and soft material during finger pressing. (ii) Experimental data of pressure sensing of the actuator for mimicking hard and soft material during finger pressing. **e** Perception regeneration of different materials, including glass, paper towel, abrasive paper (240 mesh), and silicone (20 HA), (i) data collection of SA and FA sensing signals through sliding based scanning of commercial piezoresistive (SA) and piezoelectric (FA) sensor, (ii) TACHIP regenerated mechanical stimuli monitored by SA and FA sensor. Photo credit: Minglu Zhu, Soochow University.

Modalities and dynamic adaptability of haptic feedback are critical in replicating the perceptions as realistic as possible. Figure 4a shows typical modalities of pneumatic feedback for recreating the perceptions of various interactive events. A system latency test shows a response time of about 200 ms between sensing and feedback (Supplementary Fig. 13). With the aid of tunable input air pressure and the layout of five actuators, several typical patterns of object can be regenerated to understand the contact status (also can be seen in Supplementary Fig. 15 and Supplementary Note 5). Moreover, the displacement variations with actuator array can further provide shape or geometry information. High operation frequency of piezo pumps also introduces vibrational actuation to pneumatic chambers, which are then featured with both vibration and pressing based haptic feedback, and these two modes can be activated simultaneously on the same unit. Figure 4b shows the schematics and the actual laser profiles of the examples of pneumatically regenerated shapes from TACHIP. Figure 4c illustrates the modality control of pneumatic array done by modulating cycling frequency and input power via program. Thus, reconstruction of SA and FA perception is achieved without additional actuators. As can be seen in the experimental data of mimicking mechanical stimuli during sliding process, three units (U2, U3, and U4) were activated to provide static pressure at initial stage, and followed by adding vibrational feedback for the onset of sliding. Noticeably, the decreasing feedback pressure of pneumatic actuators was observed once the vibration-pressing mode initiated, due to the limited cycling time for complete inflation.

Except establishing sensing and feedback during human machine interactions, another key concept of introducing in-situ sensor in actuator is to build a close-loop feedback system for self-adaptive pneumatic actuation with better haptic rendering. As an example, the process of pneumatic feedback-based hardness rendering with the aid of pressure sensing is shown in Fig. 4d. After pneumatic actuator was inflated, finger touch would cause deformation of the actuator due to the nature of silicone-based membrane. Hence, the consequent reaction of the actuator is crucial for regenerating the perception of hard or soft objects. Specifically, the feeling of hard object will be created if higher resistive force generated by increasing input pressure is felt after finger touches object. In contrast, the soft feeling will be sensed if the resistive force is reduced by lowering input pressure. The way of achieving this type of self-adaptive pneumatic feedback relies on the in-situ pressure sensor. As shown in the sample procedure of Fig. 4d(i), the pressure sensing signal reaches a target level during the initial actuation on the basis of feedback events. As the user touches the actuator to further explore object properties, compression of pneumatic chamber further increases the internal pressure. Once the pressure reaches the threshold for modulating input air pressure, inflation or deflation program will proceed based on the predefined or sensed data regarding the object hardness. Thus, the pressure may either be increased or decreased accordingly. The experimental data is also provided in Fig. 4d(ii) to verify the proposed self-adaptive pneumatic haptic feedback technique. The presented data includes the monitoring signals of touching soft and hard objects, respectively, with the marked features as described above. Further modulations can also be performed based on requirements. A threshold of maximum pressure is set for each actuator to stop the air supply in case of potential damage.

As shown in Fig. 4e, to further explore the capability of TACHIP in replicating the physical properties of different materials through SA and FA stimuli, the tactile information was collected by using commercial piezoresistive (SA) and piezoelectric (FA) sensors to scan across the materials and build the reference database (Fig. 4e(i)). Frequency and amplitude of a specific material was analyzed to guide the programming of the code of driving the pneumatic pump. Moreover, to verify the regenerated stimuli from TACHIP, the same SA and FA sensors were placed on the top of TACHIP to collect the replicated tactile information (Fig. 4e(ii)). According to the comparisons between the collected data and the regenerated data, TACHIP with the aid of proper actuation program, can replicate some major features of the corresponding object. For instance, it can mimic low contact pressure when touching soft materials, such as paper towel and silicone. For rougher surfaces, such as abrasive paper, it can regenerate the vibrational stimuli with high amplitude, but relative low frequency compared to those smooth surfaces. For rigid materials, such as glass, TACHIP also can generate much higher actuation force, which is similar to the collected data.

### Subjective experiments for pneumatic array-based perception regeneration

Evaluation of actual haptic feedback performance of the proposed TACHIP is an important aspect to understand the gap between the intended regenerated perception and the actual perceived feeling about a specific object or event, and hence, to further improve the design of haptic feedback programs. However, most of the current subjective experiments can only record questionnaire-based statistical data which simply describe personal experiences. The real-time status of array-based haptic device for each feedback event usually lacks quantitative data, as the tests via external sensor or instrument may suffer mechanical distortion or displacement during the actual usage, rather than standard test setup. Thus, modification of different feedback patterns will consume a massive number of cyclic subjective experiments in conventional approach. Owing to the in-situ pressure sensor, as a methodology in establishing a more quantitative and objective evaluation standard, the sensing data can be simultaneously recorded for building database during the entire actuation process (also can be seen in Supplementary Fig. 16, Supplementary Note 6, and Supplementary Movie 2).

**Fig. 5:**
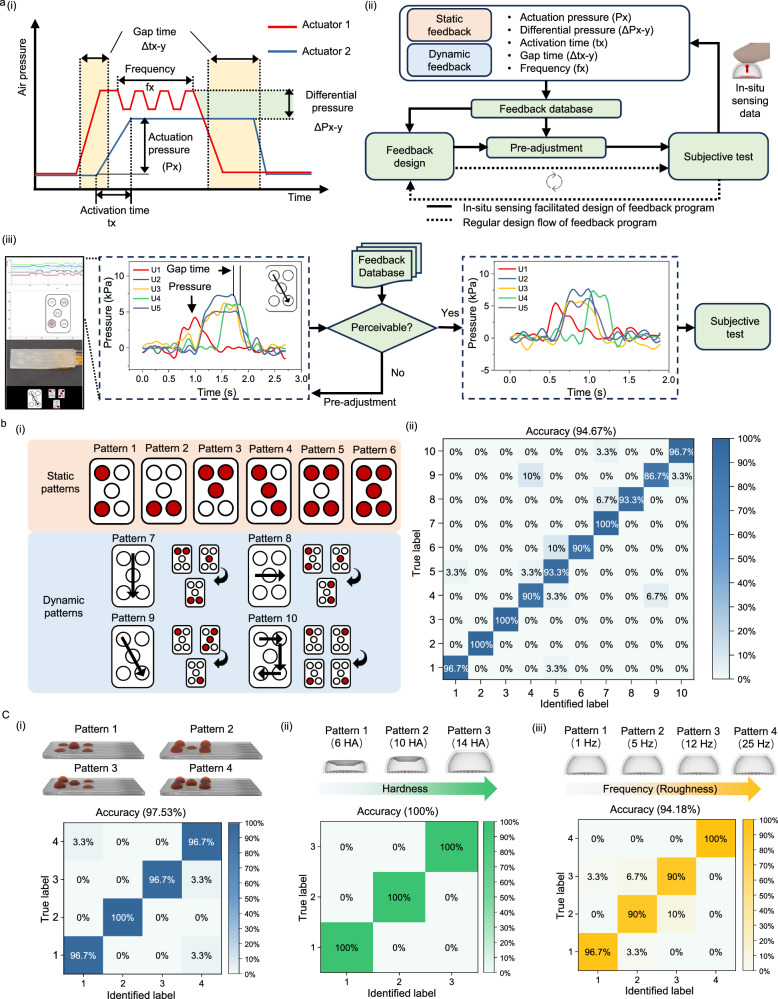
Subjective experiments for pneumatic array-based perception regeneration. **a** (i) Schematics of in-situ sensing facilitated feedback program design, left graph shows key parameters for affecting the actual user perception regarding the intended feedback pattern, (ii) a flow diagram describing the approach of utilizing pressure sensing database from previous subjective experiments for facilitating the design of new feedback program, with the comparison of regular design flow of haptic feedback pattern, (iii) practical test of utilizing in-situ sensing facilitated feedback program design. **b** (i) Static and dynamic patterns under constant input pressure for subjective experiments, (ii) confusion matrix of identification accuracy of ten patterns collected from ten participants. **c** Subjective experiments of pneumatic feedback patterns with varied control programs for mimicking (i) different geometries, (ii) different levels of hardness (measured by shore A durometer), and (iii) different levels of vibration frequencies or roughness, the confusion matrix of identification accuracy of each experiment is given below. Photo credit: Minglu Zhu, Soochow University.

In Fig. 5a, an optimized strategy of facilitating the design of a haptic feedback program is proposed with the aid of in-situ sensing. The purpose of tactile communication is to deliver spatiotemporal information of various stimulus. Meanwhile, according to the concept of exploratory procedures, which indicates purposive action patterns that perceivers execute to encode properties of surfaces and objects, It also claims the importance of spatiotemporal information in exploring objects or dynamic events. As an example of TACHIP, several key parameters are concluded from systematic analysis of the subjective test data and the corresponding sensing signals. Specifically, as shown in Fig. 5a(i), actuation pressure (P_x_, x is the index of actuator), differential pressure(ΔP_x-y_, x and y are the indexes of actuators), inflation time (t_x_), gap time (Δt_x-y_), and frequency (f_x_), etc., are essential for static and dynamic feedback. For instance, the smallest gap time of actuation between two actuators defines the limit of detection for user to perceive the sequence for dynamic feedback. Differential pressures among actuators cause variations of displacements among five pixels of TACHIP, and thus affect the perception regeneration of shape, geometry, etc. Feelings of texture and other dynamic events also depend on frequency of actuation, as skin mechanoreceptors are in charge of texture perception through different frequency stimuli related to action potential generated by ion transportation. As illustrated in Fig. 5a(ii), as an in-situ sensing facilitated feedback program design, during the subjective experiment, the sensing data of actuation will be simultaneously recorded and collected for establishment of feedback database. For those designed feedback patterns with low recognition accuracies, the abovementioned key parameters can be analyzed for modifying the program, and marked as the limit of design parameter. Eventually, with continuous improvement of database, more accurate pre-adjustment can be introduced before subjective test, and thus, to reduce the time-consuming cyclic subjective tests. As a practical test of the proposed in-situ sensing facilitated design of feedback program (Fig. 5a(iii)), the pattern 9 in Fig. 5b was redesigned based on feedback database. The in-situ pressure sensing data indicates that there are two possible issues existing in actuation program, including too small air pressure of unit 1, and too fast deflation of unit 4, which may cause unperceivable or undistinguishable differences of stimuli among those patterns with high similarities. Pre-adjustment was initiated prior to subjective test for facilitating the adjustment process, the pressure of unit 1 was increased and the deflation time of unit 4 was delayed for better reconstruction of spatiotemporal information of feedback pattern.

Eventually, some typical categories of haptic feedback were programmed with pre-adjustments and followed by subjective tests to validate the identification accuracies for the first-round test. In Fig. 5b(i), subjective experiments for actuations of static and dynamic patterns are illustrated, including six static patterns labeled as pattern 1 to pattern 6, and four dynamic patterns labeled as pattern 7 to pattern 10. Ten volunteers are involved in this blind test. Each pattern was activated three times, and a total of 30 rounds were conducted for each participant. Within 30 rounds, all ten patterns were randomly arranged in the program. According to the questionnaires recorded by all participants, a confusion matrix is shown in Fig. 5b(ii). Patterns 2, 3 and 7 show the highest identification accuracy, which is 100%. The lowest identification accuracy is observed on pattern 9, which has 10% of false identification with pattern 4. This is reasonable that both of two patterns have a stage of activating three actuators in a diagonal direction. The improper coding of the other two actuators for pattern 9 might cause insufficient feedback force and holding time for users to perceive, especially when there is a slight misplacement between TACHIP and finger, which is also mentioned above. According to this information, the modification of the program can be done for all haptic feedback patterns to ensure better performance. As an example, for a new haptic feedback pattern, the in-situ sensing data of each actuator is recorded during the initial activation and compared to database. If there are some key parameters showing high possibility of confusion (e.g., actuation pressure lower than perceivable threshold, gap time between two adjacent actuators is too short to be distinguished, etc.), the necessary modification of code (e.g., increasing of delay) can be conducted prior to the subjective test.

To further illustrate the advantages of pneumatic actuation with multiple tunable parameters, Fig. 5c presents results of subjective experiments regarding perception regeneration of shape or geometry, hardness, and roughness (frequency). In Fig. 5c(i), four patterns labeled as pattern 1, 2, 3, and 4, are designed to replicate the shapes of convex, concave, inclined, and flat surfaces. Three hardness levels were tested by changing the air pressure induced resistive force of pneumatic chamber, including hard, medium, and soft, are labeled as pattern 1, 2, and 3, as shown in Fig. 5c(ii). With the aid of piezo pump, test results of four actuation frequencies, including 1 Hz, 5 Hz, 12 Hz, and 25 Hz, marked as pattern 1, 2, 3, and 4, are used to mimic roughness or vibrations, as shown in Fig. 5c(iii). Additionally, more realistic stimulation can be perceived when simultaneous control of multiple parameters is adopted. For instance, roughness can be better rendered by collaboratively tuning the amplitude and frequency of the pneumatic actuation. According to three confusion matrices of subjective tests, TACHIP is showing promising performance in delivering multi-modal haptic feedback. With mixed actuation features, it allows higher immersivity with much more tactile information. The average identification accuracy of the first-round subjective test from different haptic feedback categories can reach about 95%, which show significant improvement compared to 85% in our previous reported works^12^. Overall, the key parameters of haptic actuators and the proper designed programs are essential to achieve high fidelity in human machine interactions. Although many current haptic devices cannot regenerate the whole profile of the real stimuli, the comprehensive evaluation of the actuation performances, including dynamic range, bandwidth, nonlinearity, etc., against the key information needed to be generated, will be an important topic to study.

### Reverse haptic feedback with pneumatic parallel manipulator for micro-motion projection

**Fig. 6:**
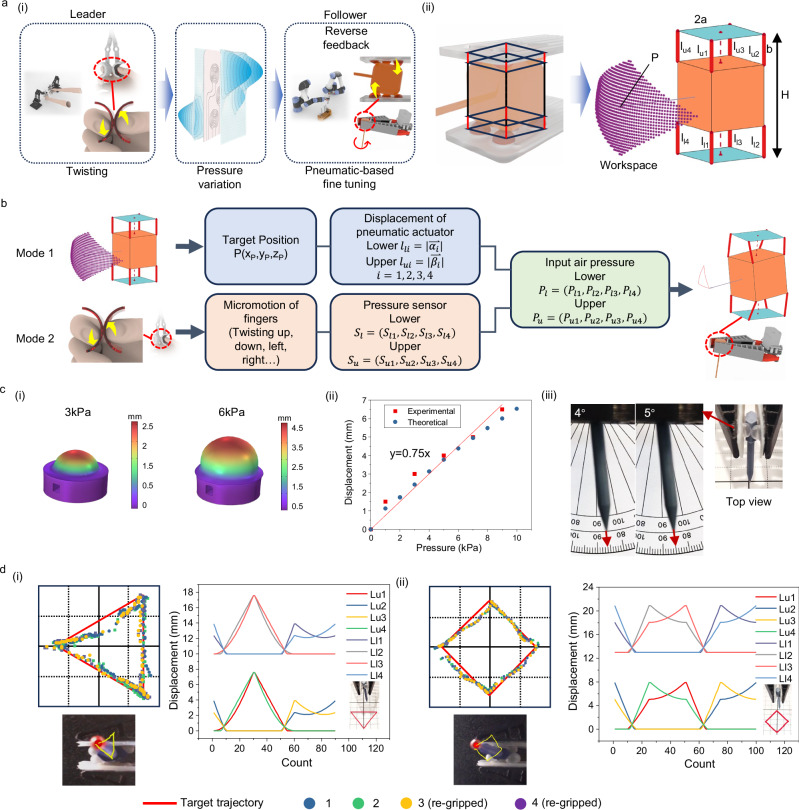
Reverse feedback with pneumatic parallel manipulator for micro-motion projection. **a** (i) Schematics of using pressure sensor array at leader side and pneumatic array at follower side for micro-motion projection, (ii) equivalent model and key parameters of pneumatic actuator array-based parallel manipulator with grabbed object, l_ui_ and l_li_ are the length parameters of pneumatic deformation heights of four actuators at upper and lower TACHIPs, respectively, H is the distance between two TACHIPs, b is the initial deformation height of all eight pneumatic actuators, 2a is the spacing between two adjacent working pneumatic actuators, P is the tip coordinates of the grabbed tool. **b** Flow diagram of two modes of utilizing TACHIP as teleoperated parallel manipulator, including mode 1: coordinate command-based control, and mode 2: projection of finger micro-motion from leader side to follower side as reverse feedback. **c** (i) Finite element analysis of pneumatic actuator under changing input pressure, (ii) comparison of experimental and theoretical data of the displacements against the input pressures, the linear fitting of the experimental data is given as red line with the corresponding equation, (iii) demonstration of resolution of micro-manipulation via TACHIP-based pneumatic parallel manipulator. **d** Comparison of target trajectories (red traces) and actual trajectories (colored dots) for manipulating the tip to form the trajectories of (i) triangle, (ii) square, blue and green dots represent two repeated manipulations for the first gripping, yellow and purple dots represent two repeated manipulations after re-gripping. The screenshots of the recorded trajectories (yellow traces) through visual recognition algorithm are given below. The original inverse kinematic data of displacements for each actuator are given at right side, with the inset photo of drawing the target trajectories. Photo credit: Minglu Zhu, Soochow University.

Conventional haptic feedback, or named as direct haptic feedback, is to deliver sensing information from robots to users for immersive experience about robot’s operation. However, reverse haptic feedback, as a specific term and technique used in this work, is to deliver user’s tactile information to robots for actuation or manipulation via the same TACHIPs used in direct haptic feedback. Differ from bilateral feedback, direct and reverse haptic feedbacks are operated asynchronously. Inspired by industrial Stewart parallel manipulator, the array of pneumatic actuators in two TACHIPs are utilized as micro manipulators for fine control of the grabbed object via the improved DOFs. As shown in Fig. 6a, inspired by dexterous control of objects via holding and twisting by human fingers, reverse haptic feedback is proposed. TACHIPs can act as both controller and manipulator to form a close-loop fine teleoperation system. According to Fig. 6a(i), a demonstrated fine teleoperation system consists of four TACHIPs attached to two control levers at leader side and two grippers at follower side. Once the grabbing command was sent by closing the control levers, two fingers are still able to perform micro motions, such as twisting, and thus, lead to variations of pressure distribution of each finger on its corresponding TACHIP. In Fig. 6a(ii), a sample tool grabbed by two TACHIPs is illustrated with labeled key parameters used for establishing a typical model of parallel manipulator.

The length parameters of l_u1_, l_u2_, l_u3_, l_u4_, l_l1_, l_l2_, l_l3_, and l_l4_ represent pneumatic deformation heights of four actuators (U1, U2, U4, U5) at upper and lower TACHIPs, respectively, H is the distance between two TACHIPs, and b is the initial deformation height of all eight pneumatic actuators. The parameter of 2a is the spacing between two adjacent working pneumatic actuators. Two center pneumatic actuators act as support pivots for upper and lower surfaces. The tip coordinates of the grabbed tool is set as P(x_p_, y_p_, z_p_). To understand the kinematics of this 3-RPS/US (revolute-prismatic-spherical joint/universal-spherical joint) parallel manipulator, the relationship between the pneumatic deformation heights of eight actuators and the target coordinates of the tip is studied. There are four platforms created by two TACHIPs and the contact surfaces on the grabbed tools, as shown in Fig. 6a(ii). The coordinates of platforms are presented as matrix *M*,

1 $$M=\left[\begin{array}{cc}{x}_{1} & \begin{array}{ccc}{x}_{2} & {x}_{3} & {x}_{4}\end{array}\\ {y}_{1} & \begin{array}{ccc}{y}_{2} & {y}_{3} & {y}_{4}\end{array}\\ {z}_{1} & \begin{array}{ccc}{z}_{2} & {z}_{3} & {z}_{4}\end{array}\end{array}\right]$$

and the corresponding platforms are marked as lower static platform (*M*_*sl*_), lower motional platform (*M*_*ml*_), upper static platform (*M*_*su*_), and upper motional platform (*M*_*mu*_). In addition, the final coordinates of lower and upper motional platforms are marked as *M*_*ml*_^*’*^ and *M*_*mu*_^*’*^, respectively. For any tip coordinates of the grabbed tool: P(x_p_, y_p_, z_p_), let:

2 $${\theta }_{z}=\arctan \frac{{y}_{P}}{{x}_{P}}$$

3 $${\theta }_{y}=\arctan \frac{{z}_{P}-\left(a+b\right)}{{x}_{P}}$$

Where θ is the rotation angle of the rod along the corresponding axis of the coordinate system. The final position of the lower motional platform is:

4 $${M}_{{ml}}^{{\prime} }=	 {M}_{{ry}}\left({\theta }_{y}\right)\cdot {M}_{{rz}}\left({\theta }_{z}\right)\cdot \left({M}_{{ml}}-\left[\begin{array}{cccc}0 & 0 & 0 & 0\\ 0 & 0 & 0 & 0\\ a+b & a+b & a+b & a+b\end{array}\right]\right) \\ 	+\left[\begin{array}{cccc}0 & 0 & 0 & 0\\ 0 & 0 & 0 & 0\\ a+b & a+b & a+b & a+b\end{array}\right]$$

The final position of the upper motional platform is:

5 $${M}_{{mu}}^{{\prime} }=	 {M}_{{ry}}\left({\theta }_{y}\right)\cdot {M}_{{rz}}\left({\theta }_{z}\right)\cdot \left({M}_{{mu}}-\left[\begin{array}{cccc}0 & 0 & 0 & 0\\ 0 & 0 & 0 & 0\\ a+b & a+b & a+b & a+b\end{array}\right]\right) \\ 	+\left[\begin{array}{cccc}0 & 0 & 0 & 0\\ 0 & 0 & 0 & 0\\ a+b & a+b & a+b & a+b\end{array}\right]$$

The following equations are set:

6 $${M}_{{ml}}^{{\prime} }-{M}_{{sl}}=({{\alpha }_{1}}^{ \rightharpoonup },{{\alpha }_{2}}^{ \rightharpoonup },{{\alpha }_{3}}^{ \rightharpoonup },{{\alpha }_{4}}^{ \rightharpoonup })$$

7 $${M}_{{mu}}^{{\prime} }-{M}_{{su}}=({{\beta }_{1}}^{ \rightharpoonup },{{\beta }_{2}}^{ \rightharpoonup },{{\beta }_{3}}^{ \rightharpoonup },{{\beta }_{4}}^{ \rightharpoonup })$$

Where ${\alpha }^{ \rightharpoonup }$ and ${\beta }^{ \rightharpoonup }$ are the pose vector expressions of the lower and the upper pneumatic actuator, respectively. The inverse kinematics solutions for the pneumatic deformation heights are set as follows:

8 $${l}_{{li}}=\left|{{\alpha }_{i}}^{ \rightharpoonup }\right|\,{{i}}=1,2,3,4 $$

9 $${l}_{{ui}}=\left|{{\beta }_{i}}^{ \rightharpoonup }\right|\,{{i}}=1,2,3,4 $$

The relationship to the pneumatic actuator displacements for the upper and lower actuators is derived from coordinate transformations of multiple platforms. The equations involve trigonometric functions and vector norms, resulting in transcendental equations that cannot be solved analytically due to their implicit nature. The inverse kinematics requires solving for displacements, which leads to a system of equations that are highly nonlinear because of the coupling between rotational and translational parameters, making analytical solutions intractable. The complexity is further illustrated by the workspace analysis, which requires iterative numerical approaches to map target coordinates to actuator displacements. To solve the non-linear equations, Newton downhill method, a variant of Newton’s method incorporating a global convergence strategy for numerical solutions, is used for direct kinematic solutions:

10 $${X}^{k+1}={X}^{k}-w{\left(J\left({X}^{k}\right)\right)}^{-1}f({X}^{k})\,(0 < {{{\rm{w}}}}\le 1)$$

Where *w* is downhill factor in the Newton downhill method, $J$ is Jacobian matrix of the objective function in the Newton downhill method, $f$ is objective function in the Newton downhill method. This approach balances computational efficiency with reliability, as it mitigates convergence issues common in standard Newton methods by dynamically adjusting the step size based on the norm of the residual function. As a result, a workspace of the proposed pneumatic actuator based parallel manipulator is shown in Fig. 6a(ii) (the detailed kinematic analysis can be seen in Supplementary Fig. 20, Supplementary Table 1, and Supplementary Note 8). By providing target coordinates, the corresponding displacements of pneumatic actuators and their required input air pressure can be calculated. This is defined as Mode 1 manipulation in Fig. 6b. Alternatively, Mode 2 is designed for more intuitive and real-time fine tuning of object during teleoperation (Fig. 6b). As previously mentioned, the finger twisting induced variation of pressure distribution can be directly projected into the input air pressure in corresponding pneumatic actuators at follower side, and thus, achieve micro manipulation with multiple DOFs. The concept of reverse feedback offers dexterous fine tuning of the object without modifying the actuation system of the main robotic platforms.

To validate the proposed approach, the relationship between input air pressure and the deformation of the pneumatic actuator is also investigated to ensure the feasibility of the manipulation. The finite element analysis (FEA) results are shown in Fig. 6c(i). Compared to the experimental data, the results indicate a relative good consistency with less than 10% error. According to Fig. 6c(ii), with the empirical equation defining the relationship between the deformation parameter and the input air pressure, the control program of pneumatic actuator arrays on TACHIPs can be developed to manipulate the grabbed tool. In Fig. 6c(iii), with the precise control of input air pressures through piezo pumps, the angular motion of the end point can be programmed with a resolution of about 1° (also can be seen in Supplementary Movie 3), which can complete various fine tuning tasks during collaborative teleoperations. To verify the motion DOFs of the proposed micro-manipulator, two operation paths are demonstrated within the defined workspace on a spherical surface, as depicted in Fig. 6d. According to the kinematics analysis, the corresponding displacements of the pneumatic actuators of two TACHIPs on the gripper for moving along the programmed trajectories can be calculated as shown in Fig. 6d(i) and d(ii). For each trajectory, the manipulations were repeated four times, including two cycles for the first gripping (blue and green dots), and two cycles after re-gripping (yellow and purple dots). The cyclic test for a single gripping shows relatively good repeatability, which also includes repeated distortions caused by several possible reasons, including non-linearity and fabrication tolerance of pneumatic actuators. On the other hand, the results of re-gripping tests show that the different distortions are observed due to slight changes of gripping status of tool, and the repeated trajectories after re-gripping are also showing good consistency. By considering compliance and non-linearity issues from elastomeric actuators, the proposed micro-manipulator encounters some limitations, such as high accuracy of motion, and small loading capacity (also can be seen in Supplementary Fig. 21). However, in many cases, together with human in the loop control shown in Mode 2 (also can be seen in Supplementary Movie 4), the reverse haptic feedback still possesses the features of tunability in multiple DOFs and the real-time adjustment in the whole teleoperation process.

### Demonstration of TACHIPs enhanced gross to fine teleoperation

**Fig. 7:**
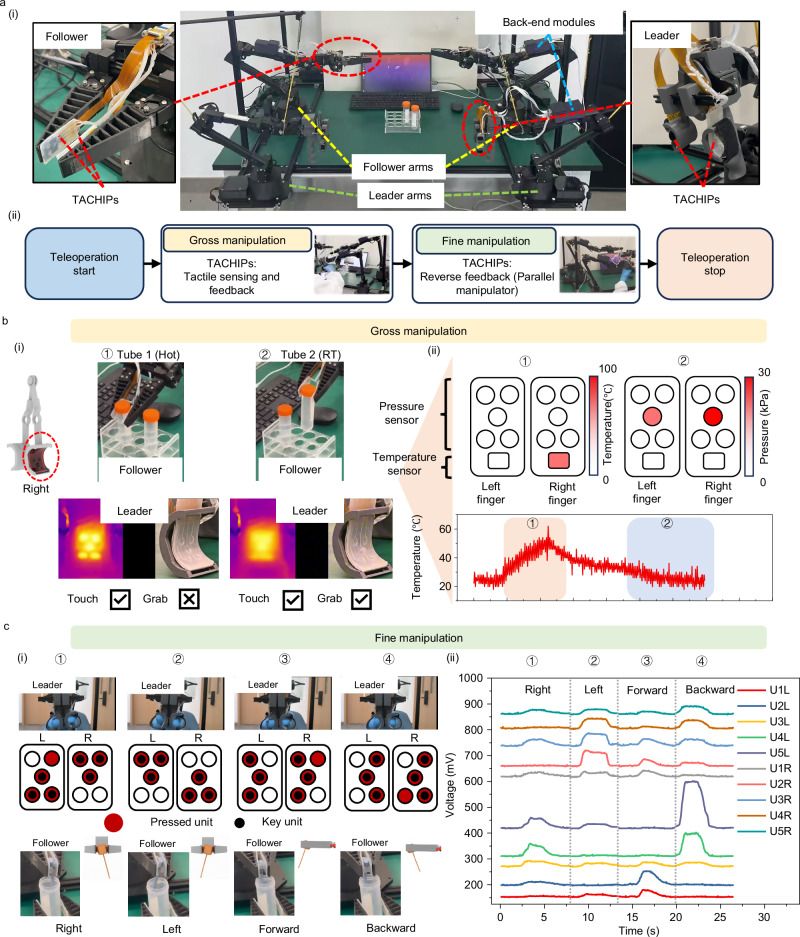
Demonstration of TACHIPs enhanced gross to fine teleoperation. **a** (i) Photos of setup of the teleoperation demonstration system with the enlarged view of TACHIPs at leader and follower sides, (ii) flow diagram of conduct gross to fine teleoperation with TACHIP assisted interactions. **b** Gross teleoperation stage: (i) thermal images and photos of TACHIPs at leader and follower sides for interacting with (1) hot and (2) room temperature tubes, (ii) the recorded pressure and temperature sensing data for two events: (1) touch hot test tube without grabbing, and (2) touch room temperature test tube and grabbing. **c** Fine teleoperation stage: demonstration of micro-motion projection with mode 2 for manipulating the grabbed object in four directions: (1) right, (2) left, (3) forward, and (4) backward. (i) Top: photos of finger micro-motion at leader side. Middle: the schematics of the pressed sensors (red circle) and the actual key units (black dot) for identifications of different directions. Bottom: the corresponding micro-manipulation of the grabbed object at follower side via TACHIPs, with the schematics for better visualization of moving directions, (ii) the recorded in-situ pressure monitoring data of finger micro-motion at leader side, manual offsets are given for illustration of multiple signals, U1L, U2L, U3L, U4L, U5L represent U1, U2, U3, U4, U5 of left TACHIP at leader side handle, and U1R, U2R, U3R, U4R, U5R represent U1, U2, U3, U4, U5 of right TACHIP at leader side handle. Photo credit: Minglu Zhu, Soochow University.

As shown in Fig. 7a(i), the open-source project ALOHA: A Low-cost Open-source Hardware System for Bimanual Teleoperation developed by researchers from Stanford University was utilized as reference to build the hardware platform for collaborative teleoperation system^35^ (also can be seen in Supplementary Fig. 22 and Supplementary Note 9). In Fig. 7a(ii), a flow diagram shows the complete process of conducting gross to fine teleoperation, and the typical roles of TACHIPs are also highlighted with the featured functions. For the first stage (gross teleoperation stage) shown in Fig. 7b, user conducted the teleoperation to select and grab the room temperature test tube. The corresponding screenshots of interacting with hot and room temperature test tubes can be seen in Fig. 7b(i), and the pressure and temperature sensing data for these two events are shown in Fig. 7b(ii). TACHIPs were acting as tactile communication tool via temperature and pressure sensing (follower side) and feedback (leader side) capabilities.

Figure 7c shows the process of conducting fine teleoperation through Mode 2 based reverse feedback of TACHIPs, with the recorded pressure sensing data of finger micro motions from leader side. As can be seen from the multi-channel signals from two TACHIPs, the actual amplitude of each sensor may experience certain variations due to several aspects (Fig. 7c(ii)), such as inconsistencies of finger placements and applied forces from users. To ensure the stability of fine teleoperation (micro manipulation), the comparison algorithms were used to assist the direction identification of actual finger mirco-motions. As shown in Fig. 7c(i), the key units (key pixels) for identifying the directions of the finger twisting motions are highlighted as black dots. The pressed units (marked as red) from the real experimental results indicate that the additional units are touched unintentionally due to the abovementioned reasons. By matching the pressed units with the key units, the twisting direction can then be determined and projected into the pneumatic actuators of TACHIPs at follower side via reverse feedback. In addition, the threshold of pneumatic actuators is also set to avoid the potential damage caused by overloaded forces, such as the signal from U5L channel during backward manipulation shown in Fig. 7c(ii). In general, the actual workspace is defined in Fig. 6a(ii).

**Fig. 8:**
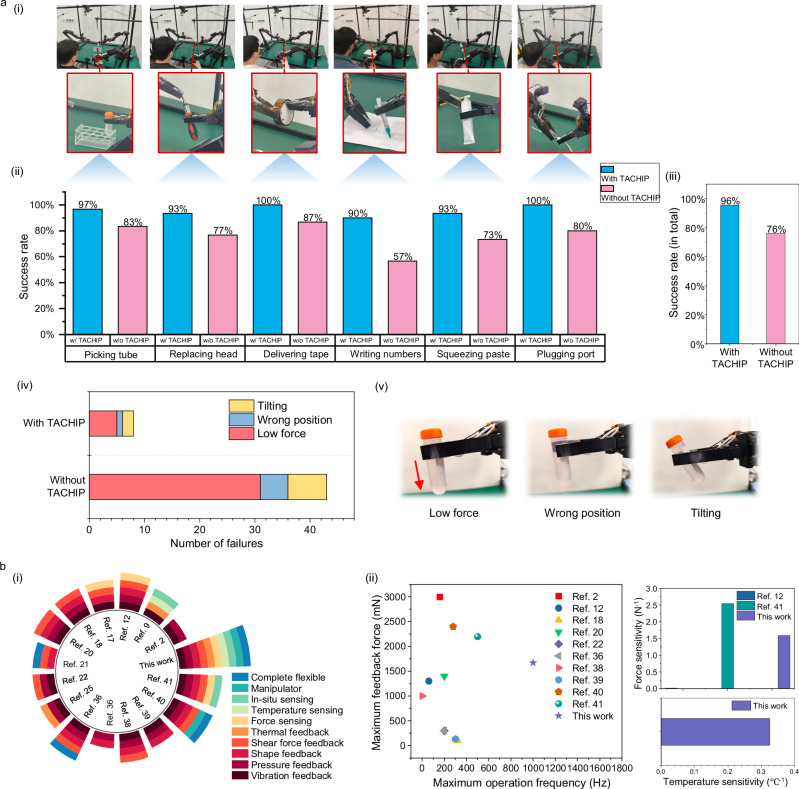
Evaluation of TACHIP assisted teleoperation tasks. **a** Comparisons of success rates of different teleoperation tasks performed with and without TACHIPs. (i) Real photos of the setup and the zoom-in views of six tasks, including picking and placing of tube, replacing head, deliver tape, writing number, squeezing paste, and plugging port, (ii) the results of success rates comparisons for the corresponding teleoperation tasks with and without TACHIP, (iii) comparison of success rates with and without TACHIP in total, (iv) Statistical data of major failure conditions during the above teleoperations, (v) illustrations of the major failure conditions. **b** Comparisons of (i) the functionalities and (ii) the general performances (maximum feedback force, maximum operation frequency, force sensitivity, and temperature sensitivity) with the reported works (Ref. ^2^, Ref. ^9^, Ref. ^12^, Ref. ^17^, Ref. ^18^, Ref. ^20^, Ref. ^21^, Ref. ^22^, Ref. ^25^, Ref. ^36^, Ref. ^37^, Ref. ^38^, Ref. ^39^, Ref. ^40^, Ref. ^41^). Photo credit: Minglu Zhu, Soochow University.

To evaluate the effectiveness of TACHIPs in improving the success rate of typical teleoperation tasks, the comparison experiments were conducted as shown in Fig. 8. A total of six different tasks were designed, including pick and place of tube, replace head, deliver tape, write number, squeeze paste, and plug USB. Six participants were selected as operators. For each task, each participant repeated the operation five times with TACHIP and followed by five times without TACHIP. Overall, there are 30 rounds of operations with TACHIP and 30 rounds of operations without TACHIP in total for each task. For the condition of dropping the objects, or trying more than three times, it is defined as failure. As can be seen in Fig. 8a(i), the real photos of teleoperation tasks are given. The comparisons of success rates of the corresponding teleoperation tasks performed with and without TACHIP are illustrated in Fig. 8a(ii). It is obvious that TACHIPs can drastically improve the success rates in teleoperation, especially for those tasks which require multiple DoFs and better control of forces. A general improvement of about 20% increase in success rate is depicted in Fig. 8a(iii). Furthermore, the detailed failure conditions are categorized into three main types, and the statistical data is shown in Fig. 8a(iv), with typical examples illustrated in Fig. 8a(v).

Overall, a comprehensive illustration of the major contributions of the proposed devices is presented by the benchmarking of the functionalities and the general performances shown in Fig. 8b^2,9,12,17,18,20–22,25,36–41^. Specifically, a complete flexible TACHIP is developed with both force and thermal feedback, as well as in-situ sensing capabilities. The features of in-situ sensing and pixel distribution of TACHIP allow self-adaptive, self-monitoring feedback with objective evaluation function, as well as parallel manipulator inspired micro-manipulator for fine teleoperation (Fig. 8b(i)). While the TACHIP still maintains comparative haptic feedback performance, with a maximum output force of 1.67 N and operation frequency range of 0-500 Hz (Fig. 8b(ii)). Detailed information also can be seen in Supplementary Table 2.

## Discussion

In the foreseeable future, robotic teleoperation may become increasingly important to industry, homecare, education, and special operations. Ranging from gross to fine teleoperations, sensing and feedback of tactile and micro-motion information will be expected for comprehensive understanding about operation status. The proposed TACHIP featured with the in-situ pressure and temperature sensor designed within the haptic actuator structure offers fusion of multi-modal sensing and feedback with high spatial consistency. Specifically, pneumatic actuator of TACHIP is capable of operating at the frequencies range from 0 to 1000 Hz, with a maximum output force of 1.67 N. While it also can detect the applied force with a sensitivity of 1.59 N^−1^. The liquid metal-based heater provides tunable and selective thermal feedback with a response time of about 10 sec from 0 to 45 °C, and the pectin temperature sensor offers a temperature sensitivity of 0.325 °C^−1^. Overall, there are several main contributions. First, owing to simultaneous SA and FA stimulations, as well as in-situ sensing function, multi-modal and self-adaptive pneumatic feedback can be realized to perform dynamic modulation of feedback actuation for more realistic perception about object properties. Secondly, as an innovative methodology in establishing a more quantitative and objective evaluation standard for the above mentioned haptic feedback functions, the in-situ sensing signals of different feedback patterns can help to generate the database which can facilitate the design of haptic feedback algorithm with higher identification rate within fewer rounds of subjective tests. Finally, fine tuning of object orientation is a key issue for precise teleoperation. By investigating potentials of pneumatic actuators and in-situ pressure sensors on TACHIP, a reverse tactile communication which project finger micro-motions from leader side into follower side for fine tuning of the target object was demonstrated. The actuator array is utilized as a parallel manipulator which enables more DOFs to the conventional robotic gripper and achieve further fine manipulation during teleoperation. As a system level integration for practical application, TACHIP with FPC can achieve simple plug-in to conventional PCB for quick deployment. Furthermore, With the developed wireless portable system, TACHIPs can act as convenient accessories to most of current robot platforms and wearable HMIs, without changing their main infrastructures. On the other hand, there are still some limitations exist due to the current materials and fabrication process. Multiple TACHIPs can provide a certain scalability, but the fabrication of large area devices with dense pixels is still challenging. The reverse haptic feedback-based micro manipulation lacks high accuracy of motion control, attributed to compliance and nonlinearity of the elastomeric actuator. Overall, the proposed work is still showing the possibilities in promoting the haptic devices along with this evaluation methodology in various fields, including industrial productivity, education, laboratory, healthcare, etc.

## Methods

### Fabrication of liquid metal pressure sensor

Masks of microchannel pattern for photolithography were designed and fabricated by laser. The glass slide substrate was repeatedly cleaned with acetone and alcohol, followed by ultrasonic cleaning for 3 min. The glass slide was dried with nitrogen gas and placed on a 120 °C hotplate for 20 min. Attached photoresist dry film (DuPont) to deionized (DI) water rinsed glass slide and then pressed and bonded it tightly using a laminating machine. Afterward, removed the protective film of the dry film on glass slide, and firmly attached to the mask. Placed the glass slide under a UV lamp for exposure of 6 sec. The unexposed part was washed away with a 1.5% Na_2_CO_3_ solution, and gently rinsed with DI water, dried with nitrogen gas. Baked the slide at 50 °C for 30 min to obtain the microchannel mold. For better peeling off process of the polydimethylsiloxane (PDMS) film with microchannel, silanization treatment of the glass slide was done before spinning coating of PDMS. Placed the glass slide in a vacuum chamber, and then added 100 μL of dimethylchlorosilane into the vacuum chamber. Evacuated for 3 min and placed for 10 min.

Mixed PDMS and curing agent (184 Sylgard, Dow-corning) with weight ratio of 10:1. Spin coated it onto the microchannel mold at 300 RPM for 30 sec, then placed it in a vacuum chamber for degassing of 30 min. Transferred it into an oven to cure at 70 °C for 2 h. Peeled off the PDMS film from the glass slide, and drilled injection hole at the end of microchannel with needle. Spin coated another layer of blank PDMS film on glass slide at 800 RPM for 30 sec, cured it at 70 °C for 2 h, and obtained the blank PDMS film. Used tape to clean the surfaces of two PDMS films, then placed them in a plasma cleaner (PDC-MG, Mingheng), treated with the power of 10 W and the oxygen of 8−10 NI/h for 120 sec. After treatment, bonded two PDMS films together and transferred the bonded film onto a heating plate at 80 °C for two h. Used a syringe to inject liquid metal into microchannels. Cut the end part of injection region, and inserted the customized flexible print circuit (FPC) into the microchannels, and encapsulated by silicone epoxy.

### Fabrication of pneumatic actuator array

Mold of pneumatic actuator array was 3D printed and polished to ensure surface quality. Mixed silicone solution A and B (Ecoflex 00-30, smooth-on) with weight ratio of 1:1 and stirred for 3 min. Poured the mixture of silicone into the 3D printed mold, placed it into an vacuum chamber for degassing of 10 min and transferred it into an oven at 50 °C for 2 h. After solidification, pneumatic actuator array was peeled off from the mold. Silicone was applied at the surface with pneumatic chambers and air channels for bonding with PDMS thin film. Mixture of silicone solution was also prepared and applied around the edge of the bonding interface for better encapsulation. Soft air tubes were inserted into the air channels accordingly and sealed by silicone rubber (E43 Elastosil).

### Fabrication of pectin temperature sensor

A 2 wt% pectin solution with 32 mM CaCl_2_ and 0.3 wt% Xanthan was prepared. For 100 g solution, 2 g of pectin powder from citrus peels (Cool chemistry), 0.355 g of CaCl_2_ (Cool chemistry), 0.3 g Xanthan powder (Yuwanbang) were measured. Placed the pectin powder into a beaker. Added DI water into the beaker and stirred at 1400 RPM and 80 °C until the pectin was completely dissolved to form a homogeneous solution. After cooled down to 40 °C, added the weighed Xanthan powder and CaCl_2_ to the solution and stirred until it is completely dissolved. Poured the solution into a petri dish and placed into an oven for dehydration. Film was the formed and peeled off by razor. Silver electrodes was then screen printed and dried in the oven. After dicing, placed pectin thin film on surface of pneumatic actuator array and encapsulated by additional silicone.

### Design of processing module for sensing and feedback

Processing module of sensing and feedback was developed as an integrated printed circuit board (PCB) consists of customized microprocessor (based on Arduino MEGA 2560), bluetooth module (HC-05), pressure and temperature sensing circuit, piezo pumps (MZB3004T04, Murata) with driver circuits, and a lithium battery. FPC connector was also included in PCB for quick setup of TACHIP with FPC. For air tubes of pneumatic actuators, a 3D printed hub was designed for easy plug-in of multiple tubes.

### Characterization of liquid metal pressure sensor

Characterizations of sensitivity, responsivity, and hysteresis of pressure sensor were conducted using force tester (Multitest 2.5-i, Mecmesin). Oscilloscope (MSO54B, Tektronix) and electrometer (6514, Keithley) were used to measure and record the characterization data. The test head was programmed with a contact speed of 900 mm/min. A voltage divider circuit was applied to measure the resistance change of sensor via microprocessor in demonstration. The ΔV/V₀ represent resistance change from the liquid-metal-based in-situ sensor. The data presented with pressure unit of kPa were calibrated by force tester (Multitest 2.5-i, Mecmesin).

### Characterization of pneumatic actuator

Characterizations of actuation force of pneumatic actuator were done with force tester (Multitest 2.5-i, Mecmesin). The corresponding input air pressure was recorded by manometer (SW-512B, SNDWAY). Displacement and shape profiles were measured using laser profiler (MVEB-L-3D, ATCHBEST). Vibration frequency and amplitude were measured by Laser Doppler Vibrometer (4525 R, Kathmatic). Monitoring of Slow-adaption (SA) and Fast-adaption (FA) tactile information were performed by commercial thin film piezoelectric vibration sensor (LDTM-028K, Te) and piezoelectric pressure sensor (FS-ARR, Legact).

### Characterization of thermal feedback

Thermal feedback performance was characterized by commercial thermistor and thermal imager (PTi120, Fluke) for both data and image recording. The input power of corresponding feedback temperature was monitored by digital DC power supply.

### Characterization of pectin temperature sensor

Pectin temperature sensor was placed on a digital hotplate with controllable heating temperature. For each temperature, the data was recorded after 1 min, and the actual surface temperature was measured by infrared thermometer (DL333380A, DELI). For object contact test, temperature sensor was placed on a rubber sheet, and the object with different temperatures were placed or pressed onto the sensor sequentially. The continuous monitoring data was recorded by microprocessor (Arduino MEGA 2560) with pre-processing circuit.

### Reliability test of sensing and feedback

Cyclic inflation test was conducted continuously for 24 h, with an operation frequency of 30 cycles/min and a fixed input power of pump. In-situ sensor was applied for continuous monitoring of cyclic inflation. A standard manometer was used as a reference sensor for verifying the stabilities of the sensor and the actuator. Test of temperature effect was done by placing TACHIP on a tunable heater, and the actual temperature was verified by thermal imager (PTi120, Fluke). Test of humidity effect was done by placing TACHIP into a container with humidifier, and the relative humidity was monitored by humidity sensor.

### Subjective experiments for pneumatic haptic feedback

Subjective experiments for haptic feedback patterns are labeled as Pattern 1 (P1), Pattern 2 (P2)…, etc. Ten participants are involved in this blind test. For each volunteer, each pattern was activated three times in total, but will be randomly arranged with other patterns together. For example of two patterns test, participant A will be tested as: P1 → P2 → P1 → P1 → P2 → P2, participant B will be tested as P2 → P2 → P1 → P1 → P1 → P2, etc. Therefore, for each participant, the test program was designed individually. Overall, each pattern was tested 30 times by ten participants. To ensure the quality and duration of generated patterns were sufficient for participants to perceive, each pattern was set to active for 5 sec, and followed by 10 sec of gap before the next pattern. During the gap time, the participants marked down the identified pattern of the previous round on questionnaire. Finally, the statistical data were obtained and evaluated based on patterns and participants. All of ten participants have signed the informed consent forms which described the research purpose, confidentiality, rights, use of results, etc.

### TACHIP-enabled micro manipulation

The central pneumatic actuator of TACHIP acts as pivot of grabbing the tools and supporting the multiple DoFs motions of the grabbed tools. To achieve smooth control, two methods of increasing the stiffness of the central actuator were adopted, including the usage of the higher Young’s modulus elastomer for central actuator, and the additional attachable pivot for bonding onto the central actuator. At current stage, all of the mode 1 based micro-manipulations were done as open-loop control, which was programmed by model calculation described in supplementary information. The real trajectory tracking was done by applying OpenCV visual tracking algorithm to process the recorded video.

### Teleoperation system for demonstration

The open-source project ALOHA developed by researchers from Stanford University was used as reference to build the hardware platform for collaborative teleoperation system. Main components of this system include two follower robots (Widow 250 Robot Arm 6DOF), two leader robots (ViperX 300 Robot Arm 6 DOF), and a computer installed with Ubuntu and ROS system. The gripper side of leader and follower robots were customized with 3D printed accessories. Fin ray soft grippers with TACHIPs were installed at follower side. Control levers with TACHIPs were installed at leader side for users to perform grab and release action. Back-end processing modules were attached to arms of robots. For safety precautions, the maximum and minimum forces applied by robotic grippers during this demonstration were measured. According to the measured force range, the force projection ratio between sensing and feedback was determined as 5:1. The dynamic range of the pneumatic actuator was set as F_max_/F_min_ = 500 mN/100 mN. The saturation value was set as 450 mN.

### Evaluation of TACHIP-assisted teleoperation system

To evaluate the improvement in success rate of teleoperation, tests of teleoperations were conducted by selecting six participants to perform six classic teleoperation tasks. For each task, the participant was asked to perform teleoperation five times with TACHIP and five times without TACHIP. The number and the reasons of failures were recorded for statistical analysis.

## Supplementary information

Supplementary Information

Description of Additional Supplementary Files

Supplementary Movie 1

Supplementary Movie 2

Supplementary Movie 3

Supplementary Movie 4

Transparent Peer Review file

## Data Availability

The experimental data and the schematics of print circuit board generated in this study have been deposited in the figshare database under accession code (10.6084/m9.figshare.31842847).

## Code availability

The sensing and feedback codes and MATLAB codes that support the findings of this study have been deposited in the figshare database under accession code (10.6084/m9.figshare.31842847).
