# Supplementary material for: Soft tactile chip with in-situ sensing for haptic rendering and reverse feedback enhanced gross to fine teleoperation
Source: Nat Commun. 2026 May 11;17:6295. doi: 10.1038/s41467-026-73000-8 (PMC13376641; doi:10.1038/s41467-026-73000-8)
Supplement: Supplementary file 1 — Supplementary Information [file 41467_2026_73000_MOESM1_ESM.pdf]

Supplementary Information for

Soft tactile chip with in-situ sensing for haptic rendering and reverse feedback enhanced gross to fine teleoperation

Minglu Zhu, Hao Ling, Rui Wang, Zhanpeng Du, Qi Sun, Ke Chen, Kuihan Chen, Lining Sun, Cheng Fan, Chengkuo Lee*, Xuan Li*, Tao Chen*

*Corresponding author: elelc@nus.edu.sg (C.K.L.); xuanli@suda.edu.cn(X.L.); chent@suda.edu.cn (T.C.)

Supplementary Note 1. Fabrication method to TACHIP
Supplementary Note 2. Characterization data of pressure sensor in TACHIP
Supplementary Note 3. Object recognition realized by distributive TACHIPs in soft robotic gripper
Supplementary Note 4. Characterization data of pneumatic actuator in TACHIP
Supplementary Note 5. Teleoperation with dynamic sensing and haptic feedback functions
Supplementary Note 6. Design of subjective experiment for haptic feedback
Supplementary Note 7. Approximation of temperature distribution via sensing fusion
Supplementary Note 8. Kinematic analysis of TACHIP enabled parallel manipulator
Supplementary Note 9. Teleoperation system used for demonstration
Supplementary Fig. 1. Fabrication process of TACHIP.
Supplementary Fig. 2. Characterization of hysteresis data of liquid metal-based pressure sensor
Supplementary Fig. 3. Temperature effect of liquid metal-based pressure sensor under varied loadings
Supplementary Fig. 4. Stability test of liquid metal-based pressure sensor under cyclic loading
Supplementary Fig. 5. Repeatability test of pressure sensor under varied loading forces for three times
Supplementary Fig. 6. Comparison of the real applied forces and the forces monitored by the in-situ pressure sensor
Supplementary Fig. 7. In-situ air pressure monitoring data via pressure sensor during pneumatic actuation for two different inflation and deflation processes
Supplementary Fig. 8. Recognition of grabbed object with three TACHIPs
Supplementary Fig. 9. In-situ air pressure monitoring for different inflation and deflation processes
Supplementary Fig. 10. Comparison of pneumatic feedback forces with different materials and sizes
Supplementary Fig. 11. In-situ sensing data for monitoring of low frequency pneumatic actuation and hysteresis test.
Supplementary Fig. 12. Influences of environmental variations and crosstalk to the in-situ pressure sensor and the pneumatic actuator
Supplementary Fig. 13. Reliability test for both in-situ pressure sensing and pneumatic actuator under cyclic operations
Supplementary Fig. 14. System latency between sensing and feedback
Supplementary Fig. 15. Teleoperation with dynamic sensing and haptic feedback functions

Supplementary Fig. 16. Identification accuracy of subjective experiment
Supplementary Fig. 17. Experimental data of dynamic thermal feedback for regenerating thermal perception
Supplementary Fig. 18. Finite element analysis of liquid metal-based heater via supplied voltage
Supplementary Fig. 19. Schematics of approximation of temperature distribution for thermal sensing and feedback
Supplementary Fig. 20. Displacements of the corresponding pneumatic actuators for completing the trajectories
Supplementary Fig. 21. Deflections of manipulated object under the changing external loadings
Supplementary Fig. 22. Photo of TACHIPs enhanced gross to fine teleoperation system
Supplementary Table 1. Definitions of parameters shown in kinematic analysis
Supplementary Table 2. Comparisons of functionalities and performances of haptic feedback interfaces

Supplementary Note 1. Fabrication method to TACHIP

Fabrication of liquid metal pressure sensor

Masks of microchannel pattern for lithography were designed using AutoCAD software, and fabricated by laser. Glass slides, as substrate of photoresist film, were repeatedly cleaned with acetone and alcohol three times, followed by ultrasonic cleaning for 3 minutes. Then, the glass surface was dried with nitrogen gas and placed on a 120°C hotplate for 20 minutes to evaporate surface moisture and keep the glass surface dry. Took a piece of photoresist dry film (DuPont) fit with the size of the glass. Removed the protective film on one side and attached it to deionized water rinsed glass slide, and then pressed it tightly using a laminating machine. Afterward, removed the protective film on the other side to obtain a dry film attached glass slide. Glass slide with the dry film was closely attached to the mask by clamps, and then placed under a UV lamp with the mask side facing up for exposure of 6 seconds. Photosensitive dry film is a type of negative photoresist. The unexposed part was washed away with a 1.5% sodium carbonate solution, then gently rinse with deionized water, and dried with nitrogen gas, and placed it in an oven at 50°C for 30 minutes obtain the microchannel mold structure. To ensure easy peeling off process for the polydimethylsiloxane (PDMS) film with microchannel from the glass slide, silanization treatment of the dry film glass slide was done before spinning coating PDMS. Placed the channel mold and petri dish in a vacuum chamber, then used a pipette to add 100μL of dimethylchlorosilane into the vacuum chamber. Evacuated for 3 minutes and placed for 10 minutes to allow the volatilized silane to settle on the glass surface, facilitating subsequent peeling of PDMS from the mold surface.

Mixed PDMS and curing agent (184 Sylgard, Dow-corning) with weight ratio of 10:1, 15:1, and 20:1. Spin coated it onto the microchannel mold at 300r/min for 30 seconds, then placed it in a vacuum chamber for degassing of 30 minutes. Transferred it into an oven to cure at 70°C for 2 hours. Peeled off the PDMS film from the glass slide, and drilled injection hole at the end of microchannel with needle. Spin coated a layer of blank PDMS film on glass slide at 800r/min for 30 seconds, cured it at 70°C for 2 hours, and then, peeled off the blank PDMS film. Used tape to clean the surfaces of two PDMS films, then placed them in a plasma cleaner (PDC-MG, Mingheng), treated with the power of 10W and the oxygen of 8-10 NI/h for 120 seconds. After treatment, bonded two PDMS films together and transferred the bonded film onto a heating plate at 80°C for two hours. Used a syringe to inject liquid metal into microchannels. Cut the end part of injection region later, and inserted the customized FPC into the microchannels, and encapsulated by silicone epoxy.

Fabrication of pneumatic actuator array

Mold of pneumatic actuator array was 3D printed and polished to ensure surface quality. Mixed silicone solution A and B (Ecoflex, smooth-on) with weight ratio of 1:1 and stirred for 3 minutes. Poured the mixture of silicone into the 3D printed mold, placed it into an vacuum chamber for degassing of 10 minutes and transferred it into an oven at 50°C for 2 hours. After solidification, pneumatic actuator array was peeled off from the mold. Silicone epoxy was applied at the surface with pneumatic chambers and air channels for bonding with PDMS pressure sensor. Mixture of silicone solution was also prepared and applied around the edge of the bonding interface for better encapsulation. Soft air tubes were inserted into the air channels accordingly and sealed by silicone epoxy.

Fabrication of Pectin temperature sensor

A 2 wt% pectin solution with 32 mM $CaCl_2$ and 0.3 wt% Xanthan was prepared. For 100 g solution, 2 g of pectin powder from citrus peels (Cool chemistry), 0.355 g of $CaCl_2$ (Cool chemistry), 0.3 g Xanthan powder (Yuwanbang) were measured. Placed the pectin powder into a beaker. Added DI water into the beaker and stirred at 1400 RPM and 80 °C until the pectin was completely dissolved to form a homogeneous solution. After cooled down to 40 °C, added the weighed Xanthan powder and $CaCl_2$ to the solution and stirred until it is completely dissolved. Poured the solution into a petri dish and placed into an oven for dehydration. Film was the formed and peeled off by razor. Electrodes was then screen printed with silver paste, and followed by drying in the oven. After dicing, placed pectin thin film on surface of pneumatic actuator array and encapsulated by additional silicone.

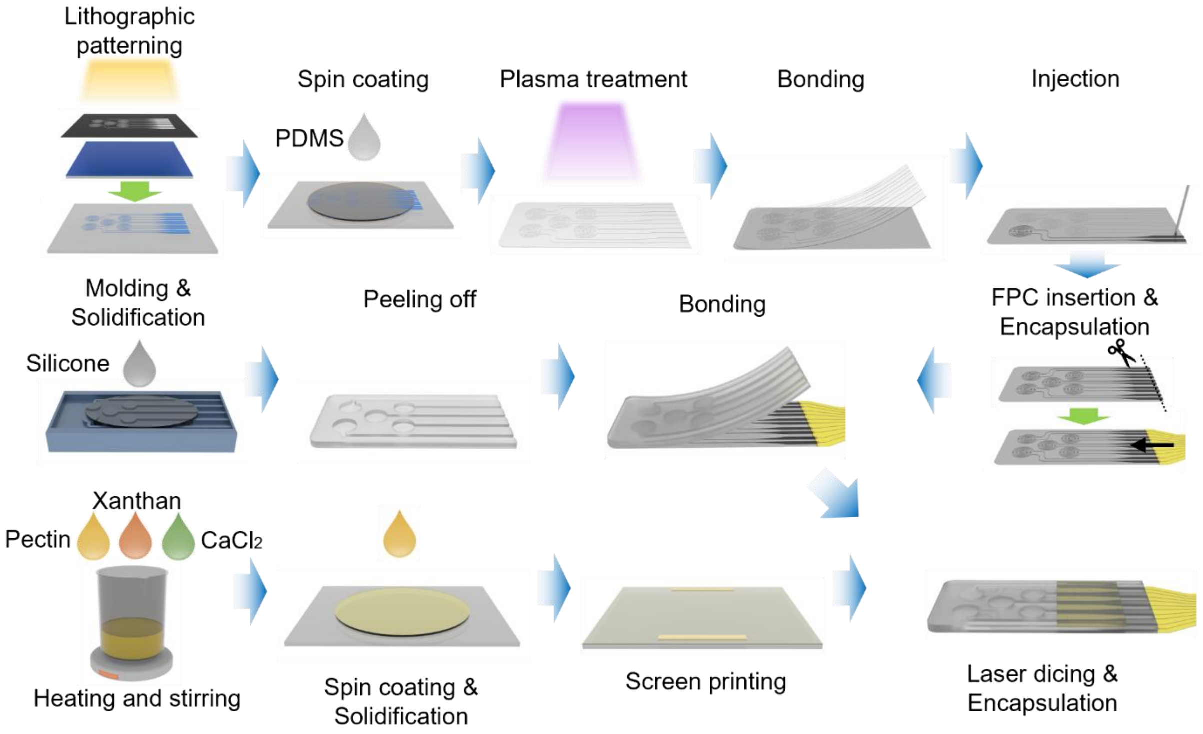

Supplementary Fig. 1. Fabrication process of TACHIP.

Supplementary Note 2. Characterization data of pressure sensor in TACHIP

In Supplementary Fig. 2, hysteresis tests based on variation of microchannel widths and turns of coil-shaped microchannel. Supplementary Fig. 3 gives the experimental results about temperature effect of liquid-metal based pressure sensor. Supplementary Fig. 5 shows a repeatability test of pressure sensors when the force gauge applied varied forces sequentially to the sensor, and removed the load by similar steps. The same test was repeated three times to prove the overall stability. Supplementary Fig. 6 shows the comparison of the applied and the sensed forces by TACHIP, the deviation of the sensed forces in Supplementary Fig. 6a was caused by the influence of the silicone layer to the delivery of mechanical stimuli. Supplementary Fig. 7 illustrated the pressure distribution sensing signals for different contacts with uniform loading. The experimental results indicate the performance consistency of multi-pixel sensors. Supplementary Fig. 9 is the in-situ air pressure monitoring data via pressure sensor during pneumatic actuation. Two actuation procedures were conducted, including continuous inflation and deflation, as well as stepwise inflation and deflation. The pressure sensing signals of two actuation

procedures are consistent with the actual program designed for these two tests. Hence, the proposed in-situ monitoring strategies for pneumatic haptic feedback are reliable, and a close-loop sensing and feedback system is completed, which is the base of realizing advanced haptic feedback, such as self-adaptive pneumatic haptic feedback for hardness rendering.

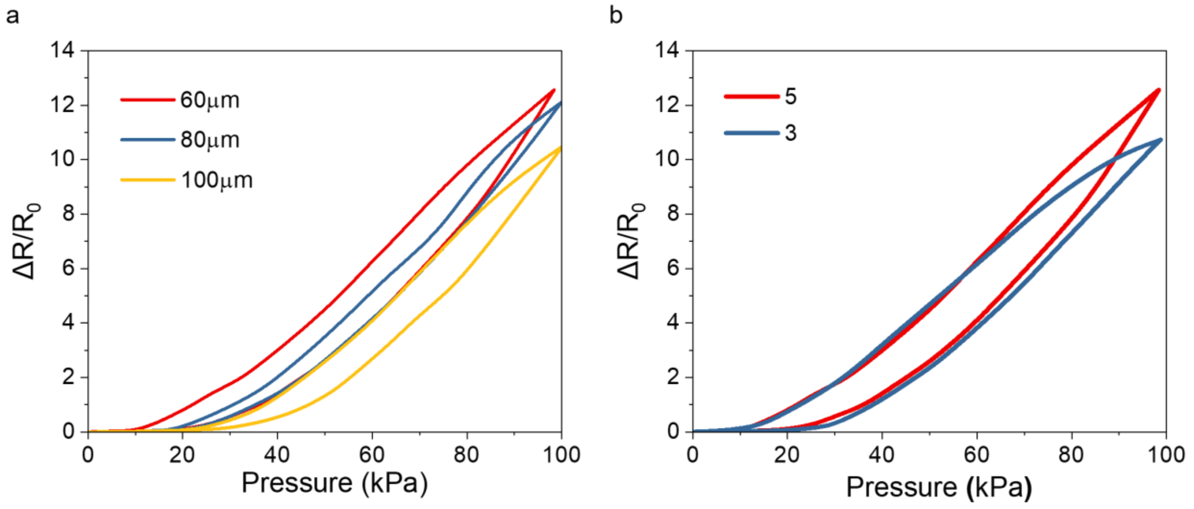

Supplementary Fig. 2. Characterization of hysteresis data of liquid metal-based pressure sensor. **a** Experimental data of liquid metal micro-channel with different widths. **b** Experimental data of liquid metal micro-channel with different widths.

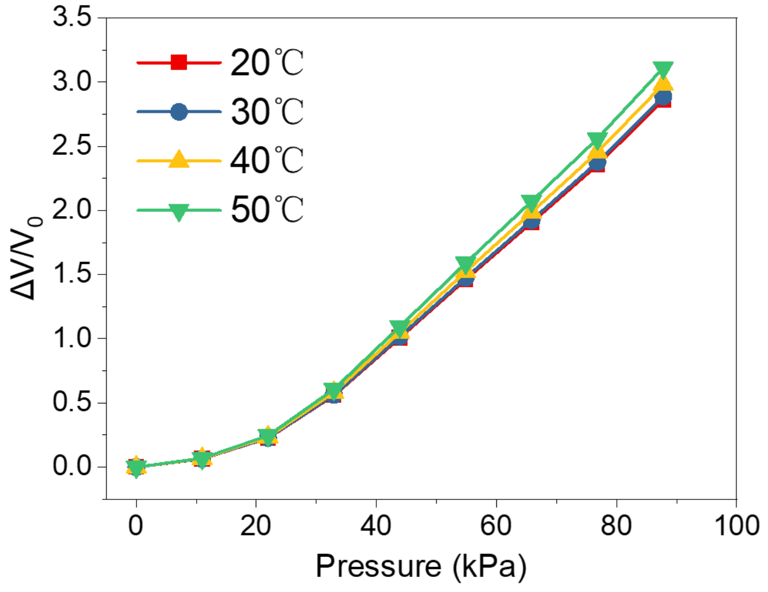

Supplementary Fig. 3. Temperature effect of liquid metal-based pressure sensor under varied loadings.

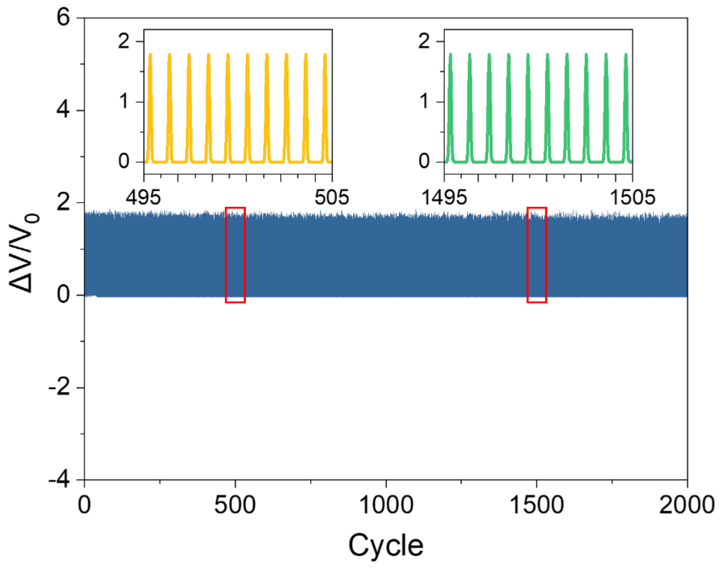

Supplementary Fig. 4. Stability test of liquid metal-based pressure sensor under cyclic loading.

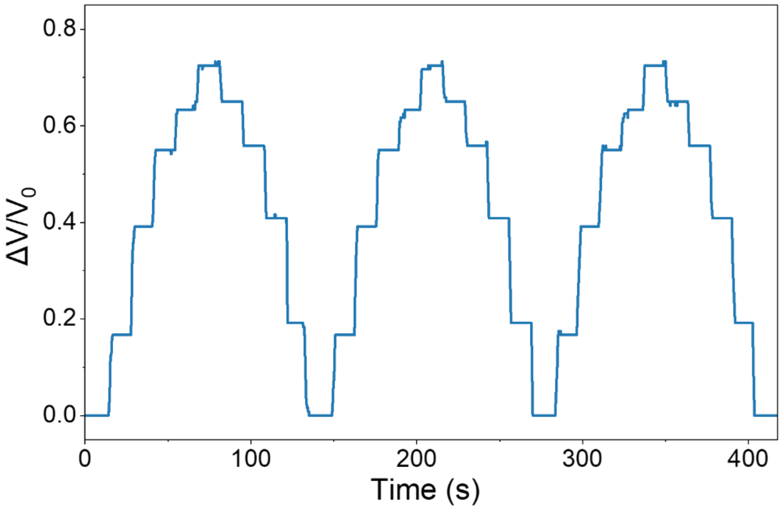

Supplementary Fig. 5. Repeatability test of pressure sensor under varied loading forces for three times.

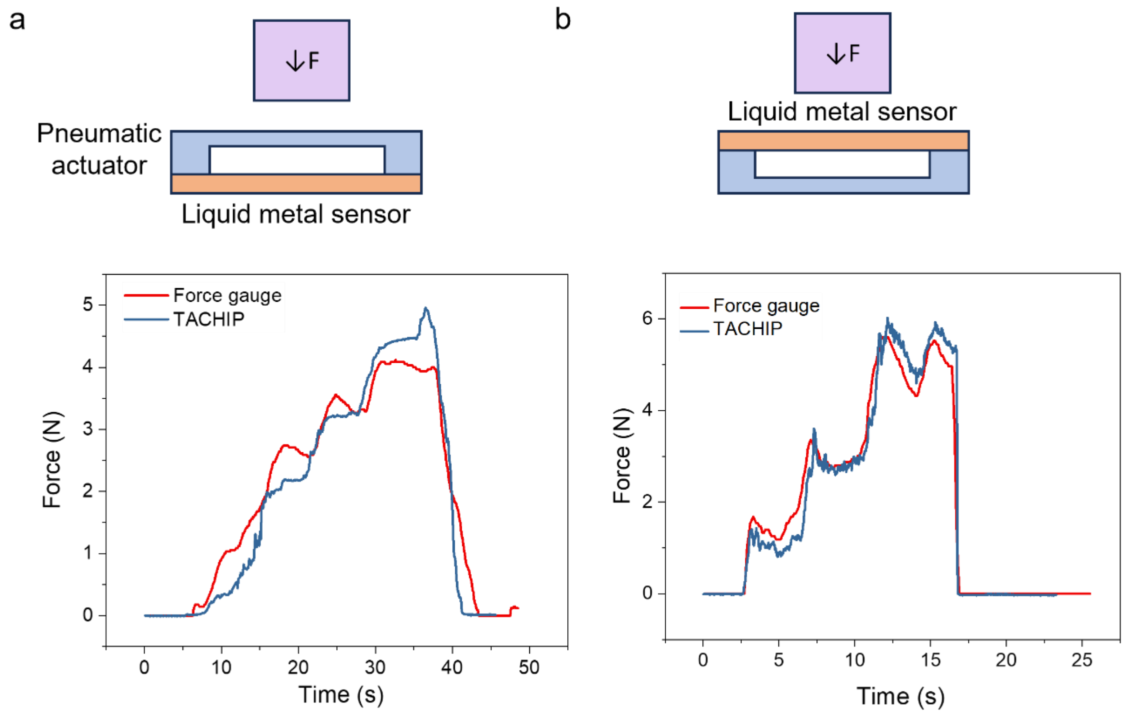

Supplementary Fig. 6. Comparison of the real applied forces (measured by force gauge) and the forces monitored by the in-situ pressure sensor via contact at **a** pneumatic actuator side and **b** pressure sensor side.

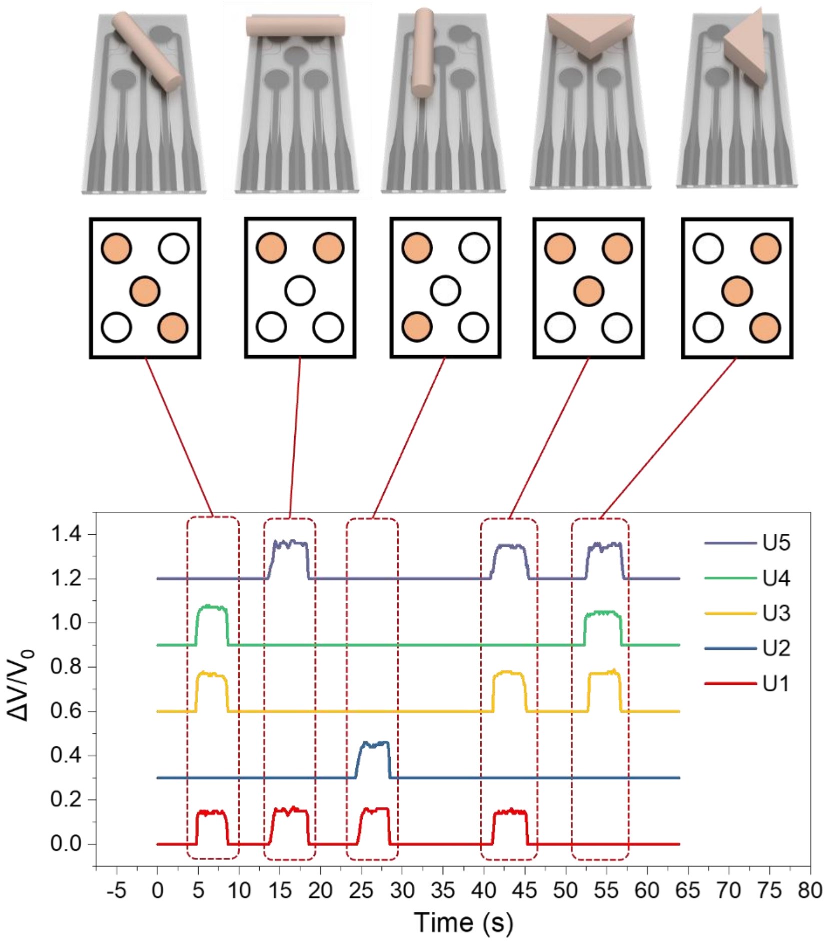

Supplementary Fig. 7. Pressure distribution sensing signals for different contacts with uniform loading.

Supplementary Note 3. Object recognition realized by distributive TACHIPs in soft robotic gripper.

Together with multi-finger soft robotic gripper, modular design of TACHIP is also capable of enabling intelligent object recognition via tactile information during grabbing. Supplementary Fig. 8 shows a typical three fingers soft gripper which were equipped with three TACHIPs. Three FPC connectors allows quick plug-in for convenient integration. Eleven objects, including sphere, cylinder, frustum, cube with four orientations, trapezoid with four orientations, were selected for collecting the training dataset. In Supplementary Fig. 8a, the training signals from all fifteen sensors are provided, and the key features within the signal dataset of each object can be easily observed. According to confusion matrix in Supplementary Fig. 8b, the average recognition accuracy can reach up to 98.68%, showing capability of identifying similar object or same object with different orientations.

For other irregular shapes, we can increase the number of gripper's fingers or completely replace the gripper by other soft manipulator with better surface coverage. Thus, the scalability of TACHIPs can be utilized for distributing across the soft manipulator. In this case, the natural shapes with more irregularities can be sensed thoroughly by new structure, which eventually improves the recognition accuracy.

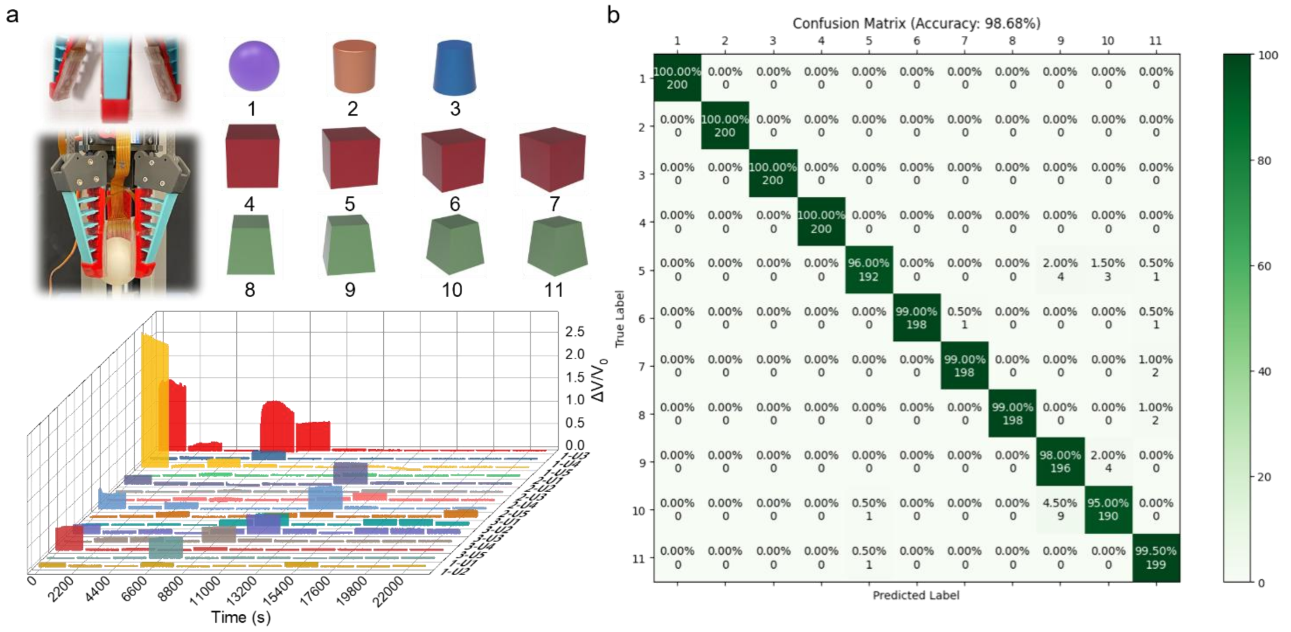

Supplementary Fig. 8. Recognition of grabbed object by attaching three TACHIPs on three finger fin ray robotic gripper, **a** photos of test setup, training object with different shapes and orientations, and training dataset of all 11 objects, **b** confusion matrix of recognition accuracy for TACHIP enabled intelligent gripper.

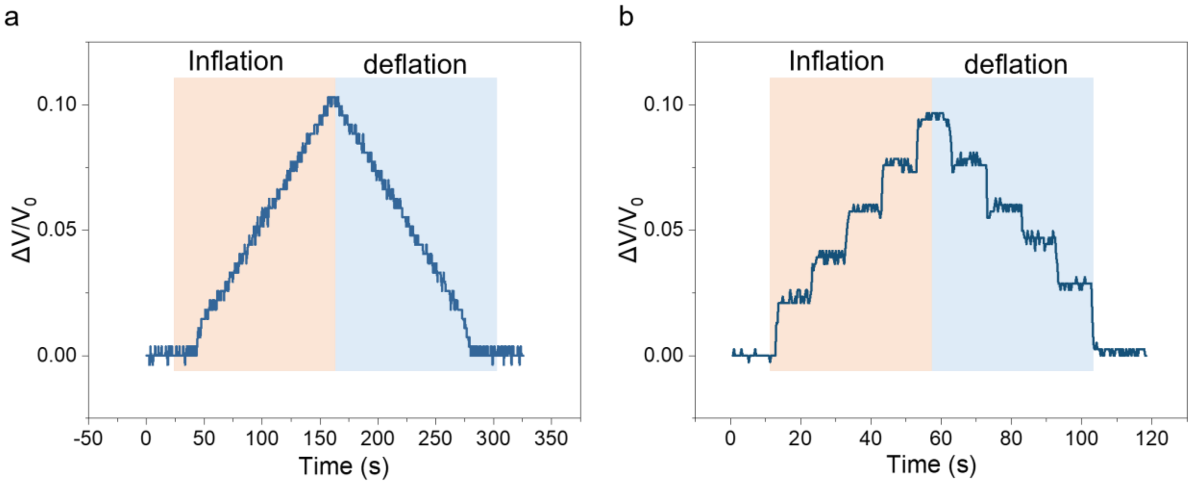

Supplementary Fig. 9. In-situ air pressure monitoring data via pressure sensor during pneumatic actuation for two different inflation and deflation processes, **a** continuous inflation and deflation process, and **b** stepwise inflation and deflation process.

Supplementary Note 4. Characterization data of pneumatic actuator in TACHIP

In Supplementary Fig. 10, silicone materials suitable for molding pneumatic actuator are studied. Specifically, four popular series, including Eco-flex 00-10, Eco-flex 00-30, Eco-flex 00-50, and

Dragon skin 30 (Smooth-on), were selected for conducting pneumatic actuation tests under a fixed gauge pressure of 20 Kpa. As can be seen in the test results, the material with lower Young's modulus offers larger haptic feedback force under a given air pressure, as the material itself possess less resistive force against deformation. On the other hand, it is also needed to limit the deformation rate of the as-fabricated pneumatic actuator to ensure the controllable actuation, especially for fine tuning application via parallel manipulator shown in Fig. 7. In the meantime, material fatigue was observed in Eco-flex 00-10 based pneumatic actuator after cyclic running under large air pressure. Thus, Eco-flex 00-30 was selected as the material used for fabricating pneumatic actuator.
Supplementary Fig. 11 shows pneumatic feedback with changing frequencies. As can be seen from the sensing data, as the actuation frequency increases for a given input pressure, the residual pressure is observed at the deflation stage, and higher frequency will cause larger residual pressure due to the time required for air flowing.

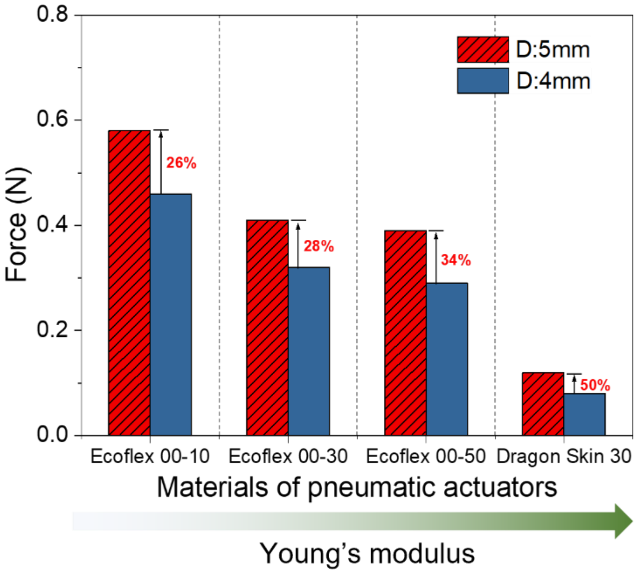

Supplementary Fig. 10. Comparison of pneumatic feedback forces from pneumatic actuators with different materials and sizes.

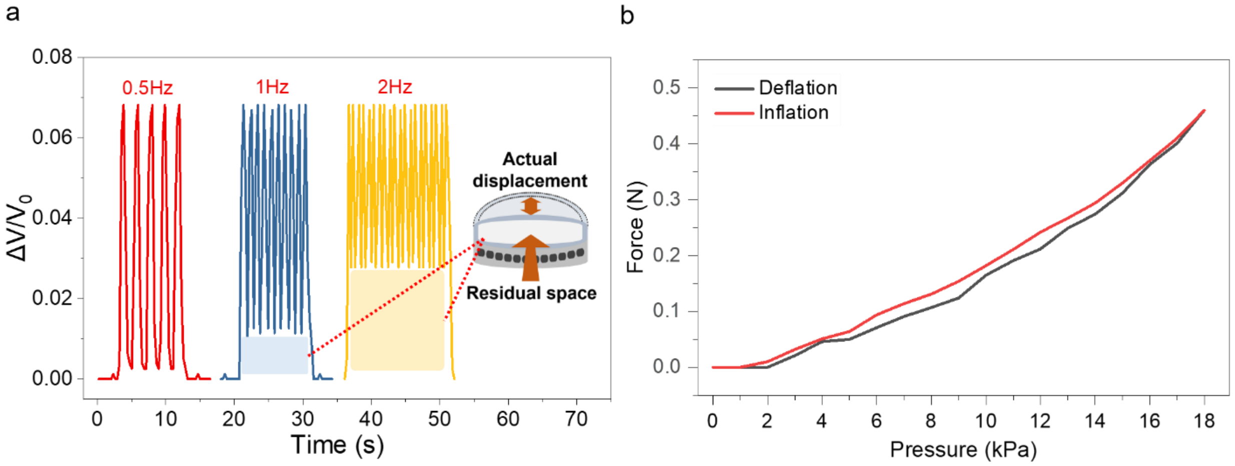

Supplementary Fig. 11. a In-situ sensing data for monitoring low frequency pneumatic actuation. **b** Hysteresis test of pneumatic actuation.

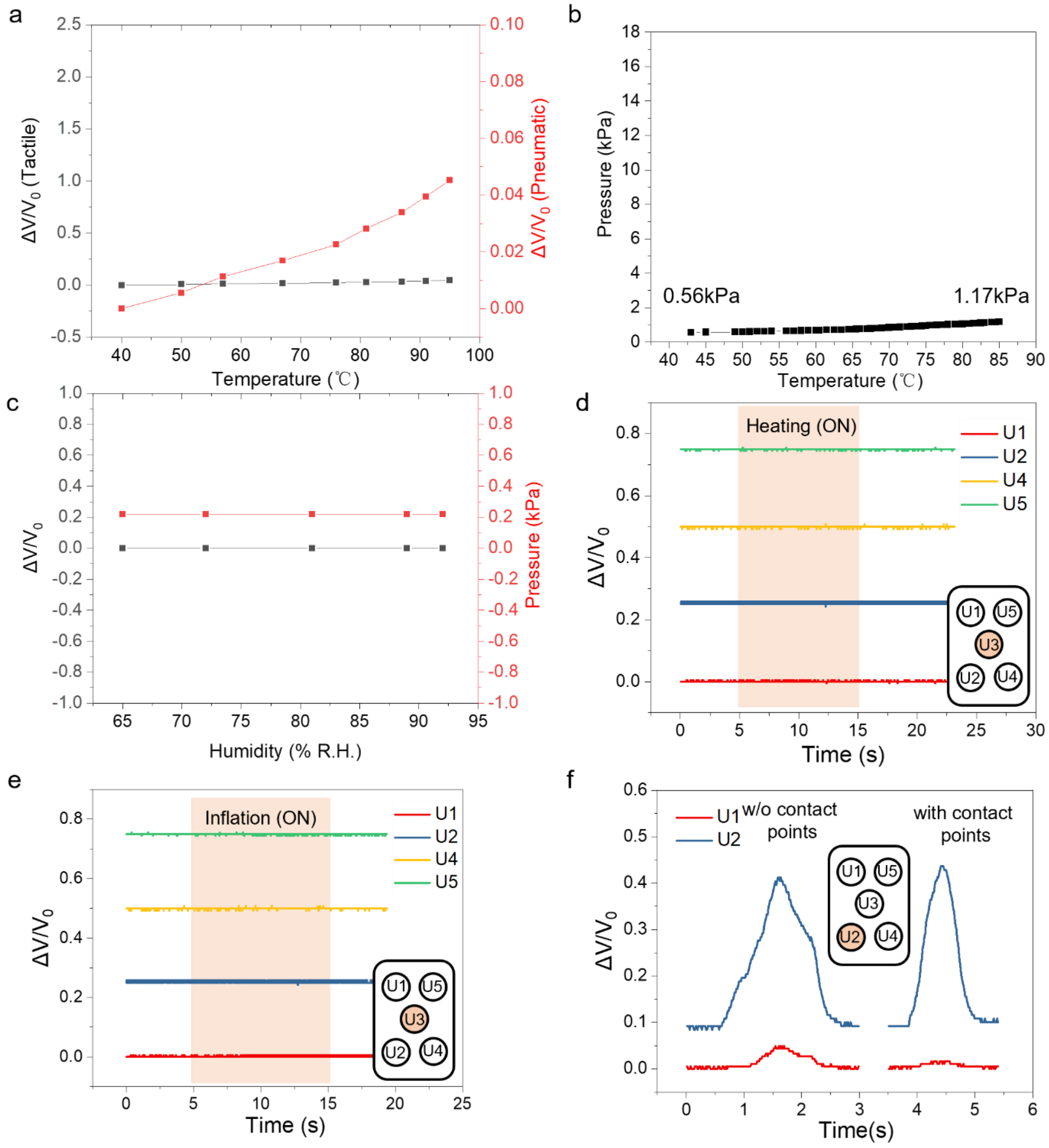

Supplementary Fig. 12. Influences of environmental variations and crosstalk to the in-situ pressure sensor and the pneumatic actuator. **a** Influences of temperature variations to the pressure sensing for tactile information and pneumatic monitoring applications. **b** Influences of temperature variations to the internal air pressure of the pneumatic actuator, with a pre-supplied pressure of 0.56kPa. **c** Influences of humidity on the in-situ pressure sensing and the internal pressure. d Evaluation of electrical crosstalk during thermal feedback at U3. e Evaluation of electrical crosstalk during pneumatic feedback at U3. f. Evaluation and optimization of interconnector deformation induced crosstalk under the applied force.

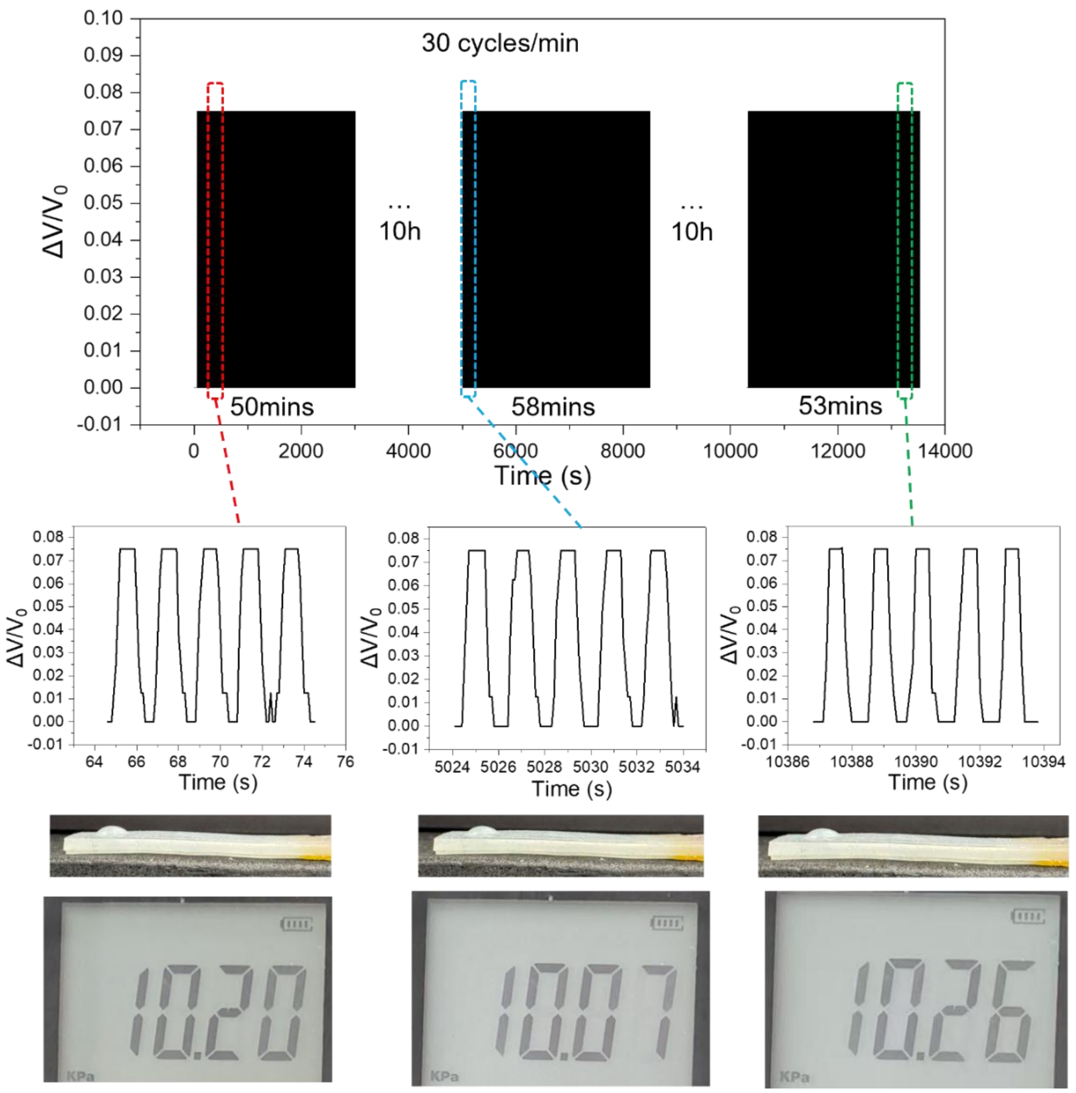

Supplementary Fig. 13. Reliability test for both in-situ pressure sensing and pneumatic actuator under cyclic operations of 24 hours (30 cycles/min). The photos of pneumatic actuation and the corresponding internal pressure are given.

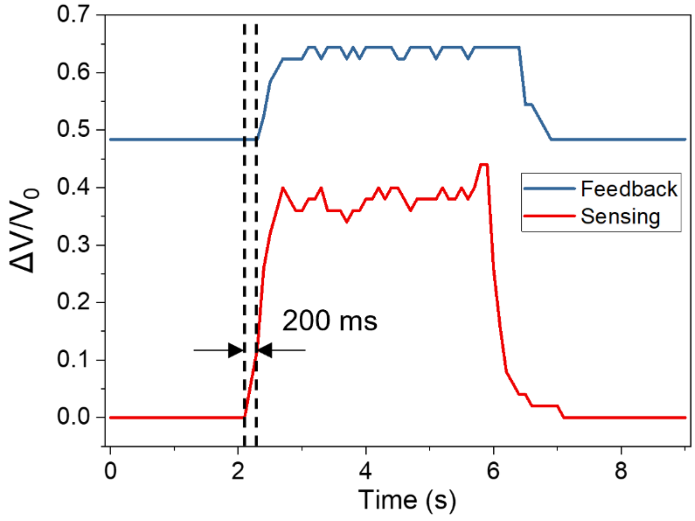

Supplementary Fig. 14. System latency between the sensing of tactile information and the feedback of the corresponding tactile information.

Supplementary Note 5. Teleoperation with dynamic sensing and haptic feedback functions.
During teleoperation, static and dynamic sensing and feedback of multi-modal information affects the on-site tactile awareness regarding the processed operations. The immediate adjustment based on haptic feedback can significantly increase the success rate of specific tasks. In Supplementary Fig. 15, systematic demonstration of fusion of multi-modal sensing and feedback with TACHIPs are illustrated. Additionally, dynamic events are frequently observed when the grabbing force or position are inappropriate, and eventually cause sliding or rolling of the target object. In Fig. 6b, the experiment of projecting the sliding/rolling event from follower side with spatiotemporal information into leader side via TACHIPs was conducted. The left side of Supplementary Fig. 15a shows the spatiotemporal data of contact pressure sensing from TACHIP of follower side, and the recorded data curves from five sensors (Supplementary Fig. 15b). Once the pressure data exceeds the threshold, the corresponding pneumatic actuator would be inflated with the input air pressure controlled by the predefined relationship against the contact pressure. The right side of Supplementary Fig. 15a shows the spatiotemporal data of pneumatic haptic feedback from TACHIP of leader side, and the recorded data curves from five in-situ sensors (Supplementary Fig. 15b) during actuation. According to the data from both sides, the relative good consistency of spatiotemporal tactile information detected from follower side and regenerated at leader side shows the feasibility of using TACHIPs for building dual way tactile communication. Noticeably, as the pressure monitoring data from leader side contains both pneumatic pressure and finger pressing, there are still some mismatches compared to the pressure monitoring data from follower side. Other possible reasons include the non-uniform membranes of five pneumatic actuators during fabrication, residual air pressure, and inconsistency of sensitivity among five sensors for small pressure.

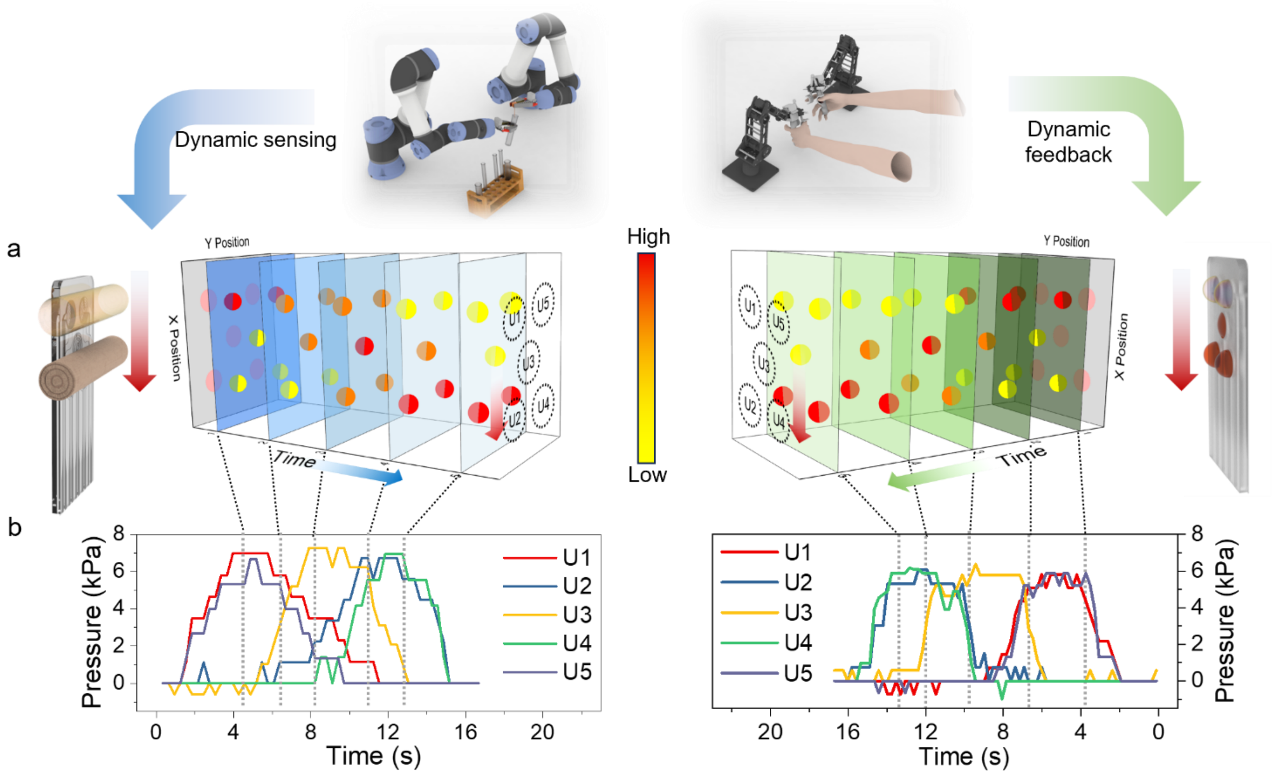

Supplementary Fig. 15. Schematics of teleoperation with dynamic sensing and haptic feedback functions. **a** Spatiotemporal tactile information between follower side (left) and leader side (right) during the sensing of the moving object and the real-time haptic feedback of the motion, the legends of the color indicate the measured pressure. **b** Continuous pressure data of sensor array for monitoring dynamic motion and pneumatic actuations during haptic feedback.

Supplementary Note 6. Design of subjective experiment for haptic feedback

Subjective experiments for haptic feedback patterns are labelled as Pattern 1 (P1), Pattern 2 (P2)…, etc. Ten volunteers are involved in this blind test. For each volunteer, each pattern was activated three times in total, but will be randomly arranged together with other patterns. For example of two patterns test, participant A will be tested as: P1→P2→P1→P1→P2→P2, participant B will be tested as P2→P2→P1→P1→P1→P2, etc. Overall, a single pattern would be tested 30 times during the entire experiment. Therefore, a specific test program is designed for each participant. To ensure the quality and duration of generated patterns are sufficient for participants to perceive, each pattern is set to active for 5 seconds, and followed by 10 seconds of gap before the next pattern. During the gap time, the participants will mark down the identified pattern of the previous round on questionnaire. Finally, the statistical data will be obtained and evaluated based on patterns and participants.

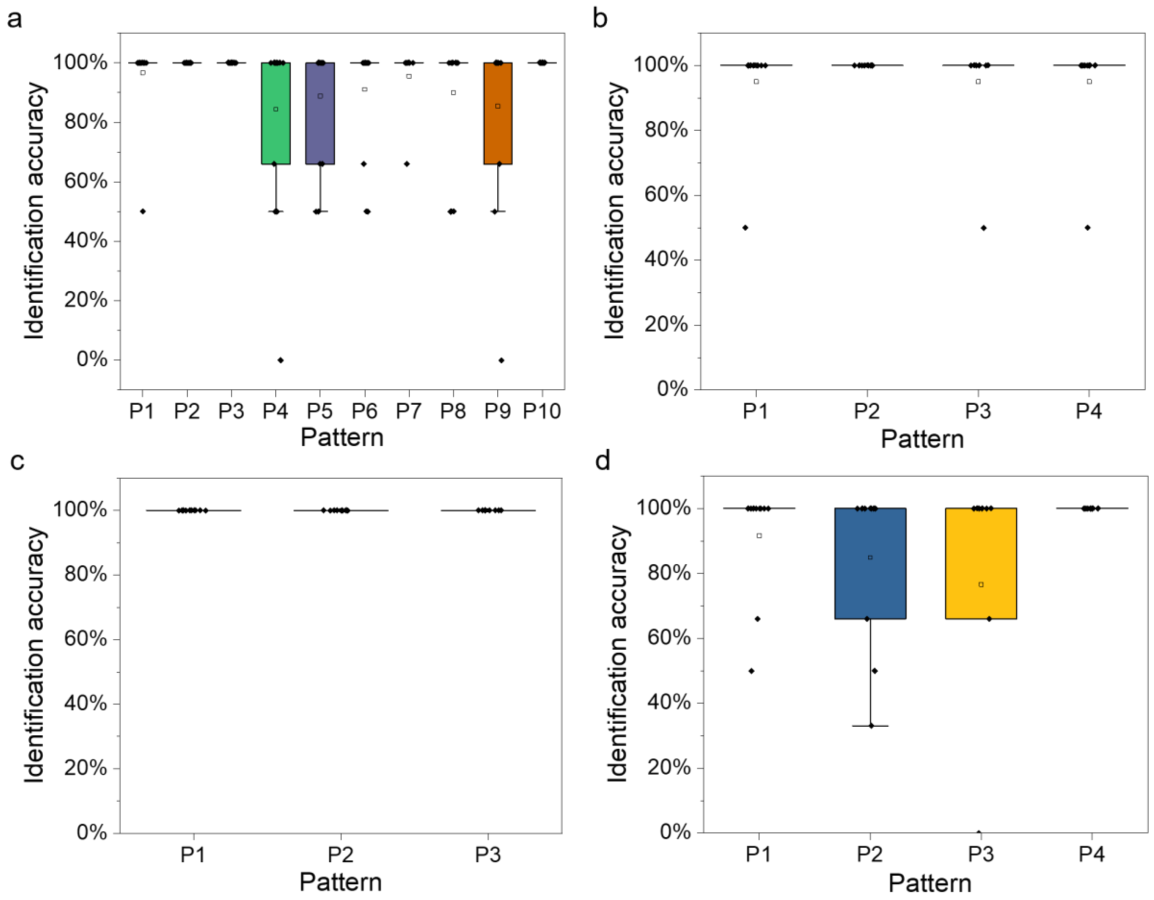

Supplementary Fig. 16. Identification accuracy of subjective experiment of **a** static and dynamic patterns, **b** patterns with four different geometries, **c** patterns with different hardness, **d** patterns with different frequencies. Boxes show median and interquartile range, whiskers show 1.5 times of interquartile range, points are the individual values.

The data analysis on the basis of participants investigates the individual differences in perceiving similar haptic feedback (Supplementary Fig. 16). It is essential to obtain guidelines for understanding the general standard of designing actuation program. On the other hand, the data analysis based on patterns facilitates the study about the similarities and uniqueness of the regenerated perception among various patterns. It helps to modify the actuation parameters, such as sequence, delay time, input pressure, etc., to reach a relative distinct feeling to improve the identification accuracy.

Noticeably, with the aid of in-situ pressure sensor, all of actual performances of those haptic feedback patterns can be digitally recorded, instead pure statistical data from subjective experiments. Hence, these sensing data can be directly treated as training dataset for machine learning assisted evaluation and modification program for haptic feedback actuation coding. As a complementary technique, it can greatly reduce the manpower cost in evaluating the haptic feedback performance via subjective experiment, and facilitate the research of actuation logic and strategy for more realistic perception regeneration using specific haptic feedback mechanisms.

Exploratory Procedures is purposive action pattern that perceivers execute to encode properties of surfaces and objects, and mainly consists of six procedures: (1) lateral motions for surface texture, (2) pressure for compliance or hardness, (3) static contact for apparent temperature, (4) unsupported

holding for weight, (5) enclosure for volume and shape, (6) contour following for exact shape. This concept indicates the importance of spatiotemporal information in exploring objects or dynamic events. In terms of spatiotemporal information, it mainly consists of time and position/displacement/shape. In this work, time information is represented by inflation time (tx), gap time (Δtx-y), and actuation frequency (fx). Position/displacement/shape information is represented by actuation pressure (Px) and differential pressure (Px-y).

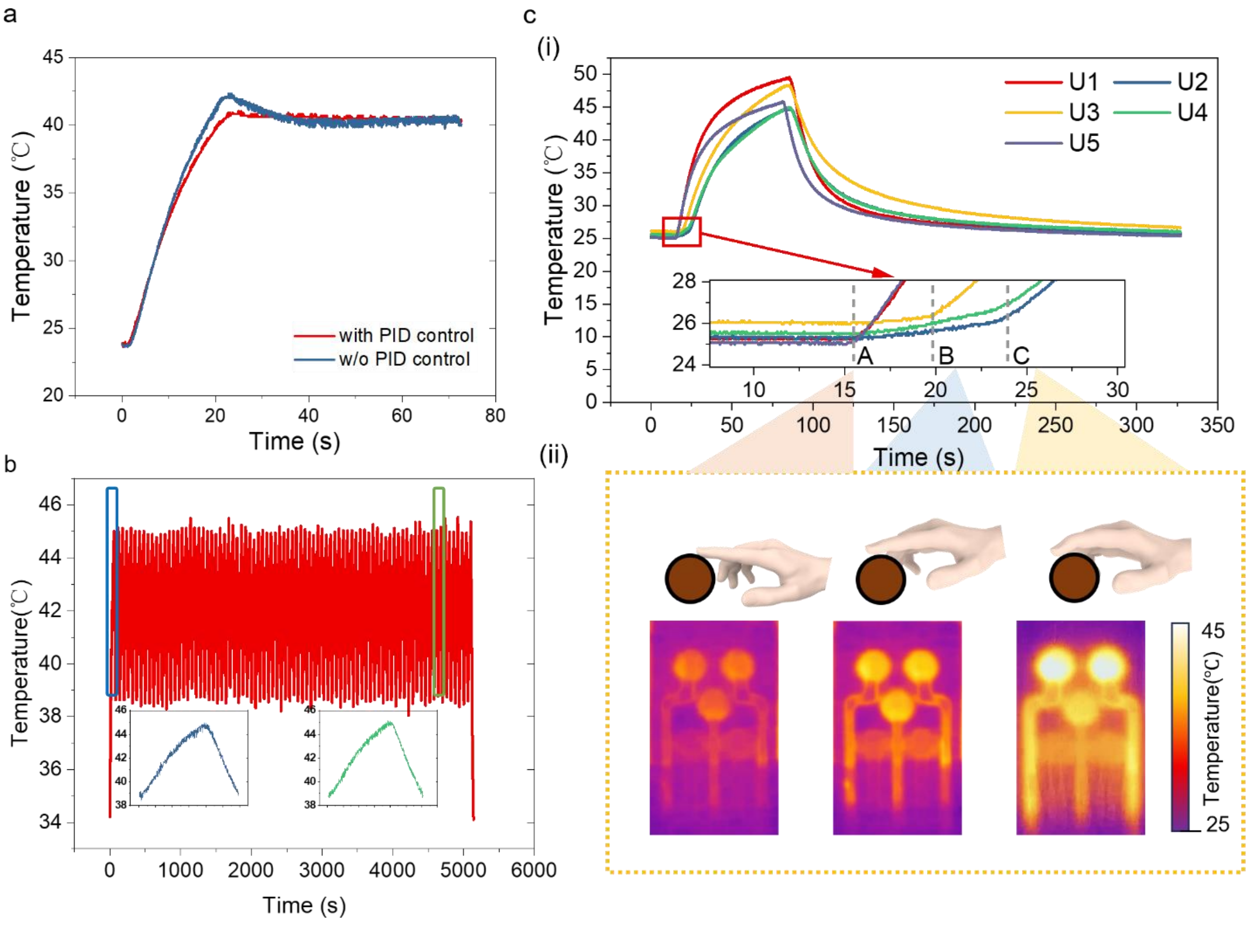

Supplementary Fig. 17. a Comparison of thermal feedback with and without PID control. **b** Cyclic test of thermal feedback for a target temperature of 45 °C. **c** Experimental data of dynamic thermal feedback for regenerating thermal perception during sequential contact, the inset shows the temporal information during contact process, with the schematics and infra-red images of three stages of thermal feedback.

Supplementary Note 7. Approximation of temperature distribution via sensing fusion

Thermal feedback function is realized by applying temperature sensor and liquid metal-based heater. However, as the concept of minimalistic design, there is only one temperature sensor integrated in TACHIP. To achieve regeneration of temperature distribution with five heaters, the sensing fusion approach with temperature approximation method is adopted (Supplementary Fig. 18). The basic idea of this proposed method is to build a relationship between the contact pressure level and the thermal conduction-based temperature level.

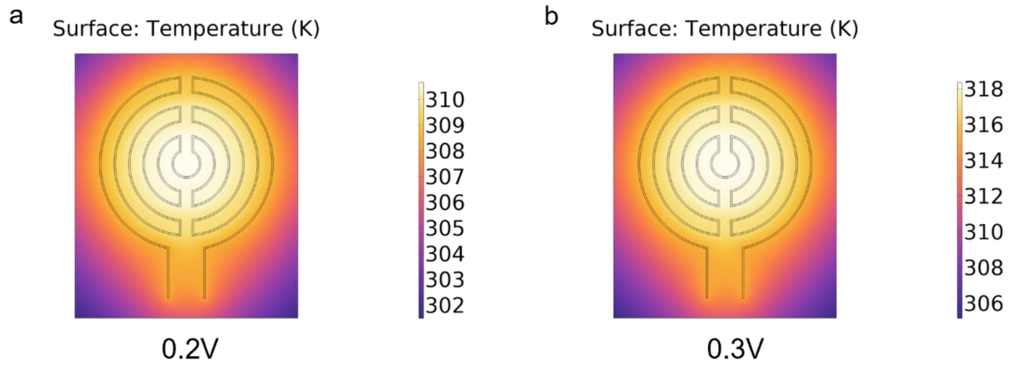

Supplementary Fig. 18. Finite element analysis of liquid metal-based heater via supplied voltage of **a** 0.2V and **b** 0.3V.

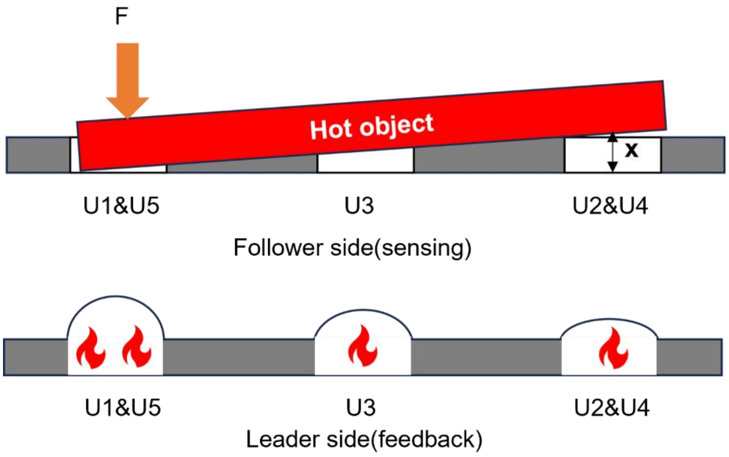

Supplementary Fig. 19. Schematics of approximation of temperature distribution for thermal sensing and feedback.

The temperature sensor is located beside pressure sensors of U2 and U4. Pneumatic chambers above pressure sensors offer deformable gaps during contact. When TACHIP starts contacting with an object, the amplitudes of the pressure signals indicate the spacing between the sensor and the object. For hot object, after knowing the temperature data from the reference sensor (pectin thin film temperature sensor beside U2 and U4), the temperature distribution perceived by TACHIP can be approximated by considering thermal conductivity of air and the specific spacing. Based on Fourier's law:

$$q = -\lambda \frac{dT}{dx} \quad (1)$$

With integration of this equation, and set the temperature at position $x=x_1$ and x_2 to be T_1 and T_2, respectively, and assume heat flux q is constant. The following equation can be obtained for calculating temperatures at the corresponding positions:

$$T_2 = T_1 - \frac{q}{\lambda}(x_2 - x_1) \quad (2)$$

Where x is proportional to pressure sensing data. It is worth noting that this approximation of temperature distribution is not accurate about the real temperature at each point. There are several potential issues that will cause huge deviations between approximated and real temperature, such as shape of object, materials variations within the object, convection, etc. The main purpose of adopting

this approach is to provide a basic perception regarding the dynamic changes of temperature distribution during interaction. Considering thermal radiation and conduction, user may not be able sense the temperature distribution with high resolution and sensitivity. Therefore, it may not be necessary to integrate a dense array of temperature sensors and heaters within small area, such as TACHIP. The fusion of pressure sensors and one temperature sensor is still a feasible way to provide general thermal information.

Supplementary Note 8. Kinematic analysis of TACHIP enabled parallel manipulator

As shown in Fig. 7a(ii), the length parameters of l_{u1}, l_{u2}, l_{u3}, l_{u4}, l_{l1}, l_{l2}, l_{l3}, and l_{l4} represent pneumatic deformation heights of four actuators at upper and lower TACHIPs, respectively, and b is the initial deformation height of all eight pneumatic actuators. The parameter of 2a is the spacing between two adjacent working pneumatic actuators. Two center pneumatic actuators act as support pivots for upper and lower surfaces. The tip coordinates of the grabbed tool is set as $P(x_p, y_p, z_p)$. The kinematics of this 3-RPS/US (revolute-prismatic-spherical joint/universal-spherical joint) parallel manipulator is given as follows.

The coordinates of platforms are presented as matrix M,

$$M = \begin{bmatrix} x_1 & x_2 & x_3 & x_4 \\ y_1 & y_2 & y_3 & y_4 \\ z_1 & z_2 & z_3 & z_4 \end{bmatrix} \quad (1)$$

and the corresponding platforms are marked as lower static platform (M_{sl}), lower motional platform (M_{ml}), upper static platform (M_{su}), and upper motional platform (M_{mu}).

$$M_{sl} = \begin{bmatrix} a & -a & -a & a \\ a & a & -a & -a \\ 0 & 0 & 0 & 0 \end{bmatrix} \quad (2)$$

$$M_{ml} = \begin{bmatrix} a & -a & -a & a \\ a & a & -a & -a \\ b & b & b & b \end{bmatrix} \quad (3)$$

$$M_{mu} = \begin{bmatrix} a & -a & -a & a \\ a & a & -a & -a \\ H & H & H & H \end{bmatrix} \quad (4)$$

$$M_{su} = \begin{bmatrix} a & -a & -a & a \\ a & a & -a & -a \\ 2a+b & 2a+b & 2a+b & 2a+b \end{bmatrix} \quad (5)$$

For any tip coordinates of the grabbed tool: $P(x_p, y_p, z_p)$, let:

$$\theta_z = \arctan\frac{y_P}{x_P} \quad (6)$$

$$\theta_y = \arctan\frac{z_P-(a+b)}{x_P} \quad (7)$$

The final position of the lower motional platform is:

$$M'_{ml} = M_{ry}(\theta_y) \cdot M_{rz}(\theta_z) \cdot \left(M_{ml} - \begin{bmatrix} 0 & 0 & 0 & 0 \\ 0 & 0 & 0 & 0 \\ a+b & a+b & a+b & a+b \end{bmatrix} \right) + \begin{bmatrix} 0 & 0 & 0 & 0 \\ 0 & 0 & 0 & 0 \\ a+b & a+b & a+b & a+b \end{bmatrix} \quad (8)$$

The final position of the upper motional platform is:

$$M'_{mu} = M_{ry}(\theta_y) \cdot M_{rz}(\theta_z) \cdot \left(M_{mu} - \begin{bmatrix} 0 & 0 & 0 & 0 \\ 0 & 0 & 0 & 0 \\ a+b & a+b & a+b & a+b \end{bmatrix} \right) + \begin{bmatrix} 0 & 0 & 0 & 0 \\ 0 & 0 & 0 & 0 \\ a+b & a+b & a+b & a+b \end{bmatrix} \quad (9)$$

Among them:

$$M_{rz}(\theta) = \begin{bmatrix} \cos\theta & -\sin\theta & 0 \\ \sin\theta & \cos\theta & 0 \\ 0 & 0 & 1 \end{bmatrix} \quad (10)$$

$$M_{ry}(\theta) = \begin{bmatrix} \cos\theta & 0 & -\sin\theta \\ 0 & 1 & 0 \\ \sin\theta & 0 & -\cos\theta \end{bmatrix} \quad (11)$$

The following equations are set:

$$M'_{ml} - M_{sl} = (\overrightarrow{\alpha_1}, \overrightarrow{\alpha_2}, \overrightarrow{\alpha_3}, \overrightarrow{\alpha_4}) \quad (12)$$

$$M'_{mu} - M_{su} = (\overrightarrow{\beta_1}, \overrightarrow{\beta_2}, \overrightarrow{\beta_3}, \overrightarrow{\beta_4}) \quad (13)$$

The inverse kinematics solutions for the pneumatic deformation heights are set as follows:

$$l_{li} = |\overrightarrow{\alpha_i}| \qquad \text{i=1,2,3,4} \quad (13)$$

$$l_{ui} = |\overrightarrow{\beta_i}| \qquad \text{i=1,2,3,4} \quad (14)$$

To solve the non-linear equations, Newton downhill method is used for direct kinematic solutions:

$$X^{k+1} = X^k - w\left(J(X^k)\right)^{-1} f(X^k) \qquad (0<\text{w}\leqslant 1) \quad (15)$$

Where w is the downhill factor with the termination condition when:

$$\|\Delta X^k\| < \Gamma \quad (16)$$

And

$$\|f(X^{k+1})\| < \|f(X^k)\| \quad (17)$$

Where Γ is the smallest integer, which is greater than zero, $J(X^k)$ is the Jacobian matrix obtained by derivation of functions for the manipulator.

$$J = \begin{bmatrix} \frac{\partial F_1}{\partial \theta_1} & \frac{\partial F_1}{\partial \theta_2} \\ \frac{\partial F_2}{\partial \theta_1} & \frac{\partial F_2}{\partial \theta_2} \end{bmatrix} \quad (18)$$

By using MATLAB for solving above kinematics solution, the workspace and the operation trajectories of tip point of the grabbed tool can be obtained with the deformation parameters of each pneumatic actuator, as shown in Fig. 7c. According to Fig. 7c(ii), with the empirical equation defining

the relationship between the deformation parameter and the input air pressure, the control program of pneumatic arrays on TACHIPs can be developed to manipulate the tool toward desired position. Detailed displacements of the corresponding actuator array can be seen in Supplementary Fig. 20.

Supplementary Table 1. Definitions of parameters shown in kinematic analysis

Parameter	Definition
M_{sl}	Matrix expression of the lower fixed platform
M_{ml}	Matrix expression of the lower moving platform
M_{mu}	Matrix expression of the upper moving platform
M_{su}	Matrix expression of the upper fixed platform
θ_z	Rotation angle of the rod along the z-axis of the coordinate system
θ_y	Rotation angle of the rod along the y-axis of the coordinate system
x_P	x-coordinate of the end point (tip)
y_P	y-coordinate of the end point (tip)
z_P	z-coordinate of the end point (tip)
a	Half-length of two adjacent working pneumatic actuators
b	Initial height of working pneumatic actuator at grabbing stage
H	Distance between the two platforms formed by two TACHIPs
M'_{ml}	Matrix expression of the final position of the lower moving platform
M'_{mu}	Matrix expression of the final position of the upper moving platform
M_{rz}	Rotation transformation matrix about the z-axis of the coordinate system
M_{ry}	Rotation transformation matrix about the y-axis of the coordinate system
l_{li}	Final height of the lower pneumatic actuator
l_{ui}	Final length of the upper pneumatic actuator
α_i	Pose vector expression of the lower pneumatic actuator
β_i	Pose vector expression of the upper pneumatic actuator
X^k	Approximate solution obtained at the k-th iteration in the Newton downhill method
w	Downhill factor in the Newton downhill method
Γ	Smallest positive integer greater than zero in the Newton downhill method
f	Objective function in the Newton downhill method
J	Jacobian matrix of the objective function in the Newton downhill method

It is worth noting that the above calculation does not consider the variation of the external loading forces. The actual displacement may vary as the external loading changes. The main issue of large deviation from the desired position may refer to the deformation caused by the contact force when the object is manipulated. There will be unpredictable external loading forces applied on the pneumatic actuator for operating random objects. The additional loading force will firstly reduce the displacement calculated by simulation with little or no external loadings. and also change the input pressure required

to twist the tip to a desired position. Specifically, as the mass of the object increases, larger input pressures/stiffness are required for moving the same distance.

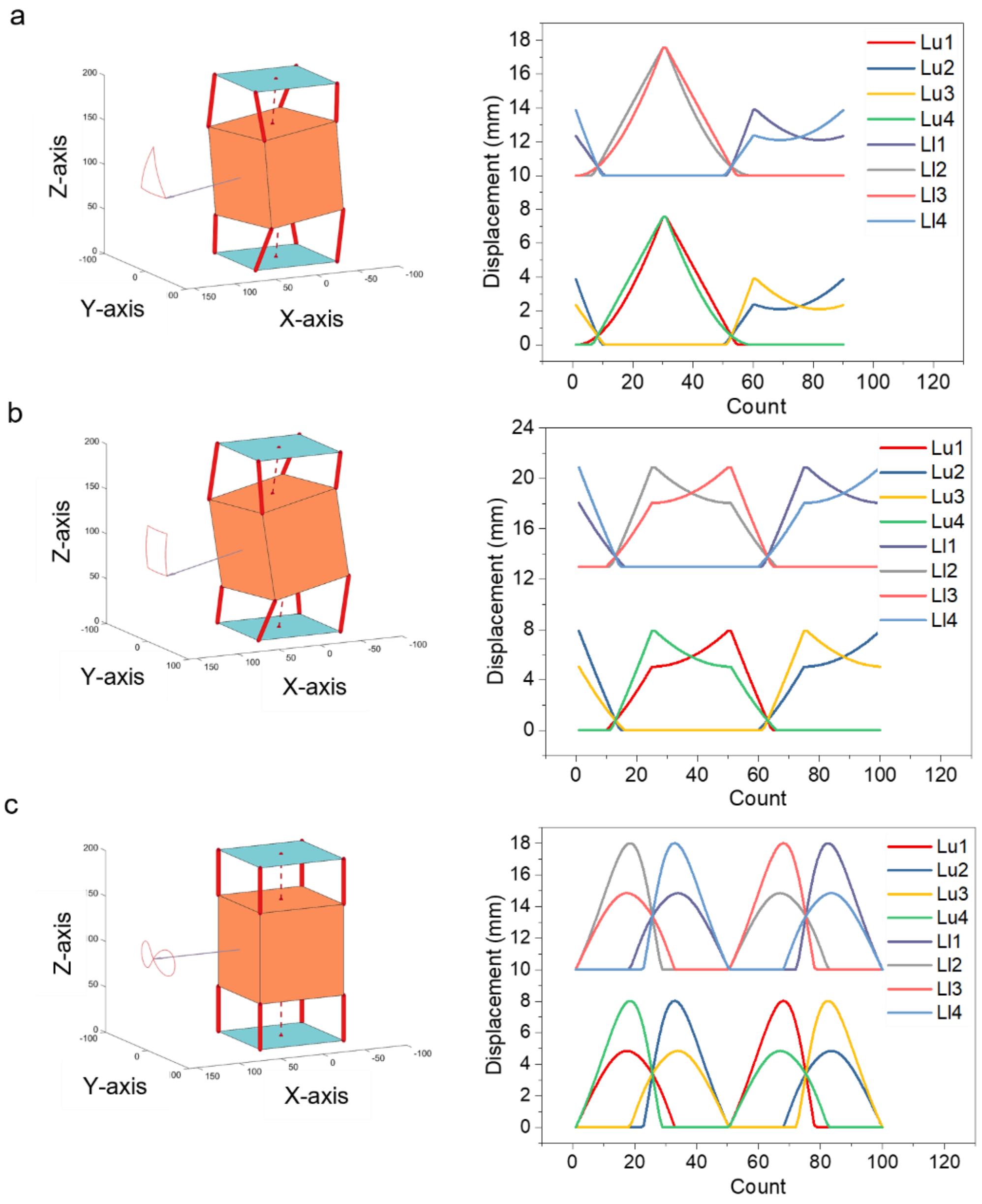

Supplementary Fig. 20. Displacements of the corresponding pneumatic actuators for completing the trajectories of **a** Triangle, **b** Rectangle, **c** Infinite sign.

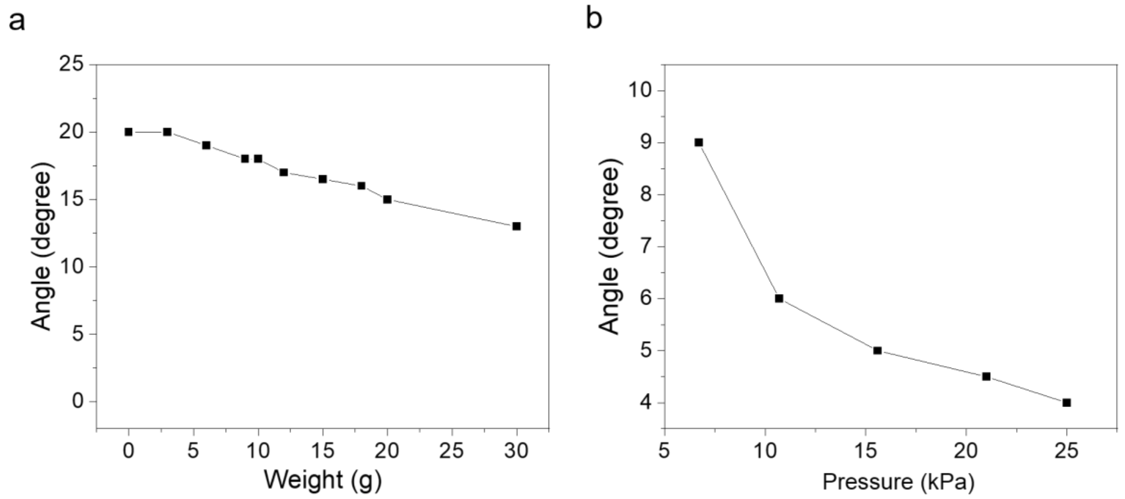

Supplementary Fig. 21. a Angle variations of the tip of the tool under the increased loading weight at the tip. **b** Deflection angle of the tip of the tool under the changing input pressure (stiffness) of the actuator for a given load (30g).

Supplementary Note 9. Teleoperation system used for demonstration

The open-source project "ALOHA: A Low-cost Open-source Hardware System for Bimanual Teleoperation" developed by researchers from Stanford University was utilized as reference to build the hardware platform for collaborative teleoperation system. As shown in Supplementary Fig. 22, the main components of this system include two follower robots (Widow 250 Robot Arm 6DOF), two leader robots (ViperX 300 Robot Arm 6DOF), and a computer installed with Ubuntu 20.04 + ROS 1 noetic operation system.

As shown in Supplementary Fig. 22, the gripper side of leader and follower robots were customized with 3D printed accessories. Fin ray effect soft grippers with TACHIPs were installed at follower side. This gripper possesses better conformability when grabbing object with different shapes, while it does not require the replacement of the original actuation motor. Control levers with TACHIPs were installed at leader side for users to perform grab and release action. Back-end processing modules were attached on arms of robots.

Sensing and feedback pipeline:

Gross teleoperation stage

1) Calibration of sensors
2) Detection of sensing range of operation (Follower side)
3) Determination of projection ratio between sensing and feedback (Leader side)
4) Real-time sensing of force and temperature and wireless transmission (Follower side)
5) Receive sensing signals and analog write (with determined projection ratio) of PWM ports for actuation of pneumatic pump and heater (Leader side)
6) In-situ monitoring of actuation status (Leader side)

Fine teleoperation stage

1) Switch of sensing and feedback program and lock the gripper
2) Mode 1: input of coordinates to kinematic model for calculating the actuation parameters/Mode 2: real-time sensing of force, wireless transmission of data (Leader side)

3) Receive direct actuation parameters or sensing signals and analog write (with determined projection ratio) of PWM ports for actuation of pneumatic pump (Follower side)

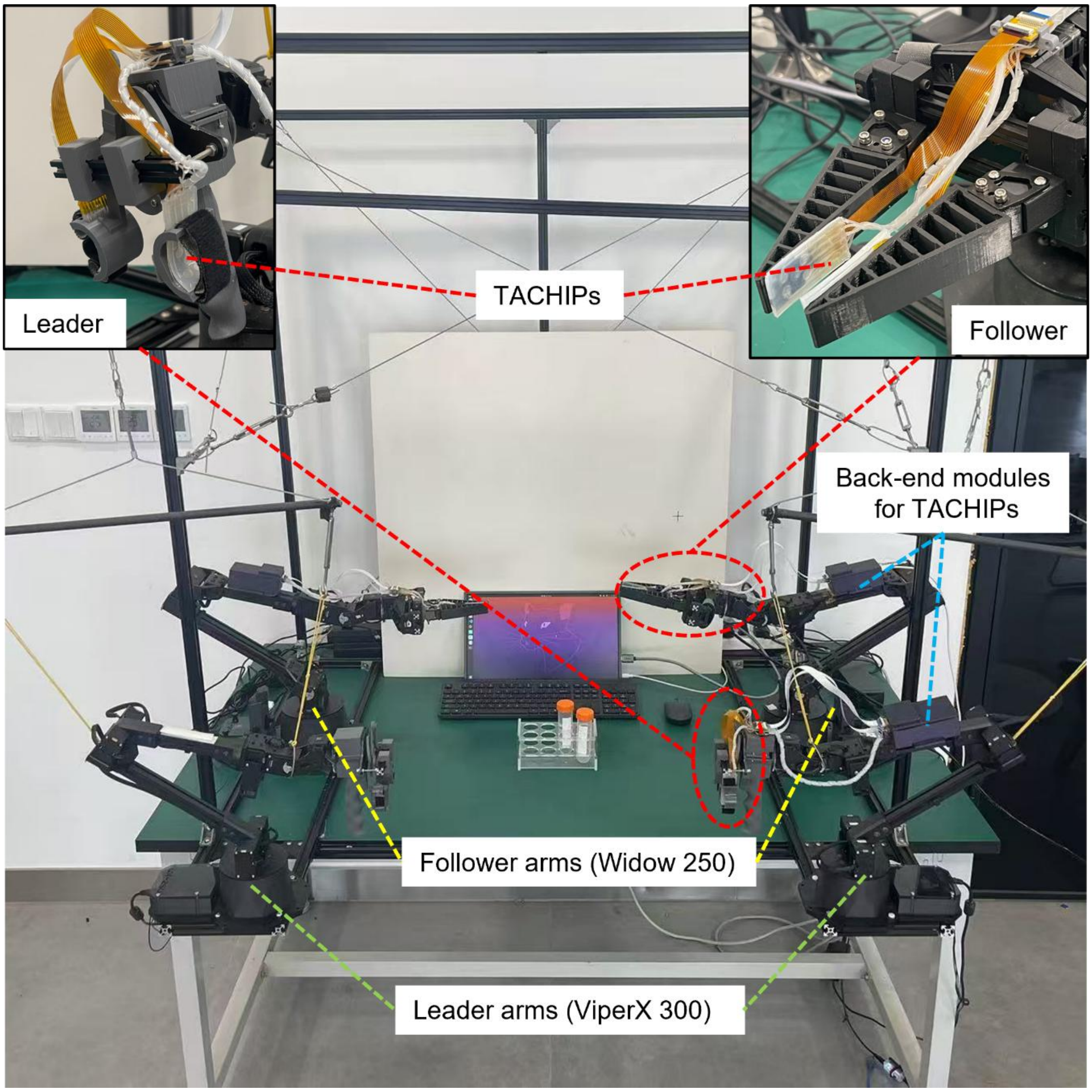

Supplementary Fig. 22. Photo of TACHIPs enhanced gross to fine teleoperation system.

Supplementary Table 2. Comparisons of functionalities and performances of haptic feedback interfaces

References	Maximum feedback force (mN)	Operation frequency (Hz)	Force sensitivity (N^{-1}) (sensing range, N)	Temperature sensitivity $°C^{-1}$(sensing range, °C)	Thermal feedback	In-situ sensing	Shape feedback	Shear force feedback	Manipulator	Complete flexible
[1]	3000	0-170	/	/	/	/	/	/	/	/
[2]	/	130-230	/	0.3577 (30-70)	Yes	Yes	/	/	/	/
[3]	1300	0-60	0.014 (1-20)	/	/	/	Yes	Partially	/	/
[4]	/	1-1000	0.014 (0.003-0.08)	/	/	/	Yes	Partially	/	/
[5]	100	0-320	/	/	/	/	Yes	Partially	/	/
[6]	1400	0-200	/	/	/	/	Yes	Yes	/	/
[7]	/	/	/	/	/	/	Yes	Partially	/	Yes
[8]	300	0-200	/	/	/	/	/	Yes	/	/
[9]	/	130-230	/	/	Yes	/	/	/	/	/
[10]	300	0-200	/	/	/	/	Yes	Partially	/	Yes
[11]	1000	0.2	/	/	/	/	Yes	/	/	/
[12]	135	100-300	/	/	/	/	Partially	Partially	/	/
[13]	370	/	/	/	/	/	Yes	/	/	/
[14]	2400	0-300	/	/	/	/	Yes	Partially	Yes	Yes
[15]	2200	0-500	2.55 (0-0.6)	/	/	Yes	Yes	Partially	/	/
This work	1670	0-1000	1.59 (0.2-1.76)	0.325	Yes	Yes	Yes	Partially	Yes	Yes

References:

1. Qi, J., Gao, F., Sun, G., Yeo, J. C. & Lim, C. T. HaptGlove—Untethered Pneumatic Glove for Multimode Haptic Feedback in Reality–Virtuality Continuum. *Adv. Sci.* **10**, 1–12 (2023).
2. Sun, Z., Zhu, M., Shan, X. & Lee, C. Augmented tactile-perception and haptic-feedback rings as human-machine interfaces aiming for immersive interactions. *Nat. Commun.* **13**, 5224 (2022).
3. Liu, M. *et al.* Tactile Sensing and Rendering Patch with Dynamic and Static Sensing and Haptic Feedback for Immersive Communication. *ACS Appl. Mater. Interfaces* **16**, 53207–53219 (2024).
4. Jin, H. *et al.* Highly pixelated, untethered tactile interfaces for an ultra-flexible on-skin telehaptic system. *npj Flex. Electron.* **6**, (2022).
5. Shen, V., Rae-Grant, T., Mullenbach, J., Harrison, C. & Shultz, C. Fluid Reality: High-Resolution, Untethered Haptic Gloves using Electroosmotic Pump Arrays. in *Proceedings of the 36th Annual ACM Symposium on User Interface Software and Technology* 1–20 (ACM, 2023). doi:10.1145/3586183.3606771.
6. Flavin, M. T. *et al.* Bioelastic state recovery for haptic sensory substitution. *Nature* **635**, 345–352 (2024).
7. Yao, K. *et al.* A fully integrated breathable haptic textile. *Sci. Adv.* **10**, eadq9575 (2024).
8. Ha, K.-H. *et al.* Full freedom-of-motion actuators as advanced haptic interfaces. *Science (80-.).* **387**, 1383–1390 (2025).
9. Oh, J. *et al.* A Liquid Metal Based Multimodal Sensor and Haptic Feedback Device for Thermal and Tactile Sensation Generation in Virtual Reality. *Adv. Funct. Mater.* **31**, 2007772 (2021).
10. Leroy, E., Hinchet, R. & Shea, H. Multimode Hydraulically Amplified Electrostatic Actuators for Wearable Haptics. *Adv. Mater.* **32**, 2002564 (2020).
11. Besse, N., Rosset, S., Zarate, J. J. & Shea, H. Flexible Active Skin: Large Reconfigurable Arrays of Individually Addressed Shape Memory Polymer Actuators. *Adv. Mater. Technol.* **2**, 1–8 (2017).
12. Yu, X. *et al.* Skin-integrated wireless haptic interfaces for virtual and augmented reality. *Nature* **575**, 473–479 (2019).
13. Chen, S., Chen, Y., Yang, J., Han, T. & Yao, S. Skin-integrated stretchable actuators toward skin-compatible haptic feedback and closed-loop human-machine interactions. *npj Flex. Electron.* **7**, 1–12 (2023).
14. Jang, S.-Y. *et al.* Dynamically reconfigurable shape-morphing and tactile display via hydraulically coupled mergeable and splittable PVC gel actuator. *Sci. Adv.* **10**, 1–13 (2024).
15. Youn, J. H. *et al.* Skin-attached haptic patch for versatile and augmented tactile interaction. *Sci. Adv.* **11**, 1–11 (2025).
