# Supplementary material for: Soft tactile chip with in-situ sensing for haptic rendering and reverse feedback enhanced gross to fine teleoperation
Source: Nat Commun. 2026 May 11;17:6295. doi: 10.1038/s41467-026-73000-8 (PMC13376641; doi:10.1038/s41467-026-73000-8)
Supplement: Supplementary file 2 — Description of Additional Supplementary Files [file 41467_2026_73000_MOESM2_ESM.pdf]

<u>Description of Additional Supplementary Files</u>

File Name: Supplementary Movie 1

Description: Characterizations of sensing and feedback functions of TACHIP

File Name: Supplementary Movie 2

Description: Subjective experiments for haptic feedback with in-situ feedback monitoring

File Name: Supplementary Movie 3

Description: Pneumatic actuator array enabled micro-manipulation

File Name: Supplementary Movie 4

Description: Demonstration of haptic rendering and reverse feedback enhanced gross to fine teleoperation
