# Supplementary material for: Soft tactile chip with in-situ sensing for haptic rendering and reverse feedback enhanced gross to fine teleoperation
Source: Nat Commun. 2026 May 11;17:6295. doi: 10.1038/s41467-026-73000-8 (PMC13376641; doi:10.1038/s41467-026-73000-8)
Supplement: Supplementary file 7 — Transparent Peer Review file [file 41467_2026_73000_MOESM7_ESM.pdf]

nature portfolio

Peer Review File

Soft tactile chip with in-situ sensing for haptic rendering and reverse feedback enhanced gross to fine teleoperation

Corresponding Author: Professor Tao Chen

This file contains all reviewer reports in order by version, followed by all author rebuttals in order by version.

Version 1:

Reviewer comments:

Reviewer #1

(Remarks to the Author)
This paper presents a device that provides remote tactile interaction by fabricating sensors in situ within the actuator structure, and that is capable of measuring both contact pressure and temperature. With that in mind, the reviewer would like to ask a few questions.

1) It seems that contact pressure sensors filled with air pressure and temperature sensors filled with liquid metal using PDMS have been proposed in the past, as shown below. Compared to these, what is the novelty of this research?

（Contact pressure sensor）
[1] Transparent Pneumatic Tactile Sensors for Soft Biomedical Robotics — S. Zhao et al., Sensors (MDPI), 2023.
[2] Flexible Pressure Sensor with PDMS Micro-/Pyramid Structures (L. Ma et al., J. Mater. Chem. C / RSC, 2018)

（Temperature sensor）
[3] Pan Zhang et al., “Liquid-Metal-Based Stretchable Dual-Parameter Sensor for Simultaneous Detection of Deformation and Temperature”, Advanced Materials Technologies, 2022 (DOI: 10.1002/admt.202201264).
[4] Zi Ye, Qian Li, Renchang Zhang, Pan Zhang, Lin Gui, “Fabrication of a thin PDMS film with complex liquid metal electrodes embedded and its application as skin sensors”, RSC Advances, 2022.

2) There have also been reports of integrating contact pressure sensors into soft pneumatic actuators. What are the advantages and differences of your proposing device?

[5] A Self-Sensing Soft Pneumatic Actuator with Closed-Loop Control for Haptic Feedback / M. Yu et al., 2022

[6] Development and Integration of Carbon–PDMS Sensors for Motion Sensing in Soft Pneumatic Actuators / K. Ma et al., 2024 (MDPI)

3) Will inflating the pneumatic actuator affect the characteristics or accuracy of the temperature sensor?

Reviewer #2

(Remarks to the Author)
1. The manuscript identifies two key problems motivating this work, but additional limitations exist that are not sufficiently acknowledged. In particular, the durability of the air channels, the long-term airtight sealing, and potential leakage risks are not addressed. Since any micro-leakage would directly compromise baseline stability and tactile feedback fidelity, please expand the discussion of these issues and provide durability data.

2. The tactile sensing mechanism is described as air-pressure-based, but the response time of the pneumatic channel–sensor loop is not quantified. Real-time haptic teleoperation typically requires <10 ms latency, and pneumatic systems often operate slower. Please provide measured response times, a latency breakdown of the sensing loop, and discuss whether the system meets the requirements for high-frequency fine manipulation.

3. The system is presented as a centimeter-scale lab prototype, yet the exact chip dimensions and the scalability of the design are not clearly stated. Please specify the dimensions of the fabricated tactile chip and discuss the potential challenges of scaling this design either for miniaturization (e.g., surgical applications) or for large-area tactile coverage (e.g., robotic palms).

4. Sensor integration is demonstrated via embedded pneumatic channels. However, it is not clear whether the system uses homogeneous pressure sensors only, or whether any multimodal sensing (e.g., shear, vibration, temperature) is supported. Please clarify the sensing modalities included in the chip and discuss whether sensor fusion could further improve performance.

5. Validation experiments are limited to a small set of gross-to-fine teleoperation tasks with few operators. Please provide more details on the number of human subjects involved, the range of tasks, and whether statistical analysis was performed to support the claims of improved dexterity and task success rate.

6. The paper claims improved gross-to-fine teleoperation, but no benchmarking is presented against other tactile sensing approaches (e.g., capacitive, piezoresistive, optical fiber). Without such comparisons, it is difficult to assess whether the pneumatic chip offers a clear performance advantage. Please include either comparative data and detailed justification.

7. Environmental robustness (e.g., temperature, humidity, long-term drift) is not evaluated. Given the pneumatic principle of the device, such conditions can affect baseline pressure and signal stability. Please discuss this limitation and whether compensation strategies were considered.

8. The system is validated only in a controlled lab setup, but integration into multi-DOF robotic hands, wireless teleoperation platforms, or long-duration tasks is not discussed. Please clarify how the proposed tactile chip could be scaled and practically deployed in more complex systems, and identify potential bottlenecks (e.g., energy efficiency, system-level latency, or mechanical compatibility).

Reviewer #3

(Remarks to the Author)

The manuscript reports a soft “tactile chip” (TACHIP) that co-integrates pneumatic actuation with in-situ sensing for telehaptics. Each chip comprises a five-pixel silicone/PDMS pneumatic array; the PDMS membrane embeds liquid-metal microchannels that serve dual roles—as piezoresistive pressure sensors and as micro-heaters for thermal feedback—while a pectin thin film provides temperature sensing. A flexible printed connector enables rapid system integration. The authors claim that in-situ sensing improves spatial co-registration between actuation and measurement, enabling self-adaptive haptic rendering (e.g., “hard” vs “soft” through pressure modulation) and a quantitative alternative to purely subjective pattern tuning. They further demonstrate fusion of haptic and thermal cues, object-shape recognition when multiple chips are mounted on a gripper, and a “reverse haptic feedback” concept: finger micro-motions on the master side are projected to a slave gripper by treating the five-actuator array as a small parallel manipulator for fine teleoperation. Reported figures include forces up to 1.67 N, operation up to 500 Hz (vibrometer-measured micromotions), and first-round subjective identification rates around 95% for selected patterns; fabrication and a portable control module are described. Overall, the work targets modular, add-on telehaptic capability that couples actuation and sensing in a compact soft system, with demonstrations spanning sensing-assisted feedback design, multimodal feedback, and micro-manipulation within a teleoperation workflow.

Here are my major comments:
1) When units U1–U5 are operated as actuators, the observed variations in voltage (V) are considerably smaller than those recorded when the same elements are used as force sensors. This suggests that the externally applied forces during sensing tests may exceed the actuation forces that the system itself can generate. From a haptic-interface standpoint, the actuation and sensing ranges should ideally be comparable to ensure transparency, accuracy, and repeatability. The authors are encouraged to clarify the relative magnitudes of the applied and generated forces, and to discuss how this mismatch may affect bidirectional performance and overall system fidelity.

2) In the current experiments, the normal forces applied by the human operator appear uncontrolled and are not quantitatively measured. It would be more rigorous to apply a known and measurable normal force—using, for example, a calibrated load cell or force gauge—so that the actual contact force can be compared directly with the sensor outputs of the TACHIP system. This would allow a more quantitative assessment of the sensor accuracy and the fidelity of the haptic feedback.

3) In the experimental results, the baseline voltages of channels V1–V5 differ even when no normal force is applied. The authors should clarify the origin of these offsets—whether they arise from fabrication tolerances, residual stresses in the pneumatic membranes, variations in liquid-metal channel geometry, or electronic drift. Explaining or compensating for these baseline differences would improve the interpretation of the sensing data and the overall reliability of the system.

4) In the high-frequency tests (50 Hz and 100 Hz), the supplementary video appears to be played at approximately 1.5× real

speed. At such frequencies, if the video speed is altered, it should instead be slowed down to allow the physical membrane motions to be perceptible to the human eye. Otherwise, it is difficult to confirm whether the actuators truly deform at the claimed frequency. Furthermore, the rationale for placing an additional object on the actuators during these demonstrations is not explained. The authors should clarify the purpose of the added object—whether it serves as a visual marker, a mechanical load, or part of the experimental measurement

5) The manuscript does not provide any information on the durability or lifetime of the pneumatic actuators and integrated sensors. Have the authors performed life-cycle or fatigue tests to assess performance degradation under repeated actuation? An estimate of the operational lifetime (e.g., number of cycles before noticeable loss of sensitivity or force output) would be valuable for evaluating the device's practical reliability and suitability for long-term teleoperation applications.

6) In the micro-manipulation experiments, the desired trajectories (triangle or square) are presented, but the corresponding actual paths followed by the system are not explicitly shown. It is therefore unclear how the authors evaluated the tracking performance or validated the accuracy of motion execution. From the provided figures, it even appears that the "desired" red path may have been derived from the recorded trajectory itself. For clarity, the desired and actual paths should be plotted together on the same graph, and the experiment should be repeated multiple times (e.g., three to five cycles) to demonstrate the repeatability and precision of the micro-manipulation.

7) The manuscript does not specify the stiffness level of the pneumatic actuators during the micro-manipulation experiments —whether they operated in a soft, medium, or hard state. Was the effective stiffness tunable during manipulation through modulation of the internal pressure, or fixed throughout the test? Since actuator stiffness directly influences positioning accuracy and repeatability, quantitative information on its value and controllability would strengthen the analysis. An explanation of how stiffness variation affects manipulation precision would also provide useful physical insight.

8) It is unclear whether the angular trajectories shown in the micro-manipulation experiments were tracked using feedback of the actual angles or operated purely in open-loop mode. If feedback was employed, the authors should specify the sensing method and control algorithm used (e.g., PID, model-based, or other). If no feedback was applied, it is difficult to understand how the system achieved accurate angular motion, given the nonlinear and compliant nature of pneumatic actuation. Clarifying whether a closed-loop or model-based control strategy was implemented—and providing details of its accuracy—would significantly improve the technical transparency of the work.

9) In the presented micro-manipulation experiments, no external contact force appears to be applied to the tool, suggesting that the tests were conducted under unloaded conditions. In practical applications, however, the manipulator would interact with external objects and thus experience contact forces that could influence accuracy and stability. The authors should verify how such external loading would affect the system's performance, quantify the expected positioning error under realistic contact forces, and discuss the implications for real-world teleoperation or fine-manipulation scenarios.

10) The graph of $\Delta V/V_0$ versus pressure in Fig. 2c provides useful information on sensor performance. However, the origin of the plotted data is unclear. Were these curves obtained from the authors' own fabricated sensors with different design parameters, or are some taken from previously published studies for comparison? Clarifying the source of each dataset and specifying whether all samples were designed and characterized within this work would greatly improve the transparency and credibility of the results.

11) In Fig. 4b, the description of the experiment is unclear. The authors state that "the experimental data for detecting the increasing air pressure and the cyclic inflation are shown," but it is not specified which variable is controlled and which is measured. From the plots, it appears that the upper panel corresponds to stepwise increases in input air pressure (10–20 kPa) to demonstrate sensitivity, while the lower panel shows cyclic inflation–deflation at a fixed pressure (16 kPa) to verify repeatability. This clarification should be explicitly stated in the text or figure caption. The authors should also mention how the pressure was applied (e.g., via piezo pump or external regulator) and whether $\Delta V/V_0$ represents voltage or resistance change from the liquid-metal sensor. Clear identification of the independent (input pressure or actuation cycle) and dependent (sensor output) variables would greatly improve reproducibility and reader understanding.

12) The authors report a response time of approximately 49 ms for the in-situ pressure sensor (Fig. 2d), which corresponds to a bandwidth below roughly 20 Hz. However, later sections (Fig. 4d(ii–iii)) claim pneumatic actuation and vibrometer-measured displacements up to 100–500 Hz. It is unclear how the soft pneumatic pixels—with their compliant membranes and air chambers—can deform and be monitored effectively at such high frequencies when the integrated sensor cannot follow signals faster than about 20 Hz. The authors should clarify whether the 100–500 Hz values refer to the pump drive frequency or the actual membrane motion, provide a frequency-response curve (amplitude and phase or −3 dB bandwidth) of the pixel under defined conditions, and explain whether the 49 ms response was measured under similar loading or if a faster sensing pathway exists for high-frequency operation. Reconciling the sensor dynamics with the claimed actuation bandwidth is necessary to substantiate that the TACHIP can operate reliably in the 100–500 Hz regime.

13) In Fig. 7c(ii), the fitted linear relationship y=1.16+0.63xy = 1.16 + 0.63xy=1.16+0.63x appears to match the experimental data more closely than the simulation results. However, this fit implies a non-zero displacement (y = 1.16) at zero input pressure (x = 0), which is physically inconsistent. Displacement should vanish in the absence of applied pressure. The

authors should clarify whether this offset arises from measurement noise, initial membrane pre-strain, or fitting artifacts, and consider constraining the intercept to zero in the regression. Providing an explanation or corrected fit would improve the physical validity of the data interpretation.

Some minor comments:
• The term “fine tuning” should be used consistently throughout the manuscript; please replace all occurrences of “fine-tuning” with “fine tuning.”

• In the Abstract, change “remains challenge” to “remains a challenge.”

• Define PDMS (polydimethylsiloxane) upon its first appearance in the Abstract.

• The sentence “to increase the response time of thermal feedback” is misleading, as higher power would in fact reduce (shorten) the response time. Please revise to reflect the intended meaning.

• In Fig. 5a(i), the text refers to Px, tx, and fx, but these parameters are not visible or labeled in the figure. Please ensure that all referenced quantities are explicitly indicated and described.

• The statement “Two center pneumatic actuators act as support pivots for upper and lower surfaces” requires clarification. What are the stiffness values of the central versus surrounding actuators? If the central actuators function as pivots, they should exhibit greater stiffness; please explain how this condition is achieved.

• When forces are applied to the five pneumatic pixels, a voltage around 700 is recorded. Please specify the unit or scale of this voltage (e.g., mV) in the corresponding axis label.

Reviewer #4

(Remarks to the Author)
This manuscript represents a soft tactile chip (TACHIP) made by in-situ fabrication of sensors and actuators. This chip has both haptic and thermal sensing with high-resolution haptic rendering, equipped with a flexible print circuit. Applying TACHIPs to a teleoperation system can achieve direct haptic rendering. Thanks to TACHIPs, finger micro motions from the primary side can be directly projected into the secondary side. The proposed approach realizes mutual micro manipulation with tactile perception in teleoperation, social network, and virtual training.

This manuscript provided much technical progress, but this reviewer found little scientific contribution in this manuscript.

- What are the noteworthy results?

This manuscript describes many topics, including a soft tactile chip (TACHIP), array-based haptic rendering, haptic and thermal feedback, and micro-motion projection. Thus, this reviewer feels this manuscript is unfocused, and would suggest that the authors focus on scientific contributions.

"Here, we report a soft tactile chip (TACHIP) made by in-situ fabrication of thin film sensors in the membranes of pneumatic actuator (Fig. 1a). This chip is fabricated by flexible pneumatic actuators, featured by bottom polydimethylsiloxane (PDMS) actuation membrane (thickness: 0.3 mm) with encapsulated liquid metal micro channels, and top layer with pectin-based temperature sensing film (thickness: 0.1mm)."
This is a significant technological achievement based on existing methods. This reviewer wonders what novel scientific contributions are in this manuscript. Technological achievement would be transferred to an engineering journal such as IEEE MEMS or Sensors & Actuators.

The authors claim that
"In addition, instead of traditional subjective test, a new methodology of evaluating haptic feedback performance with quantitative and objective method is proposed, TACHIPs with in-situ sensing allows the establishment of haptic feedback sensing database to facilitate the design of feedback pattern through data assisted pre-adjustment."
and
"Owing to the in-situ pressure sensor, as an novel methodology in establishing a more quantitative and objective evaluation standard, the sensing data can be simultaneously recorded for building database during the entire actuation process."
This reviewer wonders what "a new methodology of evaluating haptic feedback performance" implies over traditional tests. With in-situ sensors or external sensors, sensing data is available. Why do in-situ sensors alone enable building the database while external sensors do not?

Discussion seems a summary of the proposed approach. More informative discussion is required.

The authors claim that
"The enlarged data in Fig. 4d(iii) indicates that the vibrational pneumatic haptic feedback at 500 Hz can generate 1.5 \micro m displacement which is still perceivable, according to the Pacinian threshold of humans.

and
"Fig. 4e(ii) illustrates the modality control of pneumatic array done by modulating cycling frequency and input power via program. Thus, reconstruction of SA and FA perception is achieved without additional actuators."
These could be contributions. Exploring these topics in depth would be worthy of scientific contributions.

Terminology "master-slate" should be replaced by "primary/secondary", "primary/replica", or "leader/follower".

- Will the work be of significance to the field and related fields? How does it compare to the established literature? If the work is not original, please provide relevant references.

This reviewer wonders if "reverse feedback" is coincident with "bilateral feedback", which has been proposed over the past four decades in teleoperation field. Also, "in-situ sensing" seems similar to the concept of "collocation" of sensors and actuators

- Does the work support the conclusions and claims, or is additional evidence needed?

"As an example of TACHIP, several key parameters are concluded from systematic analysis of the subjective test data and the corresponding sensing signals. Specifically, as shown in Fig. 5a(i), actuation pressure (Px, x is the index of actuator), differential pressure(\Delta Px-y, x and y are the indexes of actuators), inflation time (tx), gap time (\Delta tx-y), and frequency (fx), etc., are essential for static and dynamic feedback."
This reviewer wonders how these key parameters are systematically selected out of many potential quantities. It seems that the authors select these key parameters through their intuition. If not, show the procedure to select the key parameters.

"Differential pressures among actuators affect the perception regeneration of shape, geometry, etc."
This reviewer wonders how the authors can relate the differential pressures to perceptions. This review could not find any description supporting this claim. This question applies to the following sentence: "Feelings of texture and other dynamic events also depend on frequency of actuation".

- Are there any flaws in the data analysis, interpretation and conclusions? - Do these prohibit publication or require revision?

- Is the methodology sound? Does the work meet the expected standards in your field?

"providing target coordinates, the corresponding displacements of pneumatic actuators and their required input air pressure can be calculated."
Relationship between target coordinates and actuator displacements is a simple geometric equations, which can be avoided from the main text.

This reviewer wonders why "Newton downhill method" is applied to solve these geometric equations. Automatic differentiation could be applied to such simple equations.

- Is there enough detail provided in the methods for the work to be reproduced?

"Eleven objects, including sphere, cylinder, frustum, cube with four orientations, trapezoid with four orientations, were selected for collecting the training dataset."
Here only geometric shapes are focused on. This reviewer wonders what happens when natural shapes with much irregularity are applied.

"Three hardness levels were tested by changing the air pressure induced resistive force of pneumatic chamber, including hard, medium, and soft, are labelled as pattern 1, 2, and 3, as shown in Fig. 5c(ii)."
This reviewer wonders how much these softness values are. Please show softness quantitatively (Young's moduli or Shore A hardness values).

"Fusion of haptic and thermal feedback augmented teleoperation."
"In general, fusion of thermal and haptic feedback with the varied detail information across the area can give enhanced perception recreation during precise teleoperation."
Unfortunately, only one description on "enhanced perception recreation" can be found in this manuscript. This reviewer wonders how this can be generalized.

"According to this information, the modification of the program can be done for all haptic feedback patterns to ensure better performance."
This reviewer wonders how this modification can be achieved.

"As shown in Fig. 8c(i), the key units for identifying the directions of the finger twisting motions are highlighted as black dots."

This reviewer wonders what "key units" implies. Are "key units" same as "key parameters"?

Version 2:

Reviewer comments:

Reviewer #1

(Remarks to the Author)
Thank you for your detailed response.
Your responses were sufficient to clear up any questions I had.

Reviewer #2

(Remarks to the Author)
The revised version of the manuscript shows substantial improvements in clarity, experimental detail, and system integration. However, several technical and validation aspects remain incomplete and should be strengthened before the paper can meet the standards of a Nature-tier publication. The following points summarize the remaining issues to be addressed:

The description of the liquid-metal sensor's voltage divider lacks quantitative justification for linearity and thermal stability. The authors should clarify how resistor matching, calibration, or compensation mitigates nonlinearity and temperature drift during operation.

The pneumatic actuator characterization should include quantitative error margins and hysteresis curves. Additional calibration data are needed to validate the claimed dynamic response and ensure reproducible pressure–force behavior.

The paper reports tunable thermal feedback but lacks control-loop performance data. The authors should provide evidence of temperature stability, overshoot suppression, and long-term reliability under repeated cycles.

Since multiple sensors and actuators are integrated, the manuscript should quantify any potential electrical or mechanical crosstalk between pressure, temperature, and motion channels to confirm system-level fidelity.

Comparative benchmarking is essential. The authors should position TACHIP's performance (force range, frequency bandwidth, sensing resolution) against state-of-the-art haptic platforms or recent multifunctional tactile devices.

While flexibility and modularity are highlighted, the practical aspects of large-scale fabrication, long-term reliability, and material degradation are not experimentally validated. A brief discussion or early test data should be added.

The dataset, signal-processing pipeline, and raw calibration curves need to be made publicly accessible or provided in Supplementary Information for verification.

Reviewer #3

(Remarks to the Author)
While the authors have addressed several presentation issues and added new data, a number of core concerns remain unresolved. In particular, the force fidelity, lifetime extrapolation, and micro-manipulation accuracy are still not supported by direct measurements. These issues do not necessarily preclude publication, but they require either additional validation or explicit narrowing of the claims to match the demonstrated capabilities.

I am not satisfied with the response to my first comment. While safety considerations may indeed necessitate force scaling (i.e., not rendering a 1:1 force magnitude), they do not support the statement that accurate quantitative force projection is only feasible within a safe force range. Fidelity is determined by the device's dynamic range, saturation, bandwidth, and nonlinearities, and accuracy should be defined relative to an explicit scaling law if such scaling is employed. Moreover, the manuscript does not describe any implemented force projection strategy (e.g., the claimed 5:1–10:1 ratios), nor does it explain how such scaling was applied in the reported experiments.
The force-gauge versus TACHIP comparison further suggests that the TACHIP signal drifts relative to the actual applied force, with at least two intervals in which the externally applied force is increased to re-align with the TACHIP output before being increased again. This behavior indicates a non-trivial mismatch between sensed and applied forces. Therefore, validation using a controlled external force sensor is essential, particularly for a top-tier journal such as Nature.

The added cyclic test demonstrates short-term endurance ($\approx 4\times10^4$ cycles) without obvious degradation, which is valuable. However, the conclusion that the actuators and sensors can "last for millions of cycles" is not supported by the presented data and appears to be an extrapolation without a defined degradation model, failure criterion, or accelerated testing. The manuscript should either quantify degradation metrics and justify this extrapolation or moderate the lifetime claim accordingly.

Finally, the dashed trajectories in Fig. 6(d) appear to be reconstructed from actuator pressure inputs via the kinematic model

rather than obtained from direct measurement of the end-effector motion. As no independent position sensing is described, these curves do not represent experimentally recorded paths. Consequently, this form of reporting cannot reveal the true physical accuracy of the manipulation and may give a misleading impression of tracking performance, since model-reconstructed trajectories inherently mask errors arising from compliance, slip, contact forces, and re-gripping.

Reviewer #4

(Remarks to the Author)
This revised manuscript represents a soft tactile chip (TACHIP) made by in-situ fabrication of sensors and actuators. The manuscript was revised to make clear the novel scientific contributions, haptic rendering enhanced by in-situ sensing and ulti-DOF micro-manipulation enabled through reverse haptic feedback. The authors conducted additional experiments demonstrating SA+FA perception, together with a thorough revision of texts and figures. The authors successfully mention that obtaining both subjective perception and sensing data with in-situ sensor reduces time consumption during dataset building. This reviewer raised several technical concerns as well as descriptive concerns; all of them were clarified. This reviewer is satisfied with the revision of this manuscript.

Version 3:

Reviewer comments:

Reviewer #3

(Remarks to the Author)
Thanks for your responses. Now I am satisfied with your answers. Good job, good luck!

Open Access This Peer Review File is licensed under a Creative Commons Attribution 4.0 International License, which permits use, sharing, adaptation, distribution and reproduction in any medium or format, as long as you give appropriate credit to the original author(s) and the source, provide a link to the Creative Commons license, and indicate if changes were made.
In cases where reviewers are anonymous, credit should be given to 'Anonymous Referee' and the source.
The images or other third party material in this Peer Review File are included in the article’s Creative Commons license, unless indicated otherwise in a credit line to the material. If material is not included in the article’s Creative Commons license and your intended use is not permitted by statutory regulation or exceeds the permitted use, you will need to obtain permission directly from the copyright holder.
To view a copy of this license, visit https://creativecommons.org/licenses/by/4.0/

Dear Reviewers:

Your comments are very valuable, insightful, and helpful for revising and improving our manuscript. We highly appreciate these rigorous inspections which help us to fulfill the standard in scientific research. We have studied all of the comments carefully and made considerable number of revisions which we hope can address your concerns and meet with your approval. We also would like to provide further explanation or experimental data if additional concerns are raised. Briefly speaking, we have done extra experiments to collect new data, and added the additional discussion into the main manuscript in response to reviewers' comments. We have revised Fig. 2, 3, 4, 5, 6, 7, and Fig. 8, and added supplementary Fig. 3, 6, 8, 12, 13, 14, 15, 17, 21, and modified supplementary movie 1. All of the revisions in the main manuscript and the supplementary information are marked in red, and the point-by-point response to the reviewers' comments are provided as following:

REVIEWER COMMENTS

Reviewer #1 (Remarks to the Author):

This paper presents a device that provides remote tactile interaction by fabricating sensors in situ within the actuator structure, and that is capable of measuring both contact pressure and temperature. With that in mind, the reviewer would like to ask a few questions.

1) It seems that contact pressure sensors filled with air pressure and temperature sensors filled with liquid metal using PDMS have been proposed in the past, as shown below. Compared to these, what is the novelty of this research?

（Contact pressure sensor）

[1] Transparent Pneumatic Tactile Sensors for Soft Biomedical Robotics — S. Zhao et al., Sensors (MDPI), 2023.

[2] Flexible Pressure Sensor with PDMS Micro-/Pyramid Structures (L. Ma et al., J. Mater. Chem. C / RSC, 2018)

（Temperature sensor）

[3] Pan Zhang et al., "Liquid-Metal-Based Stretchable Dual-Parameter Sensor for Simultaneous Detection of Deformation and Temperature", Advanced Materials Technologies, 2022 (DOI:

10.1002/admt.202201264).

[4] Zi Ye, Qian Li, Renchang Zhang, Pan Zhang, Lin Gui, “Fabrication of a thin PDMS film with complex liquid metal electrodes embedded and its application as skin sensors”, RSC Advances, 2022.

We thank a lot for the valuable comments with these good references. We need to clarify the potential misunderstanding about the sensor designs and the sensing working mechanisms of our proposed work. We also apologize if there is any misleading description to cause the confusion, and we have revised the manuscript accordingly.

Firstly, for the basic sensing working mechanism of pressure sensor, it consists of PDMS encapsulated liquid metal micro channels which are fabricated by photolithography. This pressure sensor can work individually as a thin film tactile sensor, which utilized piezoresistive effect induced by mechanical deformation on liquid metal channel during pressing. In this work, the pneumatic actuation and the contact force applied on pneumatic actuator need to be detected. Therefore, we integrated the above liquid metal thin film pressure sensor into the actuation membrane in pneumatic actuator.

For temperature sensor, it was made of pectin and $CaCl_2$ solidified thin films. Pectin has the structural complex of acid-rich polysaccharides which can bind ions, such as Ca2+. As the temperature increases, the ionic conduction will increase due to the exponential reduction of the cross-linking of pectin molecules. This thin film also can work individually as temperature sensor. We have integrated this film into the actuation membrane of pneumatic actuator to achieve temperature sensing and act a temperature reference to compensate the baseline drift of the liquid metal based piezoresistive pressure sensor.

The abovementioned sensors used in our manuscript are also reported several times. We also have cited those references in introduction section. In fact, the fabrication of sensor and actuator are not the novelties claimed in our original manuscript. The main contribution of our works with special designed modular TACHIP can be considered as follows: (1) In-situ sensing enhanced haptic rendering and facilitated haptic pattern programming with a new methodology; (2) Reverse haptic feedback enabled multi-DOF micro-manipulation; (3) Temperature and pressure sensing fusion of TACHIP; (4) all components, including TACHIP, pumps, wireless microcontroller, are customized as standard PCB or flexible PCB modules with quick plugin cable. Unlike the highly customized devices, scalable and universal design of TACHIP system is also ready to be integrated into data glove, and other wearable platforms. The detailed explanation can be found in the response of comment 2.

2) There have also been reports of integrating contact pressure sensors into soft pneumatic actuators. What are the advantages and differences of your proposing device?

[5] A Self-Sensing Soft Pneumatic Actuator with Closed-Loop Control for Haptic Feedback / M. Yu et al., 2022

[6] Development and Integration of Carbon–PDMS Sensors for Motion Sensing in Soft Pneumatic Actuators / K. Ma et al., 2024 (MDPI)

We thank a lot for the careful inspection and the valuable comment with two good references. We are also familiar with the work in reference 5 (the corresponding reference has been added into the manuscript). In fact, we also have published several works similar to reference 5 and 6.

1. M. Zhu, Z. Sun, C. Lee, Soft Modular Glove with Multimodal Sensing and Augmented Haptic Feedback Enabled by Materials' Multifunctionalities, ACS Nano 16 (2022) 14097–14110. https://doi.org/10.1021/acsnano.2c04043.

2. T. Jin, Z. Sun, L. Li, Q. Zhang, M. Zhu, Z. Zhang, G. Yuan, T. Chen, Y. Tian, X. Hou, C. Lee, Triboelectric nanogenerator sensors for soft robotics aiming at digital twin applications, Nat. Commun. 11 (2020) 5381. https://doi.org/10.1038/s41467-020-19059-3.

Therefore, the novelty of our new work is not simply integrating sensing and feedback units to make a combined hardware. The main advantages or scientific contributions are follows:

(1) **In-situ sensing enhanced haptic rendering and facilitated haptic pattern programming (gross operation stage).**

The enhanced haptic rendering refers to several aspects, including self-adaptive hardness regeneration via tunable SA perception by in-situ sensing, pressure and frequency modulation for replicating shape/texture/dynamic motion through fusion of SA and FA perception, etc.

On the other hand, the facilitated haptic pattern programming shows a new methodology to quantify those perceptions for continuous improvement of actuation coding of SA and FA related stimuli. The general purpose of haptic feedback is to regenerate tactile perception as real as possible. However, except building a large test equipment externally, such as expensive laser profiler, it lacks quantitative standard with efficient evaluation tool to analyze and illustrate the haptic feedback patterns designed by programs for mimicking the tactile perception from specific event or object. The work shown in reference 5 is only dealing with close-loop control. In our opinion, the fundamental issue about the performance of the haptic feedback devices and the corresponding feedback programs is the actual stimuli perceived by users. However, skin perception is a quite subjective aspect which may vary with different ages, genders, or even skin conditions. The traditional solution for designing and evaluating haptic feedback pattern or actuation algorithms includes repeating subjective tests and program adjustment with those volunteers, until the best performance is achieved. It is quite time and personnel consuming, especially for product level practical applications in which there may be thousands of

haptic feedback programs need to be verified. Thus, a new protocol/methodology of designing and evaluating haptic feedback program for haptic feedback research is needed. The in-situ sensing should be further investigated as quantitative tool for database collection, and we proposed several key parameters (e.g., gap time, differential pressure, etc.) in this preliminary research. With in-situ sensing capability of haptic actuator, the established sensing database with key parameters can be analyzed and linked to the coding of actuation program. Although subjective tests are still required to build the database at early stage, the involvement of cyclic subjective tests for each pattern can be significantly reduced by utilizing real-time sensing data. As the database keeps accumulating, less or even no subjective tests are needed by cross-checking the similarities between recorded and new haptic feedback patterns.

(2) **Reverse haptic feedback enabled multi-DOF micro-manipulation (fine operation stage).**

Differ from bilateral feedback, we adopted asynchronous revers haptic feedback, together with the array design of TACHIP, to realize a parallel manipulator inspired multi-DOF micro-manipulation which can be teleoperated by hand's intuitive micro-motion. With the aid of our TACHIP and its quick setup process, two TACHIPs with pneumatic actuator array on the gripper can form a classic parallel manipulator structure (three revolute-prismatic-spherical joint/universal-spherical joint, 3-RPS/US). Without modifying classic robotic gripper with expensive multi-DOF actuators, TACHIP with special array design and in-situ sensing as a quick add-on component, can be deployed to provide multi-DOF manipulation to the "last centimeter" of teleoperation.

(3) **Temperature and pressure sensing fusion of TACHIP**.

Following the in-situ sensing design concept, we also developed a pectin-based temperature sensing film, embedded in actuation membrane of pneumatic actuator. This temperature sensing film is immune from the mechanical stimuli, with relative good temperature sensitivity. By leveraging sensing fusion concept of TACHIP, a single temperature sensor can collaborate with pressure mapping information from tactile sensor array and perform approximated temperature mapping, which is important for precise control of haptic and thermal feedback region on another TACHIP.

Besides, there are also advantages that come from engineering achievements. As can be seen in Figure 2, the entire TACHIP system is ready for use in different commercial robots. All components, including TACHIP, pumps, microcontroller with Bluetooth, are customized as standard PCB or flexible PCB modules with quick plugin cables, which are convenient for fast deployment or packing. The whole setup time is less than 2 minutes for most robotic grippers. Unlike the highly customized devices, scalable and universal design of TACHIP system can also be integrated into data glove, and other wearable platforms. Instead of simple verification in controlled laboratory environments, the system was also tested or demonstrated at public EXPO and industrial robot manufacturer.

3) Will inflating the pneumatic actuator affect the characteristics or accuracy of the temperature sensor?

We thank a lot for the careful inspection and valuable comment. As can be seen in the continuous temperature sensing data shown in figure below, we have applied the presses via three objects, including room temperature object (20℃, empty test tube), hot object (33℃, finger), and cold object (13℃, test tube with cold water). According to the pressing of room temperature object, the mechanical contact or even deformation did not affect the temperature sensing signals. As another verification, the supplementary video 1 also demonstrates that the placing or removal of hot beaker did not generate additional interference to the sensing signal, it was even stable under the unintentional lifting induced deformation.

The main reason of this stability attributes to the fundamental working mechanism which differs from regular flexible temperature sensors. Pectin has the structural complex of acid-rich polysaccharides which can bind ions, such as Ca2+. As the temperature increasing, the ionic conduction will increase due to the exponential reduction of the cross-linking of pectin molecules. In the meantime, this kind of temperature sensing mechanism leads to a much lower sensitivity to mechanical stimuli, such as bending and pressing. Therefore, it is a favorable material for fabricating in-situ temperature sensors in the structure of TACHIP.

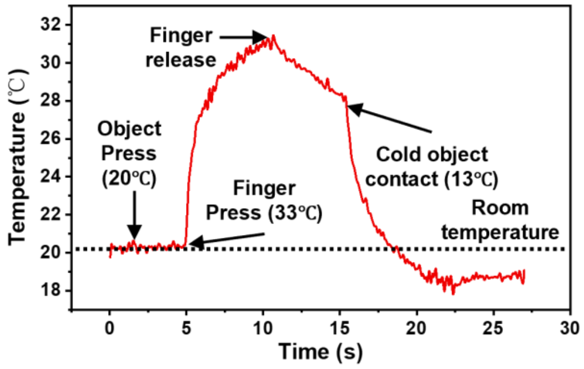

Fig. R1. Stability of temperature sensing under mechanical stimulation.

Reviewer #2 (Remarks to the Author):

1. The manuscript identifies two key problems motivating this work, but additional limitations exist that are not sufficiently acknowledged. In particular, the durability of the air channels, the long-term airtight sealing, and potential leakage risks are not addressed. Since any micro-leakage would directly compromise baseline stability and tactile feedback fidelity, please expand the discussion of these issues and provide durability data.

We thank a lot for the careful inspection and valuable comment. We have tested an old TACHIP (fabricated one year ago) with continuous cyclic actuation for over 20 hours under the constant actuation parameters (50% of input power of pump, 2 seconds/cycle), and recorded the sensing data at different stages to evaluate variations of performance. As the input parameters of piezo pump is fixed, the leakage of actuator can then be detected by sensors. The experimental data has been added accordingly with the zoom-in data of pressure sensing for the respective stages. To double check the reliability of liquid metal pressure sensor in durability test, we have also applied commercial pressure meter to verify the real-time input pressure of the pneumatic system. It can be seen that there is little

degradation of general performance (displacement and force) over long-term and cyclic usage.

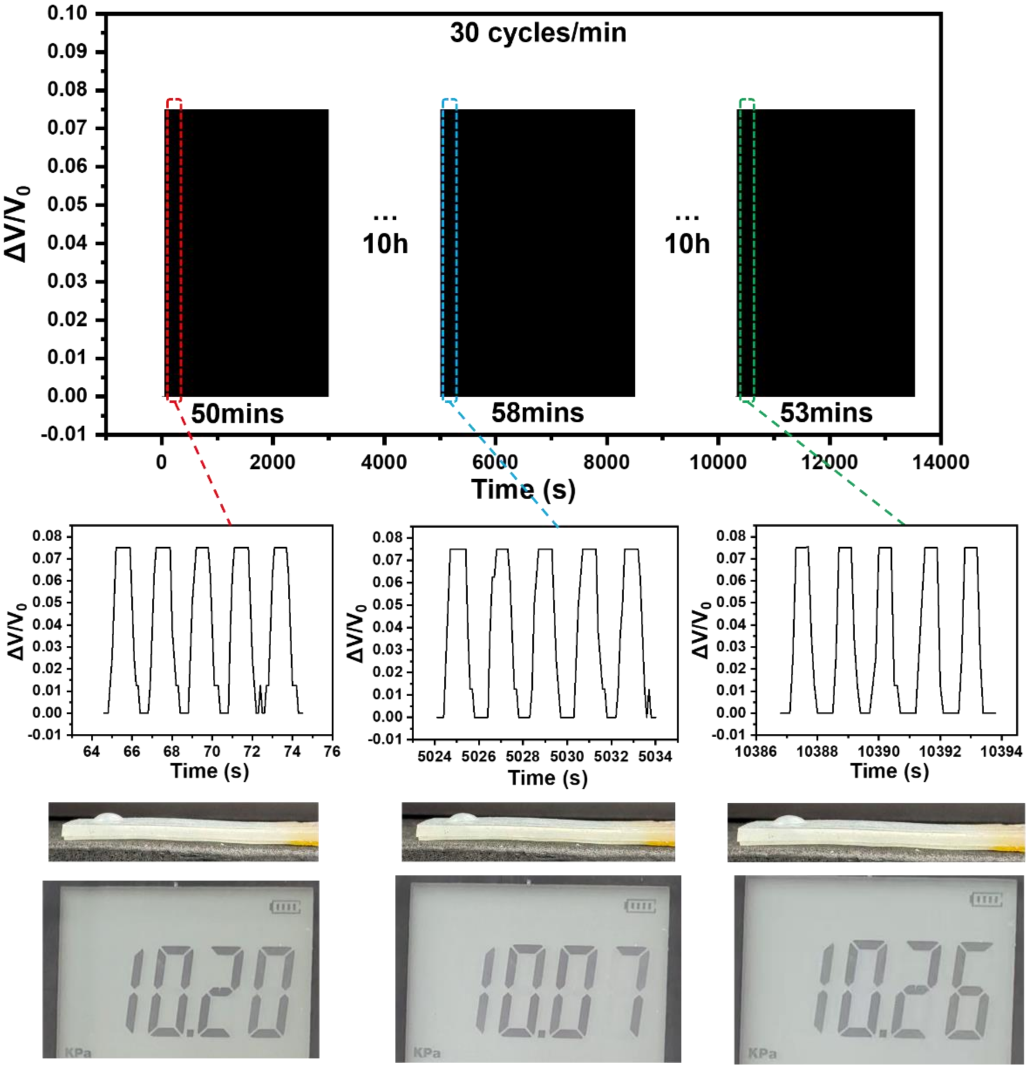

Fig. R2. Cyclic test results of pneumatic actuation and in-situ sensing within 24 hours (30 cycles/min, 50% of input power of pump). The zoom-in data of in-situ sensing, the real photos, and the manometer monitored actual pressures are shown below.

2. The tactile sensing mechanism is described as air-pressure-based, but the response time of the pneumatic channel–sensor loop is not quantified. Real-time haptic teleoperation typically requires <10 ms latency, and pneumatic systems often operate slower. Please provide measured response times, a latency breakdown of the sensing loop, and discuss whether the system meets the requirements for high-frequency fine manipulation.

We thank a lot for the insightful comment and useful suggestion. We completely agree that the delay of tactile communication may cause spatiotemporal deviation of perception and lead to high risk of failure of operations. A latency of 10ms is truly a high requirement not only for haptic feedback device, but also for those flexible tactile sensors. It will be our ultimate goal for chasing. After a literature

survey, it is suggested that the requirements of system latency are varied based on specific tasks, which range from few ms to about 300 ms. The human response times to a step change in position or force are also within few hundreds of milliseconds (doi: 10.1109/TMRB.2024.3349612).

On the other hand, we also found that high frequency operations rarely happen at current stage, except for surface texture or sliding sensing. According to massive number of experiments done by multiple participants, a system latency of 100-200 ms is still reported as acceptable for most of typical operations. Especially for fine teleoperation, the users were usually acting quite slowly to avoid misalignment. Additionally, based on survey, fine manipulations during teleoperation are usually low frequency operations, such as surgical operation, biomedical experiment, precise assembly, and remote rescue/repairing, etc., to avoid potential risk. It is also true that pneumatic actuation usually possesses lower response time compared to other cutaneous feedback techniques. The main purpose of fine manipulation is to utilize TACHIPs enable parallel manipulator structure for adding more DoFs to normal robotic gripper, and use TACHIPs at controller side for manual teleoperation, rather than an automated high frequency manipulator. In this case, we believe that the frequencies of human motions are still within few Hz range. Therefore, the system is considered as suitable for the requirements of fine manipulation. We are also willing to discuss further if the detailed description or application of high-frequency fine manipulation can be provided by the reviewer.

We have done extra experiments to quantify the latency of sensing and feedback loop. Specifically, we placed both sensor and actuator on a commercial pressure sensor with two sensing units, and mounted at force gauge. The sensing and feedback latency can then be evaluated by analyzing the delay between pressing and actuation, which is less than 200 ms based on current setup of electronics.

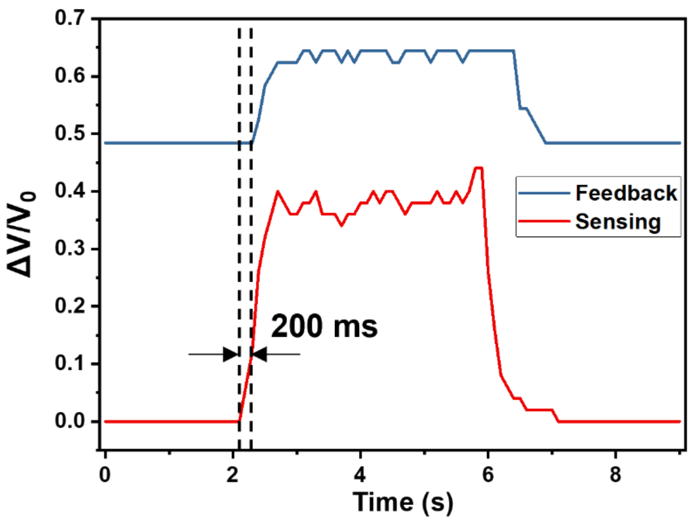

Fig. R3. Operational latency between in-situ sensing and the corresponding pneumatic feedback for a tactile stimulation.

Overall, we will keep working on the development of sensing and feedback techniques with multi-

modal tactile communication, high durability, and low latency. However, it still requires compromise or balance when considering these factors at current stage. Again, we highly appreciate this important issue raised by the reviewer, and will carefully consider it in our future research.

3. The system is presented as a centimeter-scale lab prototype, yet the exact chip dimensions and the scalability of the design are not clearly stated. Please specify the dimensions of the fabricated tactile chip and discuss the potential challenges of scaling this design either for miniaturization (e.g., surgical applications) or for large-area tactile coverage (e.g., robotic palms).

We thank a lot for the valuable suggestions. The exact chip dimensions have been stated in the original manuscript, located at the beginning of "Design and working principle of TACHIP" section. As an updated version, the exact chip dimensions are listed as follows: 35 mm (length) × 20 mm (width) × 2 mm (thickness). The pneumatic actuator has a diameter of 4.5 mm and a height of 0.8mm.

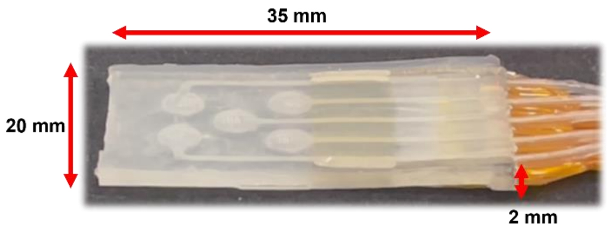

Fig. R4. Actual dimension of the as-fabricated TACHIP.

For large-area tactile coverage, the increased number of pneumatic actuators can be fabricated by using larger mold prepared by either 3D printing or industrial machining. However, the route design of pneumatic actuation may require 3D structure to avoid the overlap. For pump system, although the piezo pump is quite small, massive number of TAHIPs for scalable deployment may also bring size and power consumption issues. We may use fewer pumps with some customized mini switches, but it may lead to stability issue of input pressure. In terms of sensor, the increasing of sensing unit can simply be done by direct inkjet writing or screen-printing techniques. Additionally, it is also necessary to understand the actual needs for different applications, e.g., whether the large-are tactile coverage indicates high density of sensors and actuators, or larger size of individual sensor and actuator.

For miniaturization, the potential challenge of pneumatic actuator is the width of the air tube. As the pneumatic actuator becomes very small, we need to customize the air tube with micro-fabrication process if an array-based TACHIP is required on the surgical tools, such as forceps in Da Vinci surgical system. For sensing unit, the current photolithography approach of fabrication micro-channels is suitable for miniaturization.

4. Sensor integration is demonstrated via embedded pneumatic channels. However, it is not clear whether the system uses homogeneous pressure sensors only, or whether any multimodal sensing (e.g., shear, vibration, temperature) is supported. Please clarify the sensing modalities included in the chip and discuss whether sensor fusion could further improve performance.

We thank a lot for the careful inspection and valuable comment. In the as-fabricated TACHIP, two in-situ sensors are employed, including a liquid-metal based pressure sensor, and a pectin-based temperature sensor. For liquid-metal based pressure sensors, it only acts as a pressure sensor during sensing process, but it can be utilized as thermal haptic feedback element by external power supply during feedback process. In the meantime, we can still achieve certain levels of shear force sensing by analyzing the pressure distribution among all of five sensors. However, it requires uniform contact surface of object to the TACHIP. For pectin-based temperature sensor, it only response to temperature variations, and is immune from mechanical stimulus.

Fusion of pressure and temperature sensors provides several advantages. Firstly, the pressure distribution information can reduce the required number of temperature sensors. Currently, most of the pressure and temperature sensing arrays adopt the design of multi-pixel pressure and temperature sensing units at the same time, which usually requires multiple readouts for both signals. In practical application of haptic sensing and feedback, it may not be necessary for high resolution of temperature detection, due to thermal radiation and the limitation of human skin's perception to temperature information. With the concept of minimalistic design and the aid of five pressure sensors on TACHIP, a single temperature sensing layer may also realize the mapping of temperature distribution via a reasonable approximation done by fusing of temperature data and pressure distribution data. Secondly, piezoresistive pressure sensors usually suffer from temperature induced drifting issue. With the aid of temperature sensor which is immune from mechanical stimulus, the drifting data can be compensated according to the temperature information and the data of temperature dependency acquired before. These discussions have been included in the manuscript, such as descriptions in Fig. 3h and Fig. 2f and 2g (revised version).

5. Validation experiments are limited to a small set of gross-to-fine teleoperation tasks with few operators. Please provide more details on the number of human subjects involved, the range of tasks, and whether statistical analysis was performed to support the claims of improved dexterity and task success rate.

We thank a lot for the valuable suggestions. We have done the extra experiments which evaluated more types of operation tasks with more volunteers. To evaluate the improvement in success rate of teleoperation, tests of teleoperations were conducted by selecting six participants to perform six classic teleoperation tasks. For each task, the participant was asked to perform teleoperation five times with TACHIP and five times without TACHIP. The number and the reasons of failures were recorded for statistical analysis. Specifically, 180 teleoperations with TACHIP and 180 teleoperations without TACHIP have been performed by six participants with equal distributed works. The experimental results are shown in figure below.

Moreover, we have brought the entire TACHIP integrated teleoperation system (shown in the last demonstration of manuscript) into technical EXPO. To verify effectiveness, we have kept the left side

with no TACHIP, and the right side with TACHIPs. Many visitors, including children and seniors, have tried this system for their own tasks. This system has been used continuously for two days, and most of the visitors have made positive comments about the general performance.

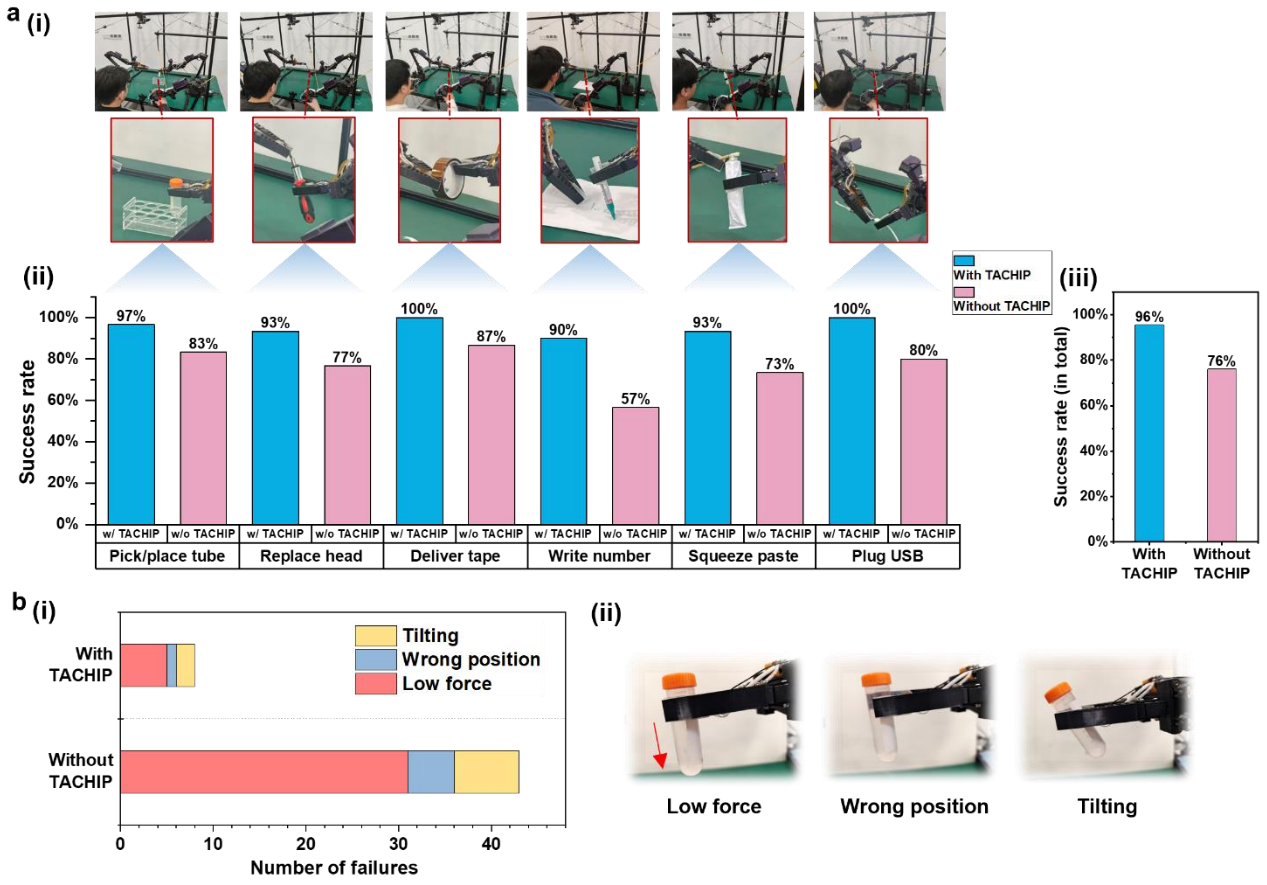

Fig. R5. Evaluation of TACHIP assisted teleoperation tasks. a Comparisons of success rates of different teleoperation tasks performed with and without TACHIPs, (i) Real photos of the setup and the zoom-in views of six tasks, including pick and place of tube, replace head, deliver tape, write number, squeeze paste, and plug USB, (ii) the results of success rates comparisons for the corresponding teleoperation tasks with and without TACHIP, (iii) comparison of success rates with and without TACHIP in total. b (i) Statistical data of major failure conditions during the above teleoperations, (ii) illustrations of the major failure conditions.

6. The paper claims improved gross-to-fine teleoperation, but no benchmarking is presented against other tactile sensing approaches (e.g., capacitive, piezoresistive, optical fiber). Without such comparisons, it is difficult to assess whether the pneumatic chip offers a clear performance advantage. Please include either comparative data and detailed justification.

We thank a lot for the valuable suggestions. It is very important to provide a benchmark for better and clearer presentation of the contributions of our reported work. Actually, the benchmarking table is provided in supplementary information of the original submission. It compared the quantitative

performances of haptic feedback and force/temperature sensing, as well as the functionalities. The capacitive, piezoresistive, optical tactile sensors were all included in that table. There are several reasons of making this type of integrated comparison. First of all, the main purpose of our work is to adopt minimalistic concept for developing tactile communication device as multi-functional as possible. Therefore, instead of focusing on fabricating single sensor or actuator with superior performances, we also need to consider the multi-modal functions desired in practical gross to fine teleoperations. Thus, there were various constraints in selection of functional materials and structural design. For instance, liquid metal was selected due to its piezoresistive effect and resistive heating effect for sensing and feedback, and it was easy to be fabricated as in-situ sensor. Elastomeric pneumatic actuator was selected based on softness, capability of both static and dynamic feedback, and relative high output force and displacement. Generally speaking, the individual performance of sensing or actuation may not be as good as the best tactile sensor or haptic feedback actuator. The TACHIP was developed to serve better for our main novelties, including in-situ sensing enabled self-adaptive haptic rendering, in-situ sensing facilitated haptic feedback programming, reverse haptic feedback based micro-manipulation.

Table R1. Comparisons of functionalities and performances of haptic feedback interfaces

References	Maximum feedback force (mN)	Operation frequency (Hz)	Force sensitivity (N^{-1}) (sensing range, N)	Temperature sensitivity $°C^{-1}$ (sensing range, °C)	Thermal feedback	In-situ sensing	Shape feedback	Shear force feedback	Manipulator	Complete flexible
[1]	3000	0-170	/	/	/	/	/	/	/	/
[2]	/	130-230	/	0.3577 (30-70)	Yes	Yes	/	/	/	/
[3]	1300	0-60	0.014 (1-20)	/	/	/	Yes	Partially	/	/
[4]	/	1-1000	0.014 (0.003-0.08)	/	/	/	Yes	Partially	/	/
[5]	100	0-320	/	/	/	/	Yes	Partially	/	/

[6]	1400	0-200	/	/	/	/	Yes	Yes	/	/
[7]	/	/	/	/	/	/	Yes	Partially	/	Yes
[8]	300	0-200	/	/	/	/	/	Yes	/	/
[9]	/	130-230	/	/	Yes	/	/	/	/	/
[10]	300	0-200	/	/	/	/	Yes	Partially	/	Yes
[11]	1000	0.2	/	/	/	/	Yes	/	/	/
[12]	135	100-300	/	/	/	/	Partially	Partially	/	/
[13]	370	/	/	/	/	/	Yes	/	/	/
[14]	2400	0-300	/	/	/	/	Yes	Partially	Yes	Yes
[15]	2200	0-500	2.55 (0-0.6)	/	/	Yes	Yes	Partially	/	/
This work	1670	0-1000	1.59 (0.2-1.76)	0.325	Yes	Yes	Yes	Partially	Yes	Yes

References:

1. Qi, J., Gao, F., Sun, G., Yeo, J. C. & Lim, C. T. HaptGlove—Untethered Pneumatic Glove for Multimode Haptic Feedback in Reality–Virtuality Continuum. *Adv. Sci.* **10**, 1–12 (2023).

2. Sun, Z., Zhu, M., Shan, X. & Lee, C. Augmented tactile-perception and haptic-feedback rings as human-machine interfaces aiming for immersive interactions. *Nat. Commun.* **13**, 5224 (2022).

3. Liu, M. *et al.* Tactile Sensing and Rendering Patch with Dynamic and Static Sensing and Haptic Feedback for Immersive Communication. *ACS Appl. Mater. Interfaces* **16**, 53207–53219 (2024).

4. Jin, H. *et al.* Highly pixelated, untethered tactile interfaces for an ultra-flexible on-skin telehaptic system. *npj Flex. Electron.* **6**, (2022).

5. Shen, V., Rae-Grant, T., Mullenbach, J., Harrison, C. & Shultz, C. Fluid Reality: High-Resolution, Untethered Haptic Gloves using Electroosmotic Pump Arrays. in *Proceedings of the 36th Annual ACM Symposium on User Interface Software and Technology* 1–20 (ACM, 2023).

6. Flavin, M. T. *et al.* Bioelastic state recovery for haptic sensory substitution. *Nature* **635**, 345–352 (2024).

7. Yao, K. *et al.* A fully integrated breathable haptic textile. *Sci. Adv.* **10**, eadq9575 (2024).

8. Ha, K.-H. *et al.* Full freedom-of-motion actuators as advanced haptic interfaces. *Science (80-.).* **387**, 1383–1390 (2025).

9. Oh, J. *et al.* A Liquid Metal Based Multimodal Sensor and Haptic Feedback Device for Thermal and Tactile Sensation Generation in Virtual Reality. *Adv. Funct. Mater.* **31**, 2007772 (2021).

10. Leroy, E., Hinchet, R. & Shea, H. Multimode Hydraulically Amplified Electrostatic Actuators for Wearable Haptics. *Adv. Mater.* **32**, 2002564 (2020).

11. Besse, N., Rosset, S., Zarate, J. J. & Shea, H. Flexible Active Skin: Large Reconfigurable Arrays of Individually Addressed Shape Memory Polymer Actuators. *Adv. Mater. Technol.* **2**, 1–8 (2017).

12. Yu, X. *et al.* Skin-integrated wireless haptic interfaces for virtual and augmented reality. *Nature* **575**, 473–479 (2019).

13. Chen, S., Chen, Y., Yang, J., Han, T. & Yao, S. Skin-integrated stretchable actuators toward skin-compatible haptic feedback and closed-loop human-machine interactions. *npj Flex. Electron.* **7**, 1–12 (2023).

14. Jang, S.-Y. *et al.* Dynamically reconfigurable shape-morphing and tactile display via hydraulically coupled mergeable and splittable PVC gel actuator. *Sci. Adv.* **10**, 1–13 (2024).

15. Youn, J. H. *et al.* Skin-attached haptic patch for versatile and augmented tactile interaction. *Sci. Adv.* **11**, 1–11 (2025).

If this original table of comparative data in supplementary information already has been checked, and did not meet the requirements of the reviewer, we can add a new table based on the suggestions of the reviewer.

7. Environmental robustness (e.g., temperature, humidity, long-term drift) is not evaluated. Given the pneumatic principle of the device, such conditions can affect baseline pressure and signal stability. Please discuss this limitation and whether compensation strategies were considered.

We thank a lot for the careful inspection and valuable suggestions. We have done extra experiments to verify the robustness under long-term cyclic usage, and varied environmental conditions (e.g., temperature and humidity). Based on the test results of millions of cycles, there is almost no drift of the baseline and the sensing signal under the fixed input pressure. For temperature variations, we already have provided the sensing data of temperature dependency in supplementary of our original submissions. It can be seen that there is about 10% of drift for high pressure (80kPa) after increasing the temperature from 30°C to 60°C. We also have done additional experiments of temperature effect to the baseline with larger temperature variations. A 4% of drift was observed from 40°C to 95°C. According to the plots for sensing ranges of both pressure sensing (tactile sensor) and pneumatic monitoring applications, this drift shows less influence on pressure sensing, but has a significant influence on pneumatic monitoring. Therefore, a compensation strategy is indeed needed for high temperature applications. However, as the temperature above 50°C usually cause potential damage to human skin, it is less likely to experience high temperature drifting effect on pneumatic monitoring.

In addition, we have done extra experiments to evaluate the temperature effect to the internal pressure of pneumatic actuator. As can be seen in the Figure, the influence of temperature variations to the pressure and the deformation of the pneumatic actuator is quite small due to the stiffness of the selected elastomer and the size of the air chamber. Similarly, as the temperature above 50°C usually cause potential damage to human skin, it is less likely to operate the pneumatic haptic feedback with such high temperature for human.

Furthermore, we have conducted experiments on humidity issues as well. It can be clearly observed that there is almost no influence on both sensor and actuator, due to the good encapsulation and integrity of the entire TACHIP.

It is correct that there are still limitations regarding environmental conditions. A compensation strategy is needed for high temperatures. It is worth noting that the pectin-based temperature sensor was originally integrated as in-situ sensor which does not respond to mechanical stimuli or humidity change. Therefore, it can be used as reference sensor to recalibrate tactile information.

We have added experimental results of environmental robustness into the supplementary information.

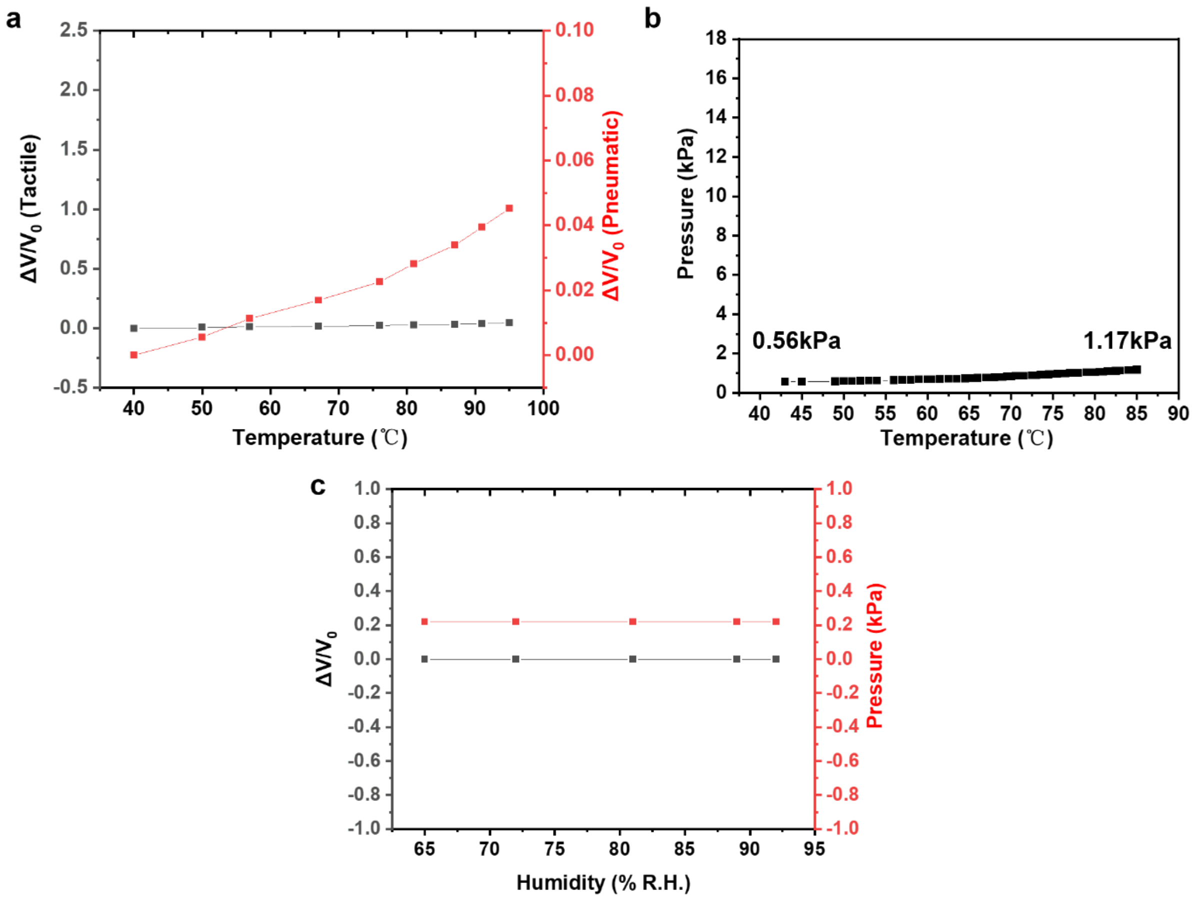

Fig. R6. Reliability tests under environmental variations. a. Temperature effects to the performance of in-situ sensing, left y-axis indicates the general sensing range for tactile sensing application, right y-axis indicates the general sensing range for pneumatic actuation monitoring. b. Temperature effects to the internal pressure of the pneumatic actuator. c. Humidity effects to the performance of in-situ sensing and internal pressure.

8. The system is validated only in a controlled lab setup, but integration into multi-DOF robotic hands, wireless teleoperation platforms, or long-duration tasks is not discussed. Please clarify how the proposed tactile chip could be scaled and practically deployed in more complex systems, and identify potential bottlenecks (e.g., energy efficiency, system-level latency, or mechanical compatibility).

We thank a lot for the careful inspection and valuable comment. We would like to address this comment point-by-point, and provide a general clarification about the scalability and bottlenecks at last.

Firstly, for multi-DoF robotic hands, it is indeed bringing some challenges into the integration of TACHIPs. Currently, we have done the integration of TACHIP in data glove to control the multi-DoF robotic hands (dexterous hand) which had internal tactile sensors already. Tactile sensing and feedback functions was realized by data gloves and robotic hands. We also have tried to integrate TACHIPs on robotic hand, it still works but with several issues need to be concerned. First of all, the surface geometries of fingers and palm are varied a lot among robotic hands from different companies, and we

need 3D printed special clips or elastomeric ring structure for TACHIP to be integrated on different hands with certain curvatures. Secondly, the micro-manipulation function cannot be performed well if the surface curvature is too large to get a flat area, but the other tactile sensing functions will not be affected. Thirdly, for better integration, we may need to customize the FPC for hand structure, but it is easy to be done, and can be universally utilized for most of the dexterous robotic hands.

Secondly, for wireless teleoperation platforms, in fact, our devices were originally operated in wireless condition. In the meantime, the data volume of tactile communications of our devices is quite small. Therefore, we believe that this is an issue related to the current wireless communication technology. As there was 5G enabled teleoperated surgery in hospitals between two remote cities already, the wireless teleoperation may not be a big problem so far, unless there are extremely high requirements of latency.

Thirdly, for long-duration tasks, we have done extra experiments for covering more typical tasks by multiple volunteers. All of those tasks have been repeated multiple times. We believe that this type of continuous, repeating, diverse tasks can be a good verification about long-duration reliability. The statistical results show a high improvement in the operation success rate compared to no TACHIP operations.

In fact, as mentioned earlier, the system was actually tested or demonstrated at public events and industrial manufacturers. We have worked with the top-notched industrial robot manufacturer to provide haptic feedback function to them for interacting with their humanoid robot. We also have brought the entire TACHIP integrated teleoperation system into technical EXPO, for different visitors to try this system continuously for two days. We have received valuable comments and suggestions from both normal visitors and expertise.

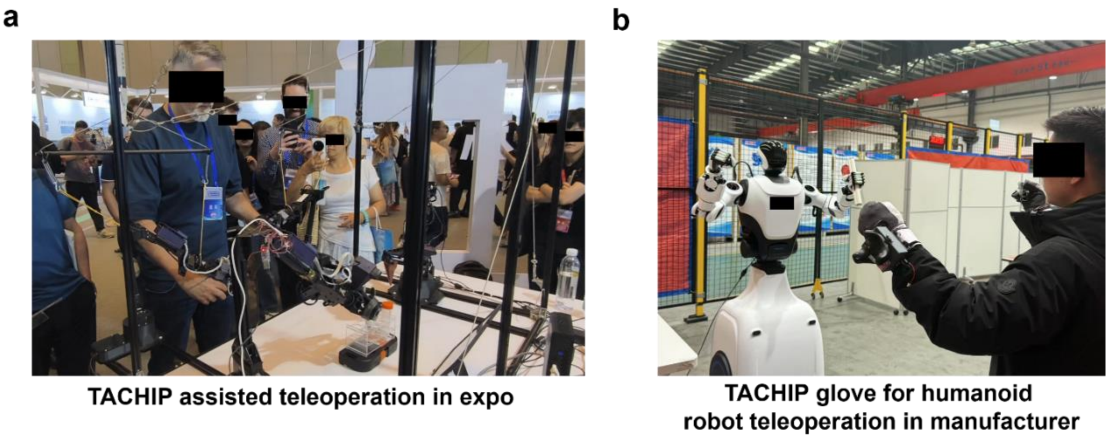

Fig. R7. Demonstrations and trials of TACHIP-enabled robotic arm and glove teleoperation system.

Overall, to enhance the scalability in more complex system, we have proposed the concepts of modular design for both TACHIP and back-end system. We also have made TACHIP as simple as possible, to

avoid low compatibility of complex device design/structure to the target system. All of materials used in TACHIP are soft, and make it completely flexible, or even rollable/foldable. It is then mechanically compatible with different platforms with various surface conditions. However, as a primary research, there are definitely some bottlenecks existed. The design of FPC and TACHIP need small customizations for better integration, or we can simple print some accessories designed for that complex system. The micro-manipulation function may not work very well if two surfaces are not flat. Although the piezo pump is quite small, massive number of TAHIPs for scalable deployment may also bring size and power consumption issues. However, compared to the power consumption of the platform which TACHIPs are intended to be integrated, our system only occupies a small portion of the entire consumption. For system-level latency, we also agree that the delay of tactile communication may cause spatiotemporal deviation of perception and lead to high risk of failure of operations. However, according to massive amount of experiments done be multiple participants, a system latency of 100-300 ms is still reported as acceptable for most of typical operations. Especially for fine teleoperation, the users were usually acting quite slowly to avoid misalignment. It is also true that pneumatic actuation usually possesses lower response time compared to other cutaneous feedback, but it also provide advantages such as softness, regeneration of shapes and stiffness, as well as simultaneous static and dynamic stimuli, etc. The system latency is also a common issue for all of those teleoperation and haptic feedback technologies, and worth for continuous study. In our opinion, there is still no perfect solution which can conquer all practical applications. We will strive to investigate the way of improving the issues mentioned by the reviewer.

Reviewer #3 (Remarks to the Author):

The manuscript reports a soft “tactile chip” (TACHIP) that co-integrates pneumatic actuation with in-situ sensing for telehaptics. Each chip comprises a five-pixel silicone/PDMS pneumatic array; the PDMS membrane embeds liquid-metal microchannels that serve dual roles—as piezoresistive pressure

sensors and as micro-heaters for thermal feedback—while a pectin thin film provides temperature sensing. A flexible printed connector enables rapid system integration. The authors claim that in-situ sensing improves spatial co-registration between actuation and measurement, enabling self-adaptive haptic rendering (e.g., “hard” vs “soft” through pressure modulation) and a quantitative alternative to purely subjective pattern tuning. They further demonstrate fusion of haptic and thermal cues, object-shape recognition when multiple chips are mounted on a gripper, and a “reverse haptic feedback” concept: finger micro-motions on the master side are projected to a slave gripper by treating the five-actuator array as a small parallel manipulator for fine teleoperation. Reported figures include forces up to 1.67 N, operation up to 500 Hz (vibrometer-measured micromotions), and first-round subjective identification rates around 95% for selected patterns; fabrication and a portable control module are described. Overall, the work targets modular, add-on telehaptic capability that couples actuation and sensing in a compact soft system, with demonstrations spanning sensing-assisted feedback design, multimodal feedback, and micro-manipulation within a teleoperation workflow.

Here are my major comments:

1) When units U1–U5 are operated as actuators, the observed variations in voltage (V) are considerably smaller than those recorded when the same elements are used as force sensors. This suggests that the externally applied forces during sensing tests may exceed the actuation forces that the system itself can generate. From a haptic-interface standpoint, the actuation and sensing ranges should ideally be comparable to ensure transparency, accuracy, and repeatability. The authors are encouraged to clarify the relative magnitudes of the applied and generated forces, and to discuss how this mismatch may affect bidirectional performance and overall system fidelity.

We thank a lot for the careful consideration and valuable comment. The reviewer mentioned a popular and common issue regarding the accuracy/consistency between the detected and the regenerated stimuli. It is indeed a very important aspect for delivering realistic information to assist human machine interaction. We would like to address this issue from two points.

First of all, it is true that the haptic feedback forces are much lower than the real loading forces in different practical applications. However, the maximum force generated by our pneumatic actuator is about 1.5N, which is a relative high force compared to other reported works (usually hundreds of mN). By considering finger perception, this is also a strong force within a constrained space (ring-shaped handler of the controller). In fact, during teleoperation tasks, too larger haptic feedback forces or displacements exerted on user’s finger may cause some troubles, such as losing control of the handler/controller. In addition, robotic operation usually contains very high contact force which is harmful to humans. The accurate haptic feedback (1:1 ratio of sensing and feedback) of those forces will definitely cause injury of user. Hence, the main purpose of haptic feedback is to replicate a general tactile information regarding static and dynamic events qualitatively. The accurate projection of

feedback forces with quantitative magnitudes is only feasible when the real contact forces are within the safe range.

Second, by considering the above issue, we conclude that the ratios of projection of sensing and feedback force are necessary for different operation tasks, such as 10:1 for heavy loaded operation, 1:1 for manipulation of small object. As long as the range of the actuation force is wide enough to present the variation of major pressure levels during operation, the user is able to identify the status by one or few trials. Overall, the relative magnitudes of the applied and generated forces become a tunable relationship of projection under the maximum actuation forces. Specifically, the ratios of the applied and generated forces are set from 5:1 to 10:1 in our experiments.

2) In the current experiments, the normal forces applied by the human operator appear uncontrolled and are not quantitatively measured. It would be more rigorous to apply a known and measurable normal force—using, for example, a calibrated load cell or force gauge—so that the actual contact force can be compared directly with the sensor outputs of the TACHIP system. This would allow a more quantitative assessment of the sensor accuracy and the fidelity of the haptic feedback.

We thank a lot for the careful inspection and valuable comment. Yes, it is a critical problem appeared in this part. Standard characterization is very important for quantitative assessment. We appreciate this comment raised by reviewer. We have performed the force measurement with force gauge simultaneously when the human operator has applied stepwise loading forces. The result shows a relative good consistency between force gauge and TACHIP. On the other hand, there are also some deviations of the measurements. The possible reasons may include the mechanical influences of air chamber and elastomer substrate, and the effective sensing region of liquid metal pressure sensors.

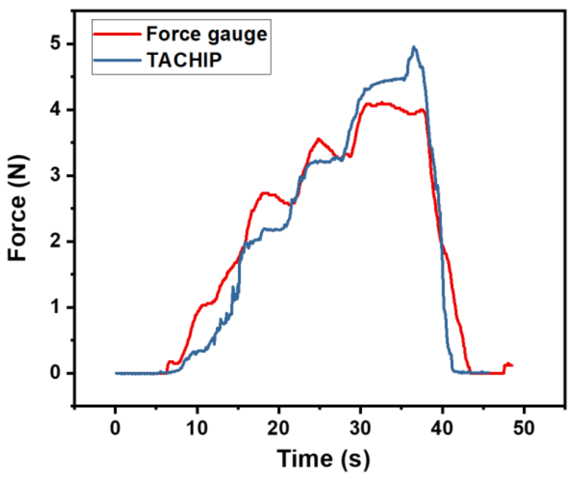

Fig. R8. Comparison of in-situ sensor and force gauge monitored tactile information.

3) In the experimental results, the baseline voltages of channels V1–V5 differ even when no normal force is applied. The authors should clarify the origin of these offsets—whether they arise from fabrication tolerances, residual stresses in the pneumatic membranes, variations in liquid-metal channel geometry, or electronic drift. Explaining or compensating for these baseline differences would improve the interpretation of the sensing data and the overall reliability of the system.

We thank a lot for the careful inspection and valuable comment. We are not sure about the exact part of the experimental results mentioned in this comment. We will try to address the reviewer's concern from two aspects.

First of all, it is true that there will be fabrication tolerances and variations in liquid-metal channel geometry. However, as we were using photolithograph fabrication process, the resulted resistance variations are very small and negligible in our applications, this also can be verified in Figure 5, which provides the signals from five channels with almost the same baseline (no offsets).

Second, if the reviewer was concerning about the signal offsets appeared in Figure 7 and the real-time signal monitoring window in demonstration videos, these are the manually added offsets to the original sensing signals. The purpose of these offsets is to make clearer data presentations of each channel, especially when there are too many channels overlapping. To avoid the possible misleading of data interpretation, we have added additional description in the caption of the figure with data offsets.

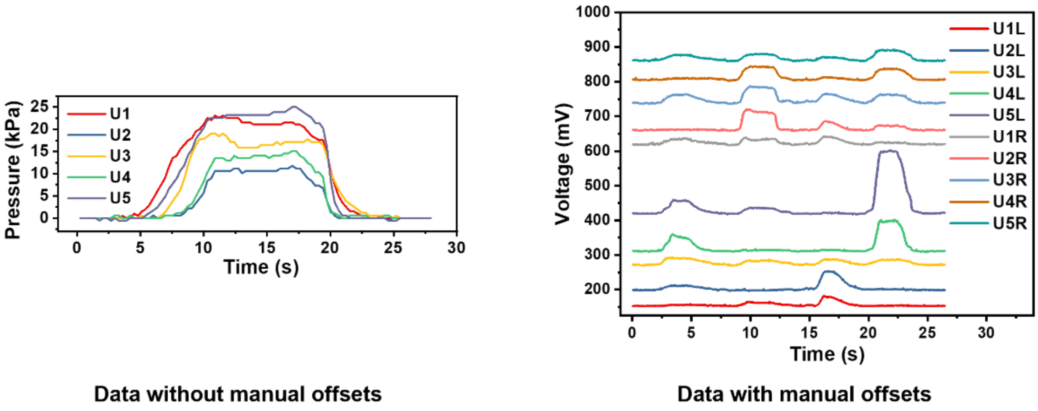

Fig. R9. Examples of sensing data without and with manual offsets.

4) In the high-frequency tests (50 Hz and 100 Hz), the supplementary video appears to be played at approximately 1.5× real speed. At such frequencies, if the video speed is altered, it should instead be slowed down to allow the physical membrane motions to be perceptible to the human eye. Otherwise, it is difficult to confirm whether the actuators truly deform at the claimed frequency. Furthermore, the rationale for placing an additional object on the actuators during these demonstrations is not explained. The authors should clarify the purpose of the added object—whether it serves as a visual marker, a mechanical load, or part of the experimental measurement

We thank a lot for the careful inspection and valuable comment. The additional object placed on the

actuator is a visual marker for verifying vibration caused by high frequency actuation. We apologize for this improper illustration. We have tried to slow down the video to check the motions at high frequency, but unfortunately our camera cannot capture such high motions for presentation. Instead, we have applied laser doppler vibrometer (LDV) to monitor both vibration frequency and amplitude in real-time. The corresponding video has been added into the original demonstration.

5) The manuscript does not provide any information on the durability or lifetime of the pneumatic actuators and integrated sensors. Have the authors performed life-cycle or fatigue tests to assess performance degradation under repeated actuation? An estimate of the operational lifetime (e.g., number of cycles before noticeable loss of sensitivity or force output) would be valuable for evaluating the device’s practical reliability and suitability for long-term teleoperation applications.

We thank a lot for the valuable comment and suggestion. It is very important to have durability test for not only sensor, but also actuator. To address issue, we have conducted additional experiments with two approaches. First, we have re-used an old TACHIP (fabricated one year ago) with continuous cyclic actuation for over 20 hours under the constant actuation parameters (50% of input power of pump, 2 seconds/cycle), and recorded the sensing data via TACHIP at different stages to evaluate variations of performance. Second, to investigate the reliability and durability of TACHIP pressure sensor, we have also applied the standard pressure meter to double check the real-time input pressure. Thus, both the robustness of pneumatic actuator and pressure sensor have been evaluated. The experimental data has been added accordingly. It can be seen that there is little degradation of general performance over time and cyclic usage. According to the experimental records, the pneumatic actuators and pressure sensors can last for millions of cycles without noticeable fatigue for normal usage. However, the excessive input pressure and contact with sharp objects may significantly affect the lift-time.

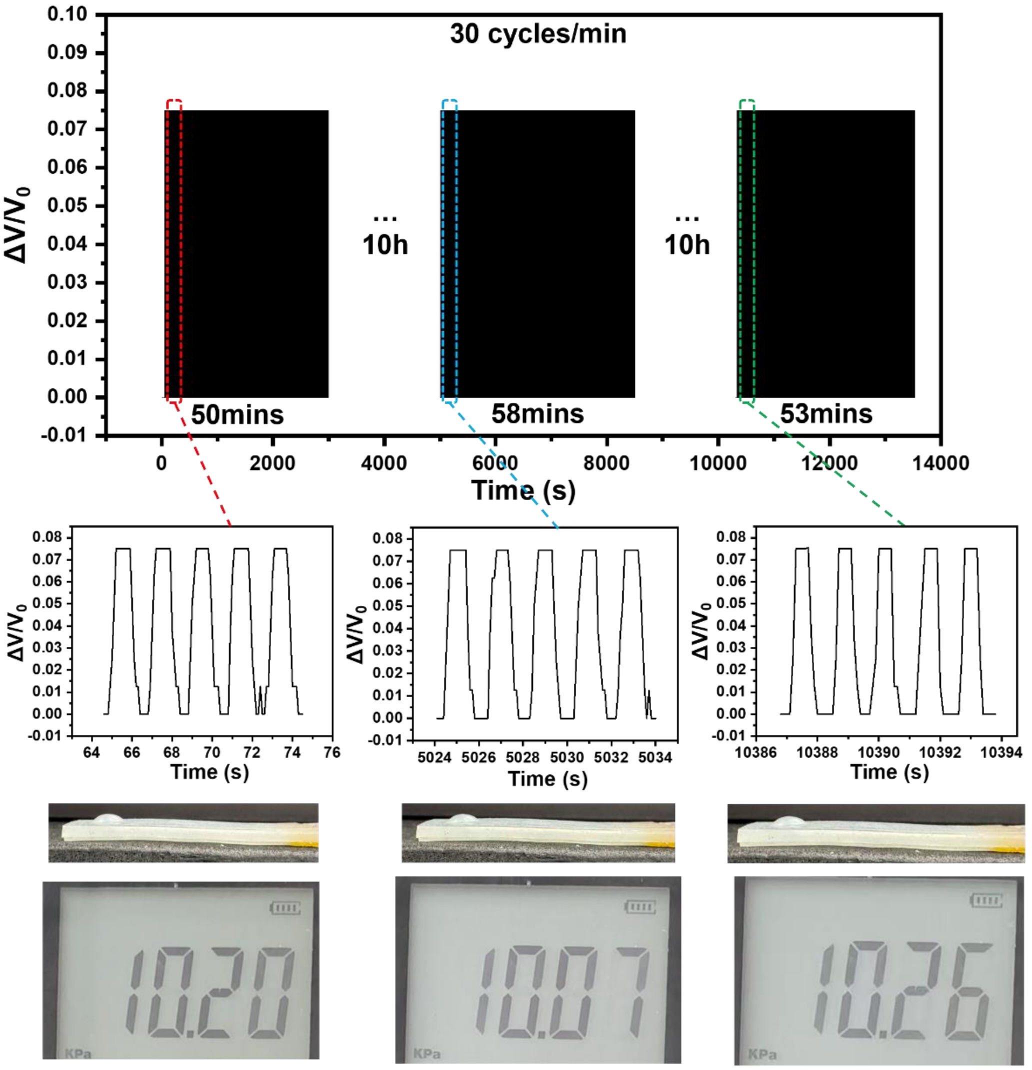

Fig. R10. Cyclic test results of pneumatic actuation and in-situ sensing within 24 hours (30 cycles/min, 50% of input power of pump). The zoom-in data of in-situ sensing, the real photos, and the manometer monitored actual pressures are shown below.

6) In the micro-manipulation experiments, the desired trajectories (triangle or square) are presented, but the corresponding actual paths followed by the system are not explicitly shown. It is therefore unclear how the authors evaluated the tracking performance or validated the accuracy of motion execution. From the provided figures, it even appears that the “desired” red path may have been derived from the recorded trajectory itself. For clarity, the desired and actual paths should be plotted together on the same graph, and the experiment should be repeated multiple times (e.g., three to five cycles) to demonstrate the repeatability and precision of the micro-manipulation.

We thank a lot for the careful inspection and valuable suggestions. Firstly, we apologize for this misleading illustration of red path. It is correct that the red path is just an expected trajectory which is

drew as a displayed item. The real paths were not shown in original figures. As can be seen in supplementary video, although we can still get the similar desired shape of trajectory, the actual paths still shown some deviations from the expected path, due to non-linear behavior and fabrication inconsistency of pneumatic actuators. As shown in figure below, we have done the cyclic trajectory tests not only for a single gripping, but also for re-gripping scenarios. Two main issues were spotted, one is the distortion of some parts in the trajectory, another one is the increasing or decreasing of the entire size compared to the desired shape. The cyclic test for a single gripping shows relative good repeatability, which also includes repeated distortion and size issues. On the other hand, the results of re-gripping tests show that the different distortions are observed, due to slight changes of gripping status of tool, and those repeated trajectories after re-gripping are also showing good consistency. As a preliminary trial on the soft haptic actuator-based manipulation, one of main purpose of this demonstration is to verify its capability of providing additional DoFs to normal gripper, without adding extra components, e.g., parallel manipulator. After the verification of its potential, we will also strive to improve the tracking performance with better precision in our future works.

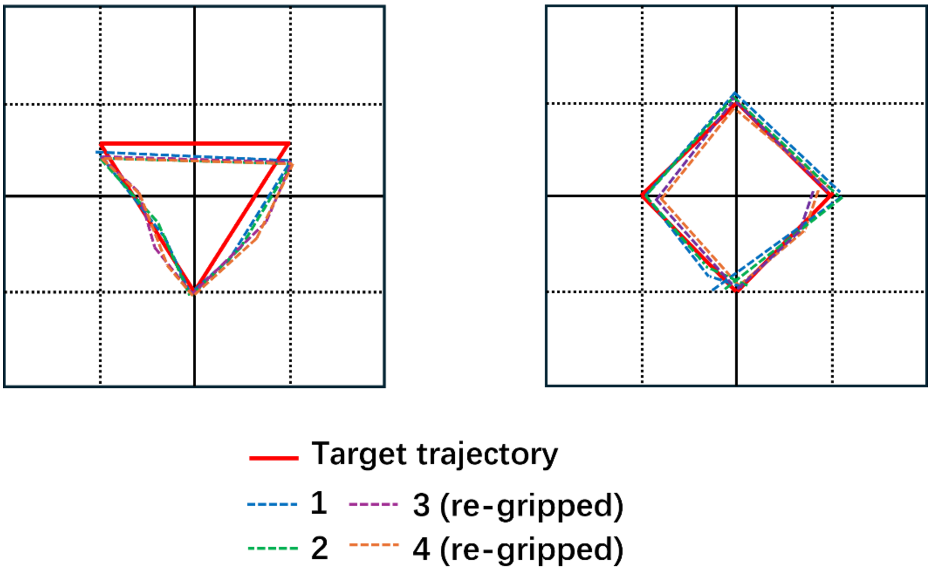

Fig. R11. Comparison of target trajectories (red line) and actual trajectories (dot line) for repeated manipulation and repeated manipulation after re-gripping.

In addition, we are more focusing on the “human in the loop” control of the micro-motion manipulator at this stage. In this case, it is more like a fine teleoperation system in which the trajectory can be simultaneously adjusted by TACHIP of controller side via observation. The user can always tuning the twisting or pressing force on the TACHIPs at the controller to make the end-point approach to the desired position.

7) The manuscript does not specify the stiffness level of the pneumatic actuators during the micro-manipulation experiments—whether they operated in a soft, medium, or hard state. Was the effective stiffness tunable during manipulation through modulation of the internal pressure, or fixed throughout the test? Since actuator stiffness directly influences positioning accuracy and repeatability, quantitative information on its value and controllability would strengthen the analysis. An explanation of how stiffness variation affects manipulation precision would also provide useful physical insight.

We thank a lot for the careful inspection and valuable suggestions. The reviewer indicates an important issue regarding the positioning accuracy for the TACHIP based micro-manipulation. It is true that the general stiffness will affect its positioning accuracy throughout the entire operation. Generally speaking, inspired by Stewart parallel manipulator, the multi-DoF micro-manipulation is achieved by tuning the heights of displacement of the corresponding pneumatic actuators, and making the grabbed object to be twisted accordingly. As the intrinsic stiffness of elastomeric material is fixed at a given operation temperature, we can only tune the general stiffness of pneumatic actuator via input pressure, and realize the twisting motions through variations of displacements. Overall, the stiffness of the pneumatic actuator is not fixed and experiences continuous tuning along with the deformation. From the physical view point, although there is non-linear behavior of deformation caused by pneumatic actuation, the simulated results indicate that the actuator is still have acceptable linearity within a specific range of pressure. The main issue of large deviation from the desired position may refer to the deformation caused by the contact force when the object is manipulated. There will be unpredictable external loading forces applied on the pneumatic actuator for operating random objects. As mentioned by reviewer in below comment as well, this additional loading force will firstly reduce the displacement calculated by simulation with little or no external loadings. and also change the input pressure required to twist the tip to a desired position. Specifically, as the mass of the object increases, larger input pressures/stiffness are required for moving the same distance. In terms of the stiffness level of pneumatic actuators in micro-manipulation, the stiffness level of the central actuator (act as pivot) remains in a hard state. The rest of four actuators (act as manipulators) keep changing their stiffness for operating the tool. In the meantime, if the desired position is reached, the greater stiffness of the supportive actuators (the actuators bearing the major loading forces) can ensure less deviation (positioning error) under larger external loading force.

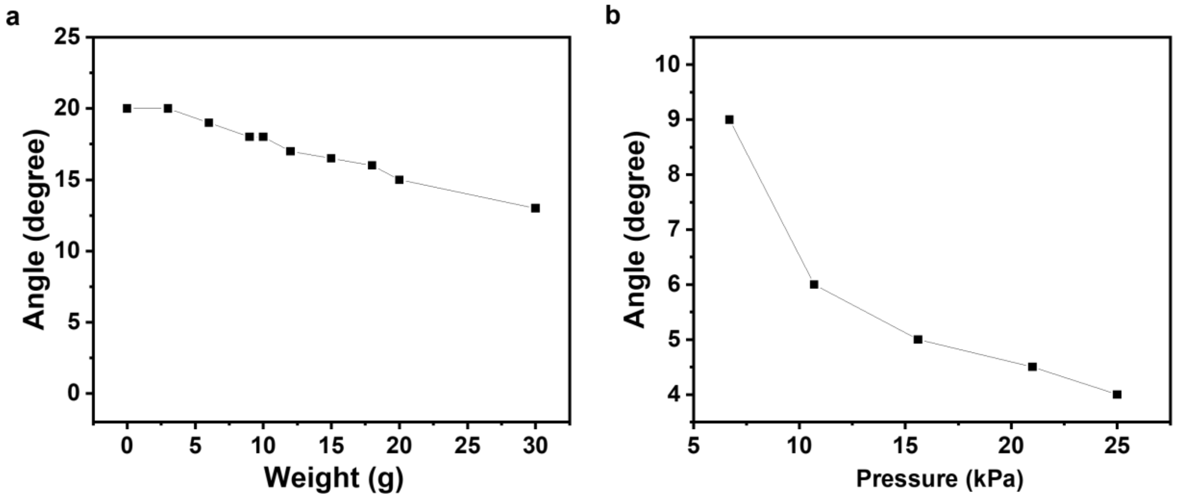

Fig. R12. a. Angle variations of the tip of the tool under the increased loading weight at the tip. b. Deflection angle of the tip of the tool under the changing input pressure (stiffness) of the actuator for a given load (30g).

As mentioned in above comment, for the mode of coordinates input based manipulation (mode 1), there are still relative large deviations from the desired position at current stage. But the general shape or trajectory formed by the program can be clearly identified. To further improve the performance, we will try to adopt close-loop control and algorithms which include more variables (e.g., deformation caused by contact force during grabbing) to increase the accuracy of positioning. On the other hand, the repeatability of positioning is relatively good, as long as the manipulated object remains unchanged. The compensated pressure is then required if the object is changed.

For the mode of reverse haptic feedback based teleoperation (mode 2), this human in the loop manipulator possesses relative good operation accuracy as user can always tuning input pressure by observation.

In fact, considering the non-linearity of elastomeric actuator, the main purpose of TACHIP-based micro-manipulator is to operate tools and objects with small weight, instead of heavy loaded tasks. For heavy loaded tasks, we may change substrate materials and pump system to improve the performance. Again, we highly appreciate this concern raised by the reviewer. Understanding and evaluation of the fundamental working mechanism is important. It is definitely the critical problems for implementing the coordinate-based manipulation with high accuracy in the practical application.

We have added the relevant discussion in the supplementary information.

8) It is unclear whether the angular trajectories shown in the micro-manipulation experiments were tracked using feedback of the actual angles or operated purely in open-loop mode. If feedback was employed, the authors should specify the sensing method and control algorithm used (e.g., PID, model-

based, or other). If no feedback was applied, it is difficult to understand how the system achieved accurate angular motion, given the nonlinear and compliant nature of pneumatic actuation. Clarifying whether a closed-loop or model-based control strategy was implemented—and providing details of its accuracy—would significantly improve the technical transparency of the work.

We thank a lot for the careful inspection and valuable suggestions. Firstly, the current demonstrated angular motion was operated in open-loop mode. The resolution of 1 degree motion was done by model-based control. Although we could achieve even smaller angular motion by current device, the model-based control cannot achieve similar resolution due to non-linearity. We also apologize for the vague description of the purpose of this experiment. Actually, the main goal of this experiment is to verify the stability and the smallest step (or resolution) can be achieved by the whole pneumatic system. At early stage, we have found that airtightness of entire pneumatic parts and performance for pump affected the general stability of pneumatic actuation. Many commercial mini-pump cannot achieve such small tunable air pressure, and also cause fluctuations or vibrations to pneumatic actuators. Thus, these problems affect the application of micro-manipulation eventually. Therefore, this experiment was primarily designed to verify the capabilities of the TACHIP based micro-manipulator, it proves (1) the stability, (2) the potential resolution, and (3) the feasibility of twisting motions for multi-DoF manipulation. The stability and the potential resolution also serve as foundation for high precision manipulation in close-loop control.

In terms of close-loop control, the key aspects of achieving high angular resolution include, (1) sensing signals from in-situ sensor or additional gyroscope, and (2) fixed shape, center of gravity and mass of grabbed object during operation. For in-situ sensor assisted close-loop control, we still need to obtain empirical equation to understand the relationship between the angle and the sensing signals from all of functional actuators, so that we can tune the pressure accordingly.

The clarification of the control strategies have been added in the method section.

9) In the presented micro-manipulation experiments, no external contact force appears to be applied to the tool, suggesting that the tests were conducted under unloaded conditions. In practical applications, however, the manipulator would interact with external objects and thus experience contact forces that could influence accuracy and stability. The authors should verify how such external loading would affect the system's performance, quantify the expected positioning error under realistic contact forces, and discuss the implications for real-world teleoperation or fine-manipulation scenarios.

We thank a lot for the careful inspection and valuable suggestions. As mentioned earlier, by considering the non-linearity of elastomeric actuator, the main purpose of TACHIP-based micro-manipulator is to operate tools and objects with small weight, instead of heavy loaded tasks. For heavy loaded tasks, we may change substrate materials and pump system to improve performance. Furthermore, based the concept of teleoperation in real-world, micro-manipulation is mainly done by human in the loop control. The user can also adjust the applied forces to compensate the external loading induced

deflection of the tip.

To quantify the expected positioning error, we have done extra experiments to evaluate the effect of loading force induced variation of tip positioning. Specifically, we have tested the influences of the increasing loading mass to the tip of the tool which has been pre-tilted by 20 degrees.

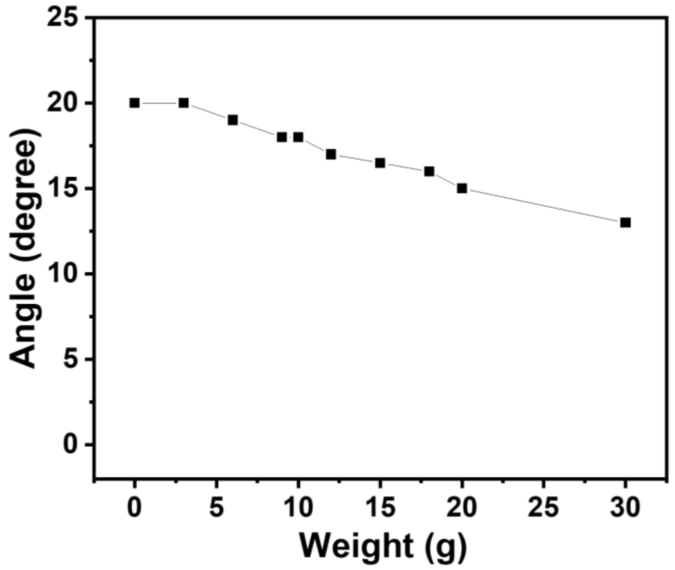

Fig. R13. Angle variations of the tip of the tool under the increased loading weight at the tip.

10) The graph of $\Delta V/V_0$ versus pressure in Fig. 2c provides useful information on sensor performance. However, the origin of the plotted data is unclear. Were these curves obtained from the authors' own fabricated sensors with different design parameters, or are some taken from previously published studies for comparison? Clarifying the source of each dataset and specifying whether all samples were designed and characterized within this work would greatly improve the transparency and credibility of the results.

We thank a lot for the careful inspection and valuable comments. We apologize for the misleading label of the legend. All of those data curves were obtained by us at the early stage of designing our liquid metal channels, the key parameters were compared based on the sensing performance. The best geometric parameters were labelled as "this work" for fabricating the final TACHIPs. We have revised the labels to clarify this issue.

11) In Fig. 4b, the description of the experiment is unclear. The authors state that "the experimental data for detecting the increasing air pressure and the cyclic inflation are shown," but it is not specified which variable is controlled and which is measured. From the plots, it appears that the upper panel

corresponds to stepwise increases in input air pressure (10–20 kPa) to demonstrate sensitivity, while the lower panel shows cyclic inflation–deflation at a fixed pressure (16 kPa) to verify repeatability. This clarification should be explicitly stated in the text or figure caption. The authors should also mention how the pressure was applied (e.g., via piezo pump or external regulator) and whether $\Delta V/V_0$ represents voltage or resistance change from the liquid-metal sensor. Clear identification of the independent (input pressure or actuation cycle) and dependent (sensor output) variables would greatly improve reproducibility and reader understanding.

We thank a lot for the careful inspection and important suggestion regarding the testing standard. The entire test setup consists of a TACHIP with in-situ sensor, a commercial air pressure meter as reference, a piezo pump controlled by microprocessor, and a three outlets air tube connecting all three components above.

For the actual testing procedures, the in-situ monitoring of stepwise increases of input air pressure was conducted by increasing the supplied air pressure from piezo pump (by controlling the input power of piezo pump through PWM port). And the real input pressures were also recorded via commercial air pressure meter. Therefore, this experiment result indicates the sensitivity of in-situ sensor.

The in-situ monitoring of cyclic inflation was performed by cyclically controlling the input air with a fixed pressure (16kPa) and fixed frequency (~8 seconds per cycle). This experiment shows a relative good repeatability via the consistent sensing output, which was also verified by air pressure meter.

As the piezoresistive sensor requires power supply to operate, and the analog input is actually acquiring the voltage across the resistive sensor when the resistance is altered under external stimuli. The $\Delta V/V_0$ actually represent resistance change from the liquid-metal sensor.

12) The authors report a response time of approximately 49 ms for the in-situ pressure sensor (Fig. 2d), which corresponds to a bandwidth below roughly 20 Hz. However, later sections (Fig. 4d(ii–iii)) claim pneumatic actuation and vibrometer-measured displacements up to 100–500 Hz. It is unclear how the soft pneumatic pixels—with their compliant membranes and air chambers—can deform and be monitored effectively at such high frequencies when the integrated sensor cannot follow signals faster than about 20 Hz. The authors should clarify whether the 100–500 Hz values refer to the pump drive frequency or the actual membrane motion, provide a frequency-response curve (amplitude and phase or −3 dB bandwidth) of the pixel under defined conditions, and explain whether the 49 ms response was measured under similar loading or if a faster sensing pathway exists for high-frequency operation. Reconciling the sensor dynamics with the claimed actuation bandwidth is necessary to substantiate that the TACHIP can operate reliably in the 100–500 Hz regime.

We thank a lot for the careful inspection and valuable comments. We totally agree that the difference between the drive frequency and the actual vibrational motions need to be clarified. We would like to address this comment from sensor and actuator aspects.

Firstly, in-situ sensor made by encapsulated liquid metal in actuation membrane is a typical piezoresistive sensor. It is correct that the response time is around 40~100 ms, which is suitable for monitoring tactile stimulus or pneumatic actuations below 20 Hz. It can cover the most situations during teleoperation tasks.

Secondly, owing to the high operation frequency of piezoelectric pump, the 98-475 Hz is the frequency range of the actual membrane motions generated by the target drive frequencies range from 100 Hz to 500 Hz, and the corresponding data curves were measured by Laser Doppler Vibrometer (LDV) pointing at the center of a pneumatic actuator. To further prove the experimental results, we have modified the supplementary video 1 and added the real-time data display from LDV. The real-time displacements and the frequency response curves are all shown in the data display window of LDV. According to the measured base frequencies and the corresponding displacements, the TACHIP is capable of operating reliably in the 98-475 Hz regime, with the errors of about ±1~5% compared to the target frequency. It is worth mention that, although there is a small deviation between the drive frequency and the actual vibrational motions, the measured frequency during the actual motion remains quite stable and reliable, with a fluctuation of less than ±0.2 %. In addition, we also have tested the displacements at the actual motion frequency of about 800Hz which is almost the threshold of the human perceivable vibration. It still can generate a peak-peak displacement of about 0.5 µm, and is still above Pacinian threshold (~0.15µm) of displacement perception. However, this type of high frequency vibration is rarely needed in most cases, unless there is a requirement of replicating specific stimulations. Overall, as a clarification, we have replaced the labels of the data curves by the actual measured frequencies.

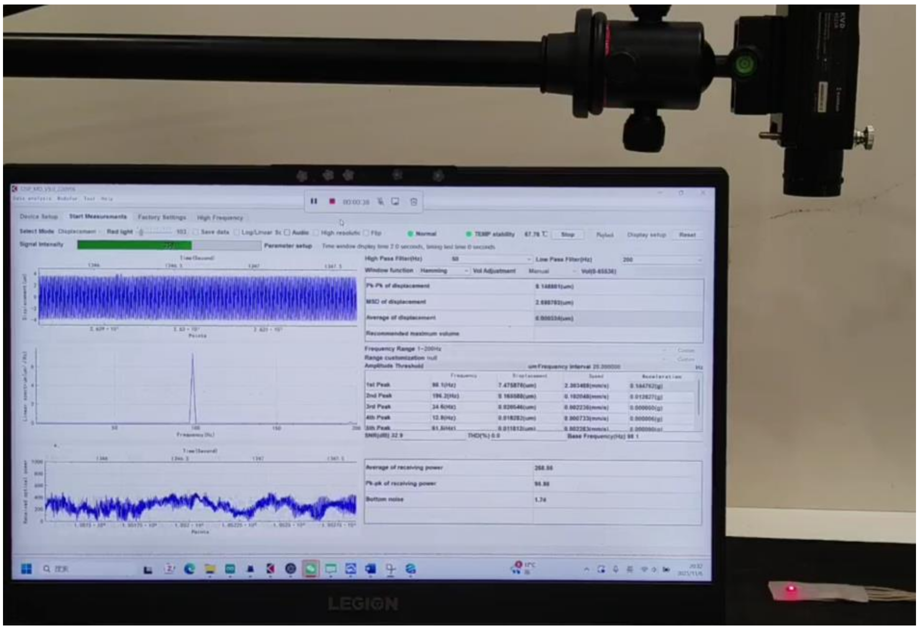

Fig. R14. Photo of test setup of using Laser Doppler Vibrometer to measure frequency and displacement of TACHIP under varied actuation frequency.

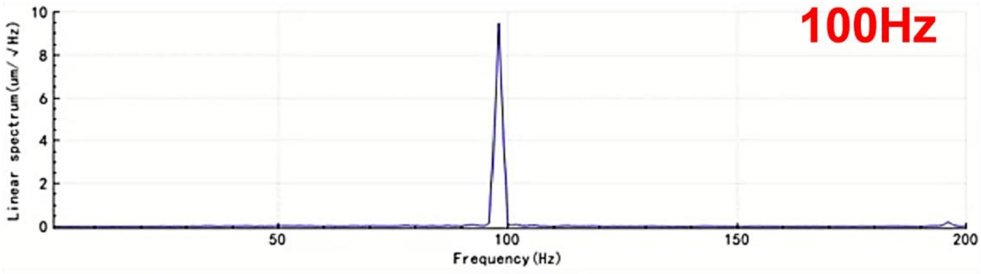

	Frequency	Displacement	Speed	Acceleration
1st Peak	98.1(Hz)	9.545380(um)	2.941333(mm/s)	0.184858(g)
2nd Peak	196.2(Hz)	0.196874(um)	0.121371(mm/s)	0.015261(g)
3rd Peak	77.7(Hz)	0.061163(um)	0.014927(mm/s)	0.000743(g)
4th Peak	54.6(Hz)	0.052279(um)	0.008970(mm/s)	0.000314(g)
5th Peak	34.4(Hz)	0.032354(um)	0.003501(mm/s)	0.000077(g)

SNR(dB) 32.7 THD(%) 0.0 Base Frequency(Hz) 98.1

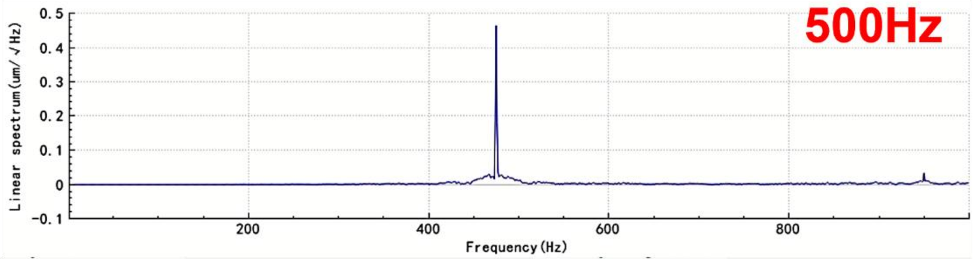

	Frequency	Displacement	Speed	Acceler
1st Peak	474.9(Hz)	0.456424(um)	0.681024(mm/s)	0.207253(g)
2nd Peak	950.0(Hz)	0.033228(um)	0.099167(mm/s)	0.060363(g)
3rd Peak	455.2(Hz)	0.030427(um)	0.043514(mm/s)	0.012692(g)
4th Peak	497.2(Hz)	0.025204(um)	0.039370(mm/s)	0.012543(g)
5th Peak	522.7(Hz)	0.014481(um)	0.023778(mm/s)	0.007963(g)

SNR(dB) 17.2 THD(%) 1.3 Base Frequency(Hz) 474.9

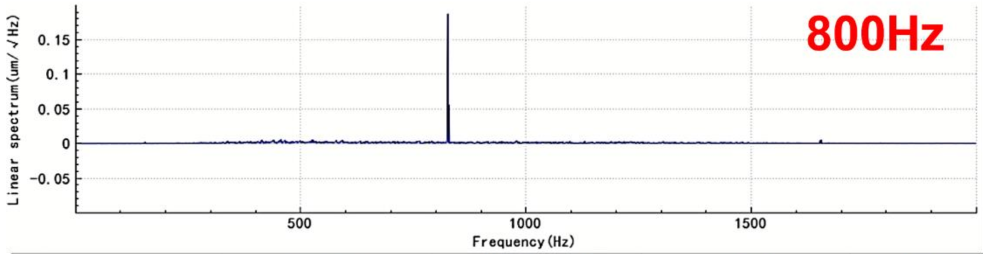

	Frequency	Displacement	Speed	Accelerati
1st Peak	826.7(Hz)	0.192943(um)	0.501082(mm/s)	0.265419(g)
2nd Peak	412.7(Hz)	0.007943(um)	0.010298(mm/s)	0.002723(g)
3rd Peak	525.4(Hz)	0.007893(um)	0.013028(mm/s)	0.004386(g)
4th Peak	455.7(Hz)	0.007495(um)	0.010729(mm/s)	0.003133(g)
5th Peak	591.3(Hz)	0.007302(um)	0.013564(mm/s)	0.005139(g)

SNR(dB) 16.1 THD(%) 0.0 Base Frequency(Hz) 826.7

Fig. R15. The experimental results of frequencies and displacements measured by LDV under different actuation frequencies.

By concerning the in-situ sensing of fast actuation frequencies, we have not implemented soft sensor with high dynamic response yet. The possible solution is to embed piezoelectric thin film into the membrane, and utilize self-generated pulse-like piezoelectric output to obtain high frequency data. We

have done the extra experiments with PVDF piezoelectric thin film attached onto TACHIP, and obtained vibrational signals with good quality at high frequencies. Similarly, by counting the peak number of the piezoelectric outputs within 0.1 second, it is also clearly showing that the TACHIP is capable of operating reliably in the 98-475 Hz regime. By cross checking the vibration frequencies measured by LDV and piezoelectric thin films with the same drive frequency, it proves that the actual frequency generated by a given actuation program shows good consistency and repeatability.

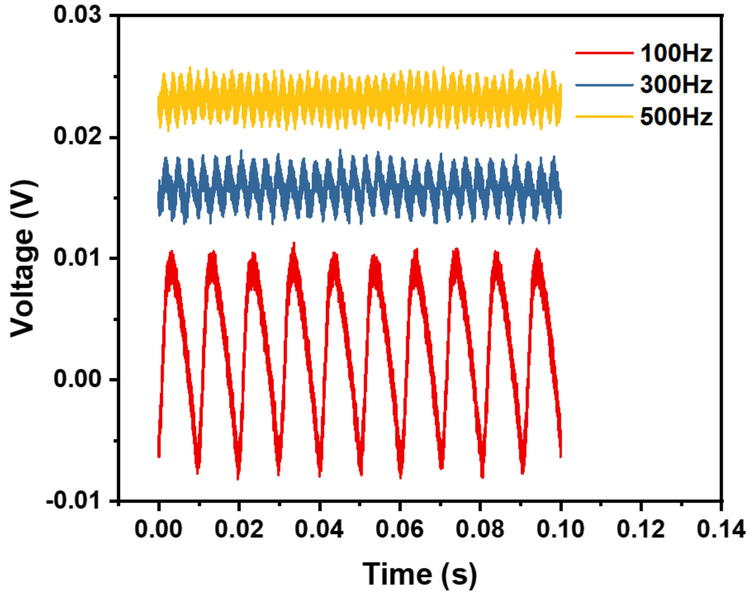

Fig. R16. The experimental results of frequencies measured by piezoelectric thin film under different actuation frequencies.

13) In Fig. 7c(ii), the fitted linear relationship y=1.16+0.63xy = 1.16 + 0.63xy=1.16+0.63x appears to match the experimental data more closely than the simulation results. However, this fit implies a non-zero displacement (y = 1.16) at zero input pressure (x = 0), which is physically inconsistent. Displacement should vanish in the absence of applied pressure. The authors should clarify whether this offset arises from measurement noise, initial membrane pre-strain, or fitting artifacts, and consider constraining the intercept to zero in the regression. Providing an explanation or corrected fit would improve the physical validity of the data interpretation.

We thank a lot for the careful inspection and valuable comment. We performed the experiment with the variables derived from theoretical simulation, and forgot to include the zero point data for linear fitting. We have replotted the data with the modified linear fitting. The updated linear fitting has a relationship of y=0.75x, as the zero point data was added.

Some minor comments:

• The term "fine tuning" should be used consistently throughout the manuscript; please replace all occurrences of "fine-tuning" with "fine tuning."

We thank a lot for the careful inspection. The corresponding parts have been corrected.

• In the Abstract, change "remains challenge" to "remains a challenge."

We thank a lot for the careful inspection. The corresponding part has been corrected.

• Define PDMS (polydimethylsiloxane) upon its first appearance in the Abstract.

We thank a lot for the careful inspection. The corresponding part has been corrected.

• The sentence "to increase the response time of thermal feedback" is misleading, as higher power would in fact reduce (shorten) the response time. Please revise to reflect the intended meaning.

We thank a lot for the careful inspection and apologize for the misleading description. It is absolutely correct that the higher power will reduce the response time. The intended meaning should be "to improve the response performance of thermal feedback" or "to reduce the response time". The corresponding part has been corrected.

• In Fig. 5a(i), the text refers to Px, tx, and fx, but these parameters are not visible or labeled in the figure. Please ensure that all referenced quantities are explicitly indicated and described.

We thank a lot for the careful inspection and valuable comment. All of the parameters have been labeled in the figure and described in the main manuscript accordingly. Here are the detailed descriptions

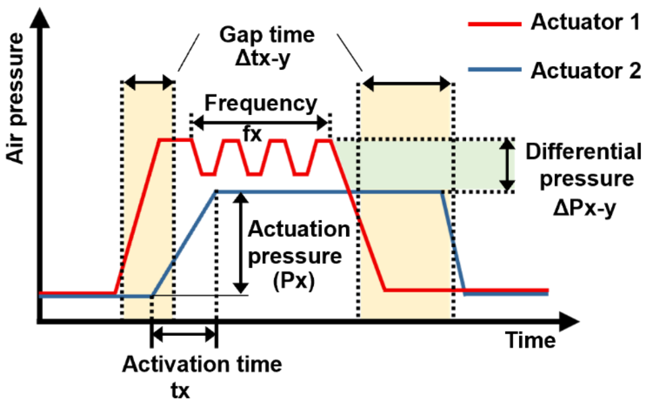

Fig. R17. Schematics of parameters used for quantifying haptic feedback pattern.

- Actuation pressure (Px, x is the index of actuator): the input pressure of actuating an actuator, and is directly related to displacement and hardness.
- Differential pressure (\Delta Px-y, x and y are the indexes of actuators): the differences of input pressures between the adjacent actuators. It affects identification of displacement variations between two actuators, in another word, the perception of shape/geometry/contour.
- Inflation time (tx): time of inflation of actuator x. It affects effective stimulation time.
- Gap time (\Delta tx-y): gap time between the deactivation of actuator x and the activation of actuator y. It affects the identification of temporal information between two adjacent actuators.
- Frequency (fx): actuation frequency of actuator x.

The corresponding figure has been modified with the labelled parameters.

• The statement "Two center pneumatic actuators act as support pivots for upper and lower surfaces" requires clarification. What are the stiffness values of the central versus surrounding actuators? If the central actuators function as pivots, they should exhibit greater stiffness; please explain how this condition is achieved.

We thank a lot for the careful inspection and valuable comment. It is a very important issue of realizing stable operation in micro-manipulation. We would like to address this comment from two aspects.

First, it is necessary to clarify that the central actuators do not need to exhibit very high stiffness compared to the surrounding actuators, unless the operated tools are heavy. The central actuator can work as pivot, as long as the stiffness is enough to provide the supporting and resisting force against the forces of gripping, gravity, and twisting induced by the surrounding actuators.

Secondly, we have mainly adopted two methods to modify the central actuators. (1) Applying elastomer materials with higher Young's modulus to fabricate the central actuator individually. (2) Fabricating a single elastomer pillar as a pivot accessory which can be easily bonded onto the membrane of the central actuator. In this demonstration, we have used method 2 to achieve the pivot assisted micro-manipulation. The corresponding descriptions have been added into the method section.

Table R2. Stiffness/hardness of actuator under different situations.

	Shore A hardness
Soft (Surrounding)	6 HA
Medium (Surrounding)	10 HA
Hard (Surrounding)	14 HA

Central pivot	24 HA

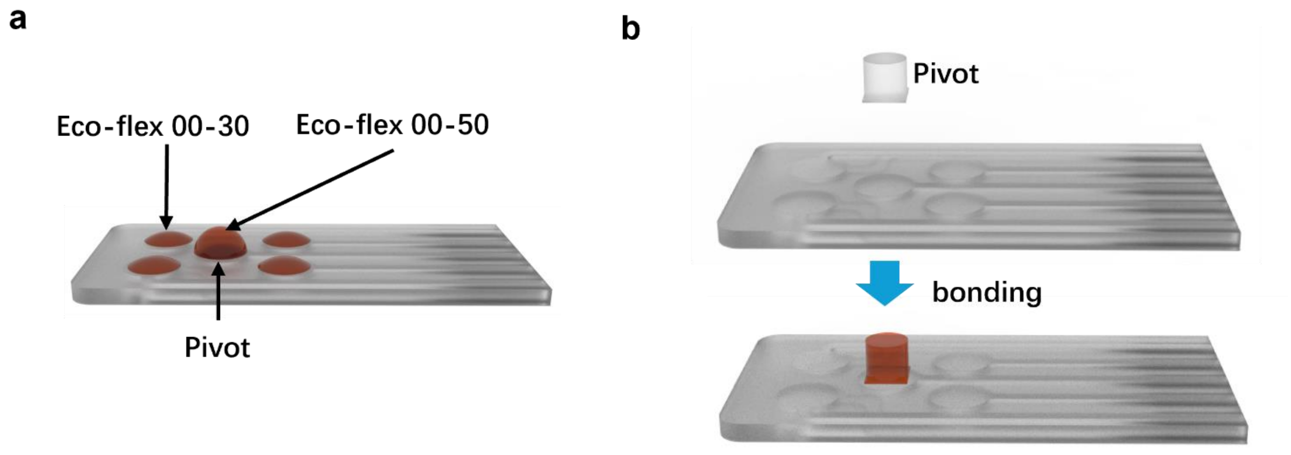

Fig. R18. Schematics of modifying the stiffness of the central actuator with two methods.

• When forces are applied to the five pneumatic pixels, a voltage around 700 is recorded. Please specify the unit or scale of this voltage (e.g., mV) in the corresponding axis label.

We thank a lot for the careful inspection and valuable comment. The unit is mV for all of the data recorded by MCU.

Reviewer #4 (Remarks to the Author):

This manuscript represents a soft tactile chip (TACHIP) made by in-situ fabrication of sensors and actuators. This chip has both haptic and thermal sensing with high-resolution haptic rendering, equipped with a flexible print circuit. Applying TACHIPs to a teleoperation system can achieve direct haptic rendering. Thanks to TACHIPs, finger micro motions from the primary side can be directly projected into the secondary side. The proposed approach realizes mutual micro manipulation with tactile perception in teleoperation, social network, and virtual training.

This manuscript provided much technical progress, but this reviewer found little scientific contribution in this manuscript.

- What are the noteworthy results?

This manuscript describes many topics, including a soft tactile chip (TACHIP), array-based haptic rendering, haptic and thermal feedback, and micro-motion projection. Thus, this reviewer feels this manuscript is unfocused, and would suggest that the authors focus on scientific contributions.

"Here, we report a soft tactile chip (TACHIP) made by in-situ fabrication of thin film sensors in the membranes of pneumatic actuator (Fig. 1a). This chip is fabricated by flexible pneumatic actuators, featured by bottom polydimethylsiloxane (PDMS) actuation membrane (thickness: 0.3 mm) with encapsulated liquid metal micro channels, and top layer with pectin-based temperature sensing film (thickness: 0.1mm)."

This is a significant technological achievement based on existing methods. This reviewer wonders what novel scientific contributions are in this manuscript. Technological achievement would be transferred to an engineering journal such as IEEE MEMS or Sensors & Actuators.

We thank a lot for the valuable comment and apology for the unclear description regarding scientific contributions. To address this issue, we have made a considerable number of modifications to the entire manuscript and the figures. The topics with less scientific contributions have been moved to supplementary information. We also have conducted further exploration of SA and FA perception regeneration via TACHIP, as per suggested by reviewer.

Overall, the revised figures and main manuscript mainly focus on two scientific contributions:

(1) In-situ sensing enhanced haptic rendering and facilitated haptic pattern programming (gross stage, Fig. 4 and 5). The enhanced haptic rendering refers to several aspects, including self-adaptive hardness regeneration via tunable SA perception by in-situ sensing, pressure and frequency modulation for replicating shape/texture/dynamic motion through fusion of SA and FA perception, etc. The facilitated

haptic pattern programming shows a methodology to quantify those perceptions for continuous improving of actuation coding of SA and FA related stimuli.

(2) Reverse haptic feedback enabled multi-DOF micro-manipulation (fine stage, Fig. 6). Differ from bilateral feedback, we adopted asynchronous revers haptic feedback, together with the array design of TACHIP, to realize a parallel manipulator inspired multi-DOF micro-manipulation which can be teleoperated by hand's intuitive micro-motion. Without modifying classic robotic gripper with expensive multi-DOF actuators, TACHIP as a quick add-on component, can be deployed to provide multi-DOF manipulation to the "last centimeter" of teleoperation.

We have rearranged the remaining figures, together with new data, to make the topics more focused. Specifically, Fig. 1 gives a general schematic of the main contribution and application of TACHIP. Fig.2 and 3 give the basic characterizations of sensing and feedback system. Fig. 4 and 5 shows the first major contribution (in charge of gross teleoperation stage), SA, FA, and in-situ sensing enabled multi-modal haptic feedback, and a corresponding new methodology to quantitatively evaluate those feedback patterns with high efficiency. Fig.6 shows the second major contribution (in charge of fine teleoperation stage), reverse haptic feedback enabled micro manipulation for fine tuning of tools. Fig 7 and 8 provide integrated demonstration and evaluation.

The authors claim that

"In addition, instead of traditional subjective test, a new methodology of evaluating haptic feedback performance with quantitative and objective method is proposed, TACHIPs with in-situ sensing allows the establishment of haptic feedback sensing database to facilitate the design of feedback pattern through data assisted pre-adjustment."

and

"Owing to the in-situ pressure sensor, as an novel methodology in establishing a more quantitative and objective evaluation standard, the sensing data can be simultaneously recorded for building database during the entire actuation process."

This reviewer wonders what "a new methodology of evaluating haptic feedback performance" implies over traditional tests. With in-situ sensors or external sensors, sensing data is available. Why do in-situ sensors alone enable building the database while external sensors do not?

We thank a lot for the valuable comment and apology for the unclear description regarding this contribution.

The fundamental issue about the performance of the haptic feedback devices and the corresponding feedback programs is the actual stimuli perceived by users. However, skin perception is a very subjective aspect which may vary among different ages, genders, or even skin conditions. It is true that

the external sensors can obtain sensing data, and it is the most traditional way to characterize the haptic feedback devices. But there are several issues which can cause deviation between sensing data and user's perception.

1. A typical approach of testing haptic feedback device through external sensors is to conduct this experiment off the body, and on a standard lab table. After the data is collected, the user is then wearing or using the haptic feedback device and filling subjective experiment questionnaires. However, due to the shape irregularity of human body or attachable platform, there are quite a lot of inconsistencies of the contact status of those actuators between two scenarios. In another word, the external sensing data does not possess a good correlation to the actual perceptions.
2. To solve this issue, a primary trial is to use soft film-based external sensing array, and attached it to haptic feedback device. Users (or attachable platforms) can then wear both external sensor and haptic feedback device together to achieve simultaneous data monitoring. However, firstly, the attached external sensor itself may reduce the haptic stimuli perceived by user, due to its material. Secondly, there may be displacement between sensor and actuator during test, and cause mismatch among those pixels, and lead to false data.

On the other hand, for haptic feedback programming, the database is considered as useful only when it is approved by human subjective tests, as the main purpose of haptic feedback is to regenerate the perception to user. It means that each pattern's sensing data should be verified by humans. For traditional test mentioned in issue #1 above, a single data collection of a specific haptic feedback pattern requires at least two steps: table test for sensing data, and human subjective test as verification. In contrast, it only takes one round of actuation (one step) to get both subjective perception and sensing data with in-situ sensor when using. As the establishment of databases usually requires millions of tests, according to numbers of patterns, different user ages/genders, etc., the time consumption can be reduced significantly (at least half) via our method. In the future, we may also investigate this database to help understand diversities of different people reacting to different stimuli, to study heterogeneous design of haptic feedback device with more comprehensive/realistic perception.

It is also worth noting that this methodology is not solely serving for our own device. It can be applied to other soft cutaneous haptic feedback techniques mentioned in introduction part, such as DEA, electromagnetic vibrator, electroosmotic actuator, fluidic actuator, etc., as long as there is soft film/membrane which can deform along with actuator. Currently, most of the haptic feedback research focus on the scientific contribution in mechanism and hardware, in order to generate different stimuli with varied pixel densities or intensities. However, fewer research is trying to investigate the possible method to utilize those as-fabricated haptic feedback devices properly, i.e., the way of developing better haptic feedback patterns efficiently. As a technology which heavily relies on personal subjective experiences, both hardware advancement and design methodology of feedback programs are sharing equal importance.

Discussion seems a summary of the proposed approach. More informative discussion is required.

We thank a lot for the valuable comment and suggestion. We have revised the discussion part with more informative contents.

The authors claim that

"The enlarged data in Fig. 4d(iii) indicates that the vibrational pneumatic haptic feedback at 500 Hz can generate 1.5 \micro m displacement which is still perceivable, according to the Pacinian threshold of humans.

and

"Fig. 4e(ii) illustrates the modality control of pneumatic array done by modulating cycling frequency and input power via program. Thus, reconstruction of SA and FA perception is achieved without additional actuators."

These could be contributions. Exploring these topics in depth would be worthy of scientific contributions.

We thank a lot for the valuable comment and suggestion. We have explored the capabilities of reconstructing SA and FA perceptions via our TACHIP, and conducted several experiments to demonstrate SA+FA perception enabled regeneration of more realistic stimulation.

To further explore the capability of TACHIP in replicating the physical properties of different materials through SA and FA stimuli, we have done extra experiments. Firstly, we have collected the tactile information by using commercial piezoresistive (SA) and piezoelectric (FA) sensors to scan across the objects, and build the reference database. Secondly, we have analyzed the frequency and the amplitude of the sensing data, and programmed the code of driving the pneumatic pump accordingly. Moreover, to verify the regenerated stimuli from TACHIP, the same SA and FA sensors were applied on the top of TACHIP, to collect the replicated “tactile information”, as can be seen in figure below.

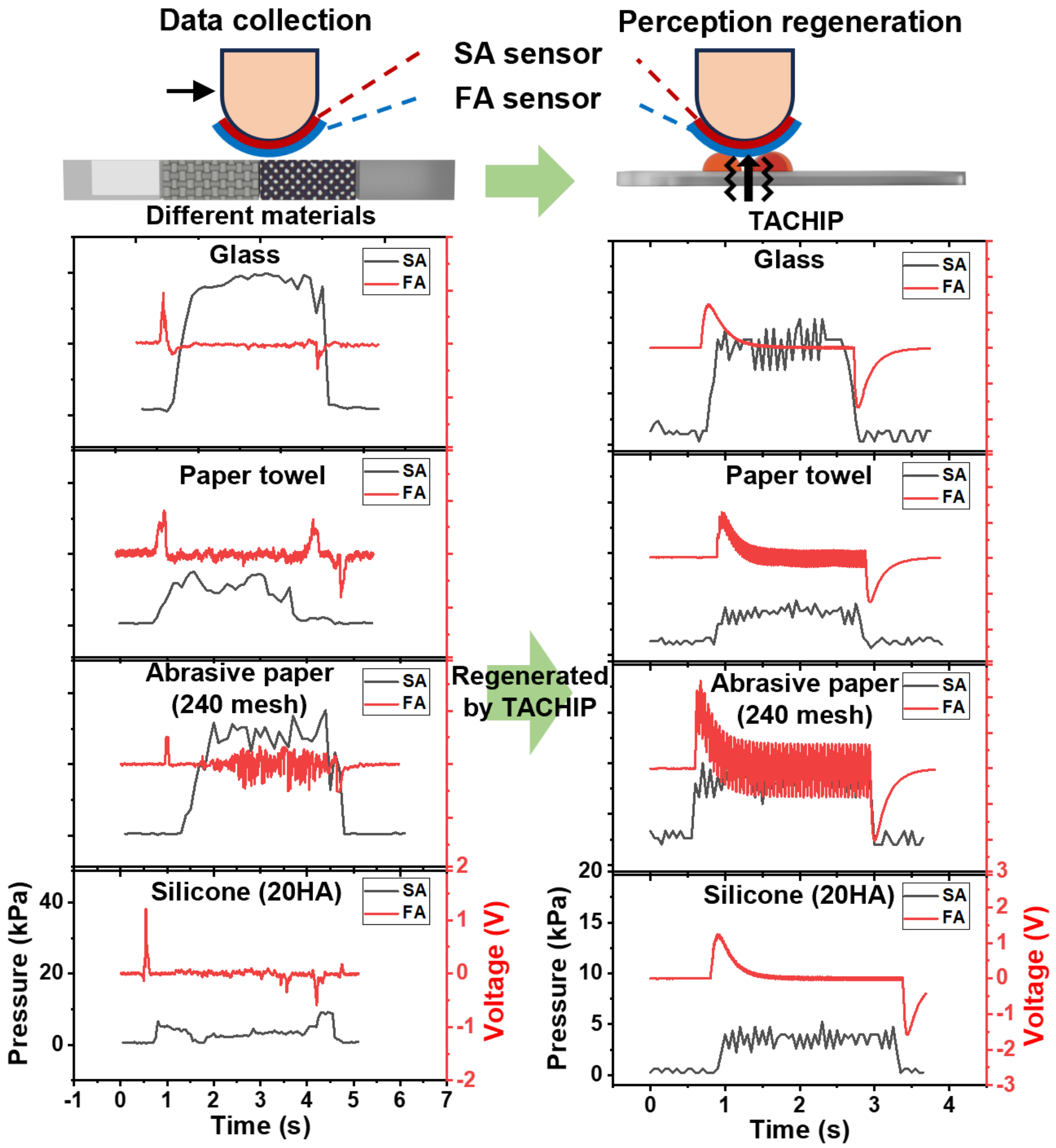

Fig. R19. Perception regeneration of different materials, including glass, paper towel, abrasive paper (240 mesh), and silicone (20 HA), (i) data collection of SA and FA sensing signals through sliding based scanning of commercial piezoresistive (SA) and piezoelectric (FA) sensor, (ii) TACHIP regenerated mechanical stimuli monitored by SA and FA sensor.

Terminology "master-slate" should be replaced by "primary/secondary", "primary/replica", or "leader/follower".

We thank a lot for the valuable suggestion. In fact, we borrowed this term ”master-slave” from the original developer (research group from Standford University) of this open-sourced teleoperation platform. It is also a commonly used terminology in teleoperation field (e.g., doi:10.1109/TOH.2009.4; 10.1109/RBME.2012.2236311; 10.1109/TRO.2011.2134130). We realized that the suggested terms provided by the reviewer are more suitable in this case. Therefore, we have replaced it by “leader/follower”.

- Will the work be of significance to the field and related fields? How does it compare to the established literature? If the work is not original, please provide relevant references.

This reviewer wonders if "reverse feedback" is coincident with "bilateral feedback", which has been proposed over the past four decades in teleoperation field. Also, "in-situ sensing" seems similar to the concept of "collocation" of sensors and actuators

We thank a lot for the valuable comment and concern about these two specific concepts mentioned in our manuscript.

For “reverse haptic feedback” issue, we also knew about “bilateral or bidirectional feedback” techniques, and also used this term in our previous works. The robotic arms of the teleoperation platform in this manuscript also possess such functions, as a well-known feature for human machine interaction. In most cases, bilateral feedback is frequently used in kinesthetic haptic feedback, such as teleoperation system, rehabilitation exoskeleton, etc., to synchronize position, torque, force, etc. For cutaneous haptic feedback, we applied “bilateral or bidirectional feedback” for the application when we need to exchange tactile information simultaneously at both sides. However, it is not the case in this manuscript. Terminology “reverse haptic feedback” comes with “direction haptic feedback”, which is the conventional transmission path of tactile information (from robot/virtual world to user). But “reverse haptic feedback” does not happen with “direction haptic feedback” at the same time. Although the tactile information is projected from user to robot during reverse haptic feedback, the purpose is not for regenerating tactile stimuli to robot obviously (it is meaningless to robot, by far). The main goal is to offer multi DOF manipulators to robot, and thus, it naturally cannot be simply considered as bilateral feedback. To conclude, first, direct and reverse haptic feedback do not work at the same time (asynchronous). Second, reverse haptic feedback actually serve as a teleoperation-like manipulation, not as a “reversed” feedback. Thirdly, it also requires the specific design of TACHIP to fulfill the purpose of manipulation, otherwise, it is just a simple reversed feedback, which is useless to robot.

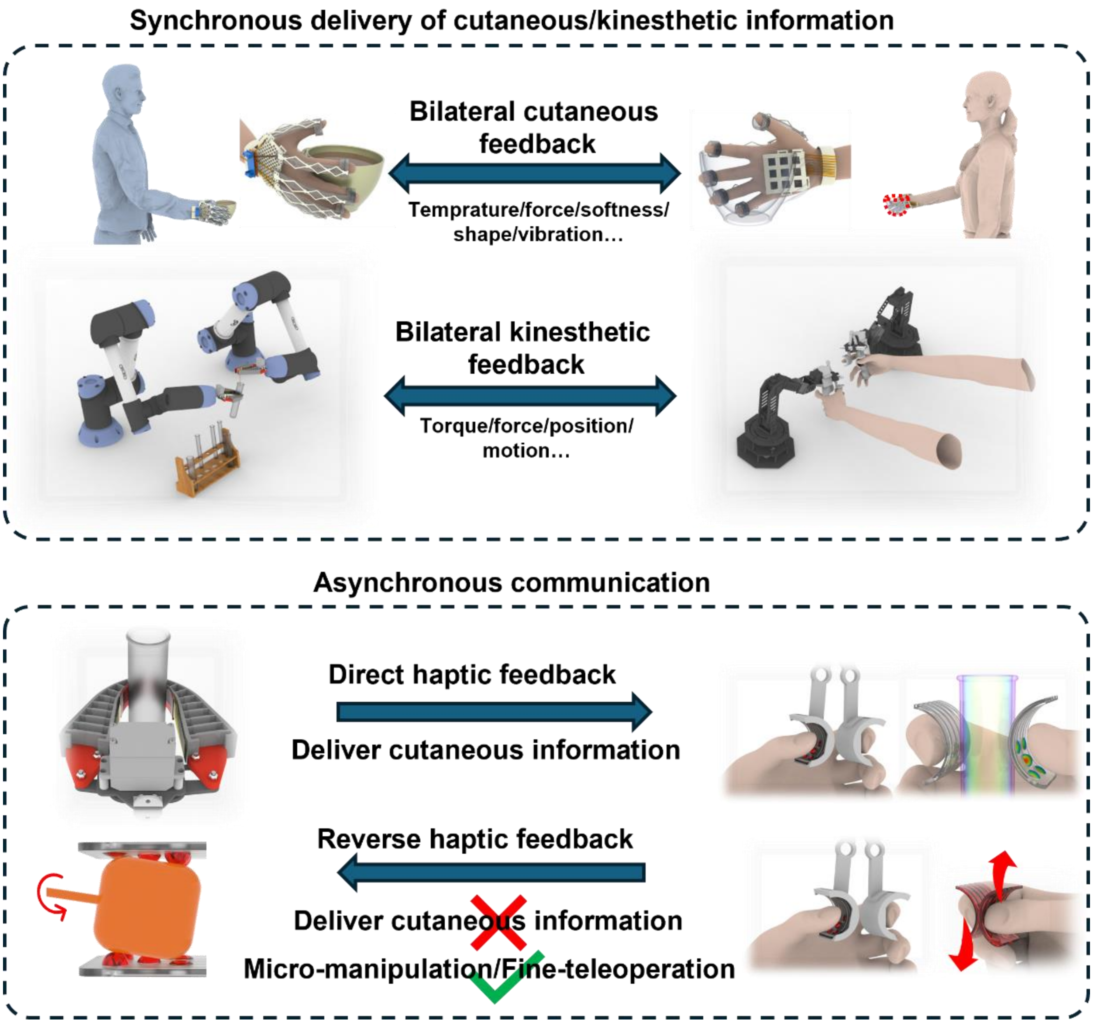

Fig. R20. Comparisons of the concepts between bilateral feedback and direct/reverse haptic feedback.

For "in-situ sensing" issue, we believe that it can be a sub-category of "collocation" of sensors and actuators. However, it may not be suitable for simply using "collocation" to replace in-situ sensing. Collocation of sensors and actuators can simply be done by combining or bonding them together. In tactile sensor field, there are also many groups working on developing multi-modal sensors at the same pixel via stacking or other approaches. Similarly, same method is also used in the integration of sensors and actuators. But stiffness of substrate or even functional element itself may affect each other, in terms of sensitivity or actuation force. In-situ sensing utilizes actuation membrane itself as a substrate for sensor. Together with liquid metal, the whole sensing unit has less influence on the actuator. As a typical example shown below (doi: 10.1126/sciadv.adt4839), this work uses "collocation" of sensors and actuators to develop a haptic feedback device with tactile sensing capabilities as well. However, as the photo microsensors are rigid commercial electronics, they may affect entire flexibility of the device and limit its application as manipulator in our case (vulnerable to gripping force and makes the surface uneven).

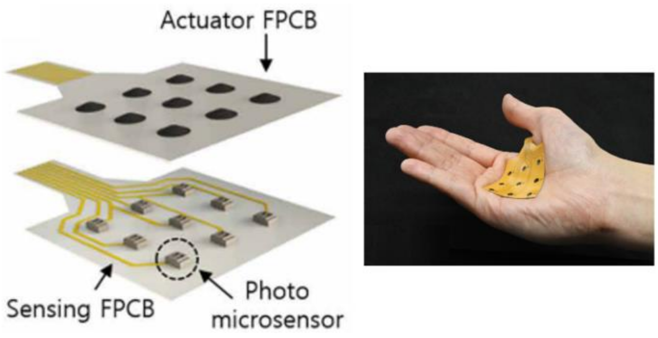

Fig. R21. Example of collocation of sensor and actuator.

- Does the work support the conclusions and claims, or is additional evidence needed?

"As an example of TACHIP, several key parameters are concluded from systematic analysis of the subjective test data and the corresponding sensing signals. Specifically, as shown in Fig. 5a(i), actuation pressure (Px, x is the index of actuator), differential pressure(\Delta Px-y, x and y are the indexes of actuators), inflation time (tx), gap time (\Delta tx-y), and frequency (fx), etc., are essential for static and dynamic feedback."

This reviewer wonders how these key parameters are systematically selected out of many potential quantities. It seems that the authors select these key parameters through their intuition. If not, show the procedure to select the key parameters.

We thank a lot for the valuable comment and apology for the unclear description. As mentioned before, most of the current haptic feedback research focus on the scientific contribution in mechanism and hardware, in order to generate different stimuli with varied pixel densities or intensities. However, fewer research is trying to investigate the actual performance of the perception received by users. The frequently used method is only subjective questionnaire, statistical data of survey, and confusion map (target perception vs. actual perceived feeling). As one of the foundations for generating those perceptions, quantitative evaluation about the parameters of haptic feedback pattern is rarely presented. Without the database collection of those key parameters as reference, each haptic feedback pattern will need cyclic procedures (programming, subjective test, reprogramming, subjective test, …) to reach a user accepted performance. We believe that it will become a major concern once haptic feedback technology is popularized in the future, due to the massive amount of diversified perception.

Therefore, we started our primary trial of investigating those parameters and the way of obtaining the related data in this manuscript. Here are the detailed descriptions

- Actuation pressure (Px, x is the index of actuator): the input pressure of actuating an actuator, and is directly related to displacement and hardness.
- Differential pressure (\Delta Px-y, x and y are the indexes of actuators): the differences of input pressures between the adjacent actuators. It affects identification of displacement variations between two actuators, in another word, the perception of shape/geometry/contour.

- Inflation time (tx): time duration of actuation of each actuator. It affects effective stimulation time.
- Gap time (\Delta tx-y): gap time between the deactivation of actuator x and the activation of actuator y. It affects the identification of temporal information between two adjacent actuators.
- Frequency (fx): actuation frequency of an actuator.

In fact, the purpose of tactile communication is to deliver spatiotemporal information of various stimulus. We adopt the concept of Exploratory Procedures (EP), which is proposed by Lederman and Klatzky in 1987. EP is purposive action pattern that perceivers execute to encode properties of surfaces and objects, and mainly consists of six procedures: (1) lateral motions for surface texture, (2) pressure for compliance or hardness, (3) static contact for apparent temperature, (4) unsupported holding for weight, (5) enclosure for volume and shape, (6) contour following for exact shape. This concept indicates the importance of spatiotemporal information in exploring objects or dynamic events. In terms of spatiotemporal information, it mainly consists of time and position/displacement/shape. In our case, time information is represented by inflation time (tx), gap time (\Delta tx-y), and actuation frequency (fx). Position/displacement/shape information is represented by actuation pressure (Px) and differential pressure (Px-y).

As can be seen in Figure 5, we provide 4 confusion maps indicating the results of subjective tests which actually evaluate the key parameters. For instance, frequency recognition test shows the perceivable variations of actuation frequency (fx). Hardness recognition test shows the perceivable variations of actuation pressure (Px), and inflation time (tx). Shape recognition test shows the perceivable variations of differential pressure (Px-y), Static and dynamic pattern test shows the perceivable variations of gap time (\Delta tx-y).

In terms of how to verify the importance of these parameters during the selection procedures, we have conducted the basic modifications of haptic feedback program, along with the followed subjective tests. As an instance we mentioned in manuscript, there is a relative high rate of false identification between pattern 4 and 9 in Figure 5b. Pattern 4 is a static haptic feedback which has three pixels activated in a diagonal direction. Pattern 9 is a dynamic haptic feedback pattern sequentially activated five pixels in a diagonal direction. Noticeably, pattern 4 is actually the same as one of three actuating stages of pattern 9. That is the possible reason for the false identification. More specifically, the possible key parameters affecting this result include low actuation pressure (Px) and fast gap time (\Delta tx-y). Low actuation pressure of U1 and U4 (P1 and P4), will cause low distinguishable displacement of U1 and U4. Fast gap time from U1 to U2/3/5 (\Delta $t_{(2,3,5)-1}$), as well as from U2/3/5 to U4 (\Delta $t_{4-(2,3,5)}$), will lead to less time of effective stimuli to skin. These two issues make Pattern 9 to be subjectively similar to Pattern 4. Thus, we controlled the variables to modify above two parameters for verifying the effectiveness of identification improvements. This is the typical procedure to select and verify the key parameters. In general, actuation frequency and input pressure are two tunable parameters which can modify the proposed key parameters. We will also appreciate it if there are other tunable quantities for us to investigate further.

It is indeed that there may be some missing aspects due to the limitations of our current knowledge. We will strive to improve this methodology and are willing to receive any kind of criticism and suggestions to help us. We have added extra descriptions in the main manuscript and the supplementary information.

"Differential pressures among actuators affect the perception regeneration of shape, geometry, etc."

This reviewer wonders how the authors can relate the differential pressures to perceptions. This review could not find any description supporting this claim. This question applies to the following sentence: "Feelings of texture and other dynamic events also depend on frequency of actuation".

We thank a lot for the valuable comment and apology for the unclear description regarding this contribution. As mentioned above, differential pressure (\Delta Px-y, x and y are the indexes of actuators) is the differences of input pressures between the adjacent actuators. It affects identification of displacement variations between two actuators. As an array based haptic feedback device, the displacements variations across the whole surface can modify the perception of shape/geometry/contour, as shown in Figure 4e(i) and 5c(i).

Similarly, actuation frequency of an actuator is important to replicate surface texture and dynamic event, such as sliding. According to exploratory procedure mentioned above, humans often use lateral motions for feeling the surface textures. The sliding event can also be sensed by skin. Sensory function, processed through skin within the human body's somesthesis, is enabled by four representative sensory mechanoreceptors. This system is divided into two FA and two SA mechanoreceptors, created by combining large and small receptive fields. In detail, a Merkel disk (MD) and a Ruffini cylinder (RC), used as SA receptors, continue to react as long as stimulation continues. By contrast, a Meissner corpuscle (MC) and a Pacinian corpuscle (PC), used as FA receptors, respond only at the beginning and/or ending moments of stimulation. Basically, when these mechanoreceptors are subjected to external stimuli, ions are transported through the ion channel to generate receptor potential. Receptor potential generated by the four receptors is converted into an action potential while they are transmitted through the axon; that potential has a specific frequency. Typically, perception of stimulus in human skin would need more than two mechanoreceptor signals. For example, humans feel skin motion (PC in FA) and texture perception (MD in SA) at the same time, and sometimes feel more of one than the other in different situations. Skin motion is measured by SA-II receptors, and large textures such as braille measured by SA-I receptors. Fine texture during rubbing can be monitored by FA-II receptors. Therefore, as FA receptor is in charge of fine texture perception with high frequency stimuli, and SA receptor is responsible for low frequency stimuli, the actuation frequency is then important for regenerating those feelings. We have added extra descriptions in the main manuscript.

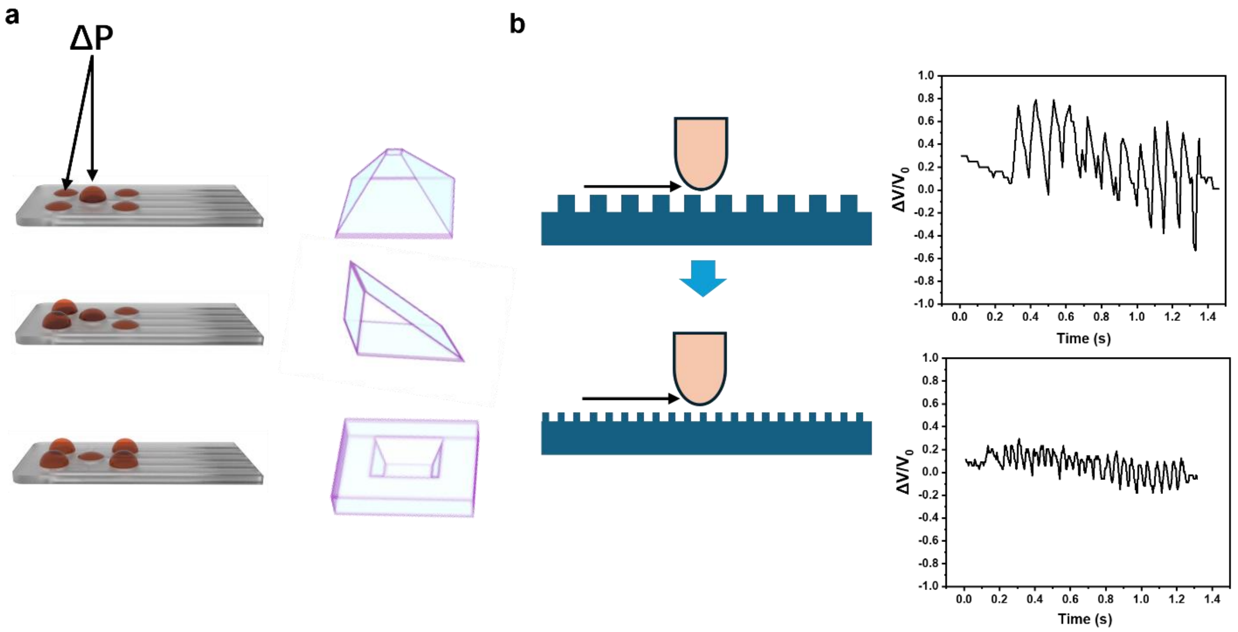

Fig. R22. Schematics of realizing shape and texture perceptions through **a.** differential pressures and **b.** actuation frequencies.

- Are there any flaws in the data analysis, interpretation and conclusions? - Do these prohibit publication or require revision?

We thank a lot for the valuable comment and apology for the flaws in the data analysis, interpretation and conclusions. All reviewers have thoroughly and carefully reviewed our manuscript and highlighted several concerns about data interpretation and standardization. We highly appreciate your professional and rigorous attitude shown in scientific research. We have tried our best to address all of the comments with detailed explanations, and have done considerable number of extra experiments. The major revisions have been done, and the corresponding parts in manuscript have been marked accordingly in red.

- Is the methodology sound? Does the work meet the expected standards in your field?

"providing target coordinates, the corresponding displacements of pneumatic actuators and their required input air pressure can be calculated."

Relationship between target coordinates and actuator displacements is a simple geometric equations, which can be avoided from the main text.

This reviewer wonders why "Newton downhill method" is applied to solve these geometric equations. Automatic differentiation could be applied to such simple equations.

We sincerely thank the reviewer for this insightful comment regarding the numerical method employed in our work. The reviewer's point is well-taken, and we appreciate the opportunity to clarify the rationale behind our choice of the Newton downhill method.

It is important to emphasize that the relationship between the target coordinates and the actuator displacements in our parallel manipulator system is not governed by simple, explicit geometric equations. As detailed in the kinematic analysis presented in the manuscript (and further elaborated in Supplementary Note 8), the system's kinematics result in a set of highly nonlinear and strongly coupled transcendental equations. Due to this inherent complexity, an analytical solution is intractable.

In this context, an iterative numerical solver is essential. We selected the Newton downhill method—a variant of the classical Newton's method enhanced with a global convergence strategy—for its robustness and computational efficiency in solving such nonlinear systems. While automatic differentiation (AD) is indeed a powerful technique for efficiently computing derivatives, its application in our case would still necessitate an iterative solver core due to the implicit nature of the equations. The Newton downhill method was chosen specifically for its balance between reliable convergence and handling the specific nonlinearities of our system, which helps avoid divergence issues that can occur with the standard Newton's method for certain initial guesses.

We realize that the original manuscript could have more clearly articulated complexity of the underlying kinematic model. Therefore, we have revised the relevant sections to explicitly state that the relationships are transcendental and require robust numerical methods for solution, thereby providing a clearer justification for our choice of the Newton downhill method.

- Is there enough detail provided in the methods for the work to be reproduced?

"Eleven objects, including sphere, cylinder, frustum, cube with four orientations, trapezoid with four orientations, were selected for collecting the training dataset."

Here only geometric shapes are focused on. This reviewer wonders what happens when natural shapes with much irregularity are applied.

We thank a lot for the valuable comment and careful inspection of this issue. The reviewer raises a very popular question regarding this kind of demonstration. Currently, grabbing object recognition is a very common experiment in tactile/visual sensing research to verify machine learning assisted multi-channel sensing function. We would like to address this comment from two aspects.

First of all, let us assume that there will be no more change or modification of the current gripper and the amount of TACHIPs, as well as the distribution of TACHIPs. As the algorithm we used was supervised learning, there were fixed labels or categories pre-trained specifically for those presented

geometric shapes. Obviously, any other natural shapes will be recognized and categorized into one of those pre-trained labels/shapes, depending on how it is grabbed. The contact status of the grabbed natural shape object will most likely be labelled as one geometric shape which has similar contact status. We can also pre-train the new object to add new label in database, but the recognition accuracy cannot be guaranteed if we do not modify the gripper, due to the lack of comprehensive contour information caused by the surface coverage issue of gripper finger and sensor. Simply speaking, the current setup of robotic platform still has certain limitations in general applications. Based on exploratory procedure concept, we can also adopt multiple grabs with different positions to improve the coverage for better recognition accuracy. But it may come with other problems.

Secondly, we can increase the number of gripper's fingers or completely replace the gripper by other soft manipulator with better surface coverage. Thus, the scalability of TACHIPs can be utilized for distributing across the soft manipulator. In this case, the natural shapes with more irregularities can be sensed thoroughly by new structure, which eventually improves the recognition accuracy. It is worth noting that the data for pre-training is still needed for building the model.

Moreover, it will be more suitable of applying visual-tactile sensing fusion in the practical applications of dexterous manipulation. It allows collaborative collection of information to solve the issues raised by the drawbacks from both sensing techniques. In addition, to focus more on the main contributions, we have moved this experiment results into supplementary information, with extra discussion about the irregular object.

"Three hardness levels were tested by changing the air pressure induced resistive force of pneumatic chamber, including hard, medium, and soft, are labelled as pattern 1, 2, and 3, as shown in Fig. 5c(ii)."

This reviewer wonders how much these softness values are. Please show softness quantitatively (Young's moduli or Shore A hardness values).

We thank a lot for the valuable comment and suggestion. We have added the hardness test results accordingly.

Table R3. Stiffness/hardness of actuator under different situations.

	Shore A hardness
Soft	6 HA
Medium	10 HA
Hard	14 HA

Noticeably, we were not able to mimic the real hardness of the rigid objects, due to the nature of elastomeric materials. Thus, the main objective of the air pressure-based hardness haptic feedback is to provide qualitative indications about the hardness differences among various objects. We have added the hardness values into the manuscript accordingly.

"Fusion of haptic and thermal feedback augmented teleoperation."

"In general, fusion of thermal and haptic feedback with the varied detail information across the area can give enhanced perception recreation during precise teleoperation."

Unfortunately, only one description on "enhanced perception recreation" can be found in this manuscript. This reviewer wonders how this can be generalized.

We thank a lot for the valuable comment and apology for the unclear description. Mechanical and temperature perceptions are two key tactile information which affect efficiency and safety during human machine interactions. Enhanced perception regeneration provided by fusion of mechanical and temperature feedback can offer comprehensive understanding of real-time on-site condition during operation and assist the corresponding adjustment. In the research field of cutaneous haptic feedback, most of the related work reported either mechanical or temperature stimulation. Fewer of them demonstrated the integration of mechanical and temperature feedback. However, those integrated devices usually encounter several issues. For instance, discrete distribution/location of mechanical actuator and temperature stimulator causes spatial inconsistency of feedback information. Simple combination of commercial actuator and heater at the same position (collocation) causes bulky and rigid structure.

In this manuscript, we utilized the resistive heating effect of liquid metal within the actuation membrane (which is also an in-situ sensor) to provide thermal feedback accompanied with each pneumatic actuator. It is then feasible to offer simultaneous thermal and mechanical feedback with high spatial consistency. As an example, when contacting with hot object, the temperature distribution of gripper/robotic hand usually shows good correlation with the contact pressure distribution. Therefore, TACHIPs with fusion of thermal and mechanical haptic feedback can enhance the perception of the manipulated object, by providing not only the information of shape/geometry/hardness/dynamic motion, but also the temperature variations of the contacted positions. Thus, users are experiencing enhancement of tactile perception via multi-modal stimuli. This is the main reason of saying "enhanced perception regeneration/recreation". We have added additional description at the beginning of this section.

"According to this information, the modification of the program can be done for all haptic feedback patterns to ensure better performance."

This reviewer wonders how this modification can be achieved.

We thank a lot for the valuable comment and apology for the unclear description. First of all, except sensing and feedback between follower and leader for real world interaction, there are also applications

of generating haptic feedback pattern for virtual objects or events. Hence, modification means the code reprogramming (e.g., delay, PWM parameters) of specific feedback pattern actuation. For a new haptic feedback pattern, such as dynamic sliding, the initial program is activated, and the in-situ sensing data of each actuator is recorded. According to database, if there are some key parameters showing high possibility of confusion (e.g., actuation pressure lower than perceivable threshold, gap time between two adjacent actuators is too short to be distinguished, etc.), the necessary modification of code (e.g., increasing of delay) can be conducted prior to the subjective test. After all of those potential issues are solved, the subjective test is then performed as double-check, and usually provides better accuracy for the first round.

As mentioned in previous comment, the fundamental issue about the performance of the haptic feedback devices and the corresponding feedback programs is the actual stimuli perceived by users. However, skin perception is a quite subjective aspect which may vary with different ages, genders, or even skin conditions. In the meantime, for different actuation mechanisms, e.g., pneumatic/fluidic pump, dielectric elastomer, electroosmotic pump, electrostatic pump, etc., there are various gaps between the coding of actuation program and the actual haptic stimulation perceived by user. As a traditional approach, each haptic feedback pattern will need cyclic procedures (programming, subjective test, modification by reprogramming, second subjective test, modification by reprogramming, third subjective test…) to reach a user accepted performance. We believe that it will become a major concern once haptic feedback technology is popularized in the future, due to the massive number of diversified perceptions. According to our experiment in Figure 5, the results suggested that cyclic procedures of "modification-subjective test" in traditional approach are probably caused by the missing of reliable quantitative sensing data of actuation. Thus, each modification in traditional approach is basically conducted by intuition which usually led to excessive or insufficient tuning, and requiring time consuming subjective evaluation.

With in-situ sensing capability of haptic actuator, the established sensing database with key parameters can be analyzed and linked to the coding of actuation program. Although subjective tests are still required to build the database at early stage, the involvement of cyclic subjective tests for each pattern can be significantly reduced by utilizing real-time sensing data. As the database keeps accumulating, less or even no subjective tests are needed by cross-checking the similarities between recorded and new haptic feedback patterns.

"As shown in Fig. 8c(i), the key units for identifying the directions of the finger twisting motions are highlighted as black dots."

This reviewer wonders what "key units" implies. Are "key units" same as "key parameters"?

We thank a lot for the comment and apology for the unclear description. Key units mean the physical items, or we may call them "key elements/key pixels". As can be seen in Figure 2, we labelled 5 pixels

of TACHIP as U1, U2, U3, U4, and U5, which stands for Unit 1, Unit 2… Therefore, the description highlighted by reviewer means that, there are pre-defined key pixels corresponding to the different twisting directions. A specific twisting direction can only be identified when the pressed key pixels match with the pre-defined condition. We have added extra descriptions in the main manuscript.

Dear Reviewers:

Your comments are very valuable, insightful, and helpful for revising and improving our manuscript. We highly appreciate these rigorous inspections which help us to fulfill the standard in scientific research. We have studied all of the comments carefully and made considerable number of revisions which we hope can address your concerns and meet with your approval. We also would like to provide further explanation or experimental data if additional concerns are raised. Briefly speaking, we have done extra experiments to collect new data, and added the additional discussion into the main manuscript in response to reviewers' comments. We have revised Fig. 3, and Fig. 6, and added supplementary Fig. 6, 11, 12, 17, and 21, and modified supplementary movie 3. We have made significant modification to avoid the overclaim and misleading presentation. All of the revisions in the main manuscript and the supplementary information are marked in red, and the point-by-point response to the reviewers' comments are provided as following:

REVIEWER COMMENTS

Reviewer #1 (Remarks to the Author):

Thank you for your detailed response.

Your responses were sufficient to clear up any questions I had.

We thank a lot for the valuable time and support from the reviewer for helping us to improve the quality of the manuscript.

Reviewer #2 (Remarks to the Author):

The revised version of the manuscript shows substantial improvements in clarity, experimental detail, and system integration. However, several technical and validation aspects remain incomplete and should be strengthened before the paper can meet the standards of a Nature-tier publication. The following points summarize the remaining issues to be addressed:

1.The description of the liquid-metal sensor's voltage divider lacks quantitative justification for linearity and thermal stability. The authors should clarify how resistor matching, calibration, or compensation mitigates nonlinearity and temperature drift during operation.

We thank a lot for the valuable comments and suggestions. Nonlinearity and temperature drift are the common issues experienced by those piezoresistive sensors. We can mitigate these issue through two approaches, hardware and software. For hardware part, the temperature sensor can be applied to

compensate the drift. For software part, we can apply regional linear functions to reduce the accuracy issue caused by nonlinearity.

Specifically, as can be seen in the characterization data of liquid metal sensor in Fig. 2c and supplementary Fig. 3, the curves are indeed showing some nonlinearities throughout the whole sensing range. However, we also observed that each curve can be separated into two regions with relative good linearities, including the region below 20 kPa, and the region above 20 kPa. This feature can be found in all of the characterization curves, even for the data about the temperature drift. Hence, we actually applied two linear fitting functions for processing the signals and converting them into pressure data.

The resistor matching of voltage divider was done by measuring the resistance of the as-fabricated liquid metal sensors. It is true that the temperature variations affect the baseline resistance of the sensor, and we also provided the temperature drift data in the previous response. The way of mitigating the temperature drift is to utilize the pectin temperature sensor as a reference sensor which is also immune from the mechanical stimuli, as can be seen in Fig.2 as well. This temperature sensor shows relatively good linearity in the temperature range which affects the liquid metal's resistance. Therefore, we calibrated the liquid metal sensor under varied temperatures and obtained the linear functions accordingly. Then, the signal processing program were applied with two conditional statements: (1) identifying the temperature range firstly; (2) followed by identifying the data of $\Delta V/V0$ (pressure level). The corresponding linear function with specific coefficient can then be chosen.

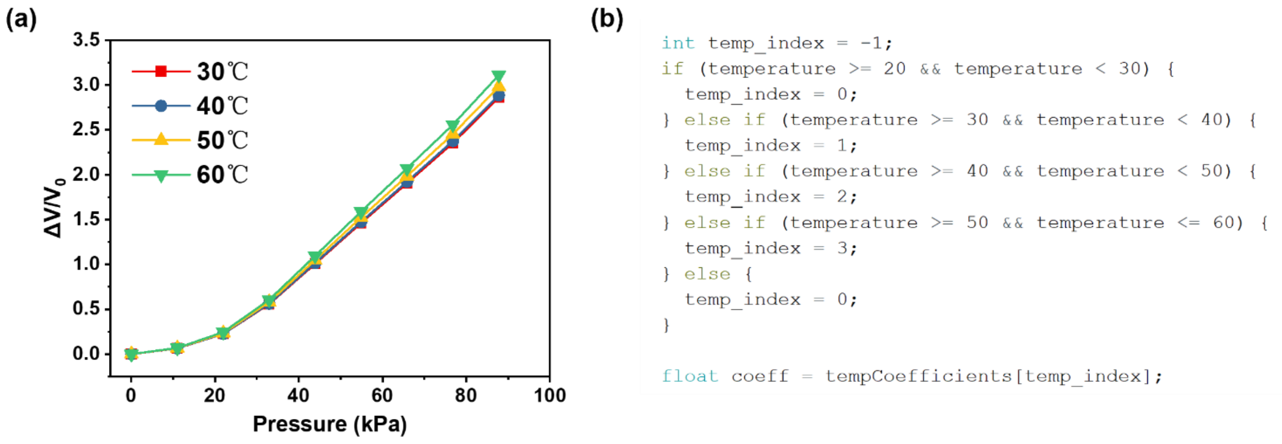

Fig. R1. Temperature effect of liquid metal-based pressure sensor under varied loadings and the implemented program.

2.The pneumatic actuator characterization should include quantitative error margins and hysteresis

curves. Additional calibration data are needed to validate the claimed dynamic response and ensure reproducible pressure–force behavior.

We thank a lot for the valuable comments and suggestions. We have repeated the corresponding characterizations for adding quantitative error margins. The hysteresis tests have been conducted as well. As can be seen in the figure, the pneumatic actuator show relative good reproducibility under specific input pressures. It also can be proved by the in-situ force sensing data at 16kPa input pressure. The sensing signal shows good consistency during the cyclic actuation.

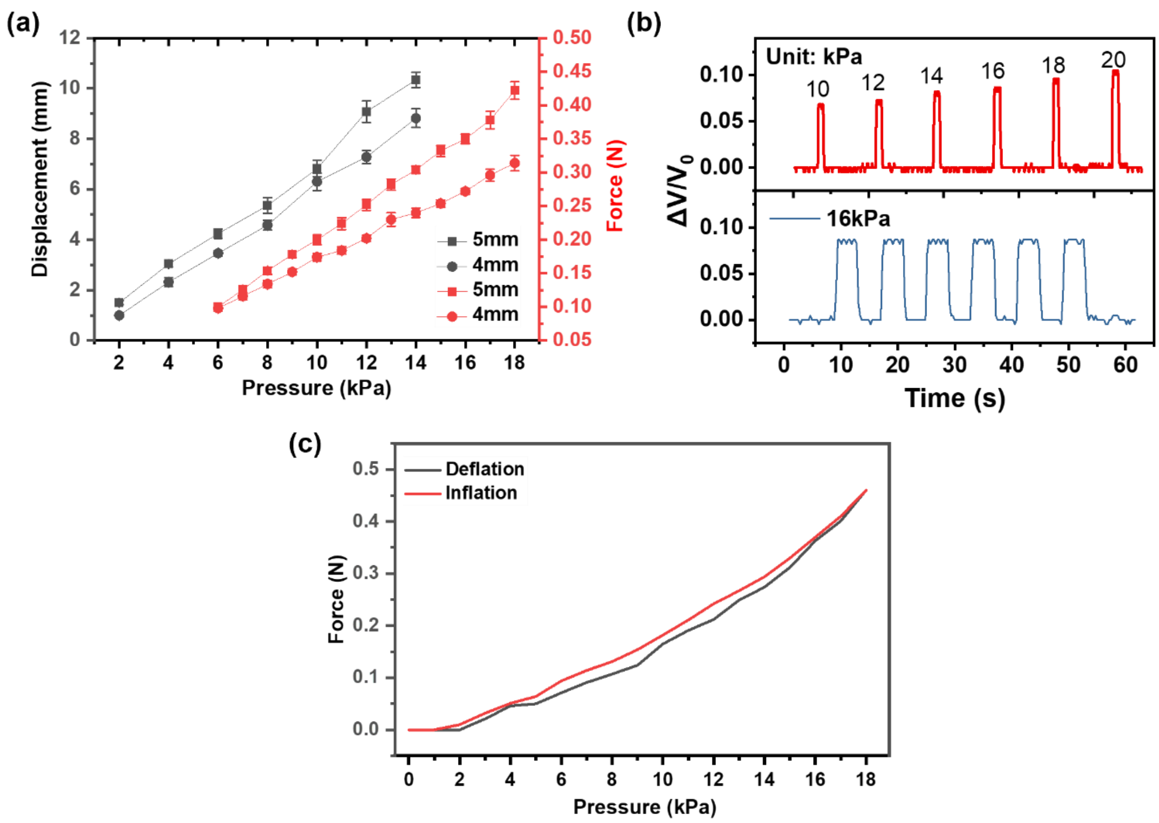

Fig. R3. **a**. Characterization data of pneumatic output forces with different design parameters. **b.** Sensitivity and stability of in-situ monitoring of pneumatic actuation via liquid metal pressure sensor. **c.** Hysteresis test of pneumatic actuation.

3.The paper reports tunable thermal feedback but lacks control-loop performance data. The authors should provide evidence of temperature stability, overshoot suppression, and long-term reliability under repeated cycles.

We thank a lot for the valuable comments and suggestions. The close-loop control is indeed a very important aspect of achieving tunable thermal feedback with high stability. In this manuscript, we have adopted PID control with the aid of temperature sensor. As can be seen in the figure below, the data comparison of the thermal feedback temperature profiles between PID control and normal open-loop control indicates that the PID control provides better overshoot suppression at the same heating rate and the target temperature. It also can be seen that the temperature stability after reaching the target value is good, without noticeable fluctuations or drifts.

Additionally, the long-term reliability of cyclic thermal feedback has been conducted as well. The temperature sensing signals of cyclic thermal feedback show a good reliability. It is worth noting that, the thermal feedback unit was cycled without the complete cooling process, due to the time consumption.

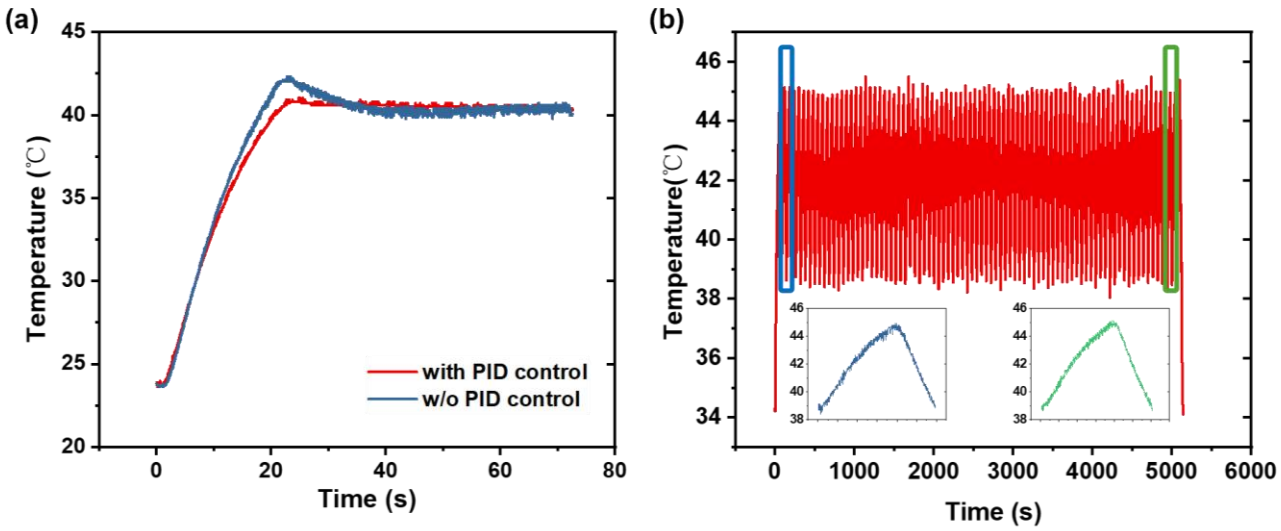

Fig. R3. **a.** Comparison of thermal feedback with and without PID control. **b.** Long-term reliability test under the cyclic operation with the target temperature of 45°C.

4.Since multiple sensors and actuators are integrated, the manuscript should quantify any potential electrical or mechanical crosstalk between pressure, temperature, and motion channels to confirm system-level fidelity.

We thank a lot for the valuable comments and suggestions. Crosstalk is indeed a very important issue to multi-functional and array-based devices. We have done additional experiments to address this issue from three aspects.

(1) Electrical and mechanical stability under temperature variations: We selected U3 as the activated unit for thermal feedback. The electrical signals and the internal pressures of the other four units, U1, U2, U4, and U5, were recorded to evaluate stability. Notably, all of the units were pre-inflated

with 10 kPa input air pressure for increasing the sensitivity to the possible crosstalk. As can be seen in the figure below, both the electrical signals and the internal pressures remained the same before and after heating.

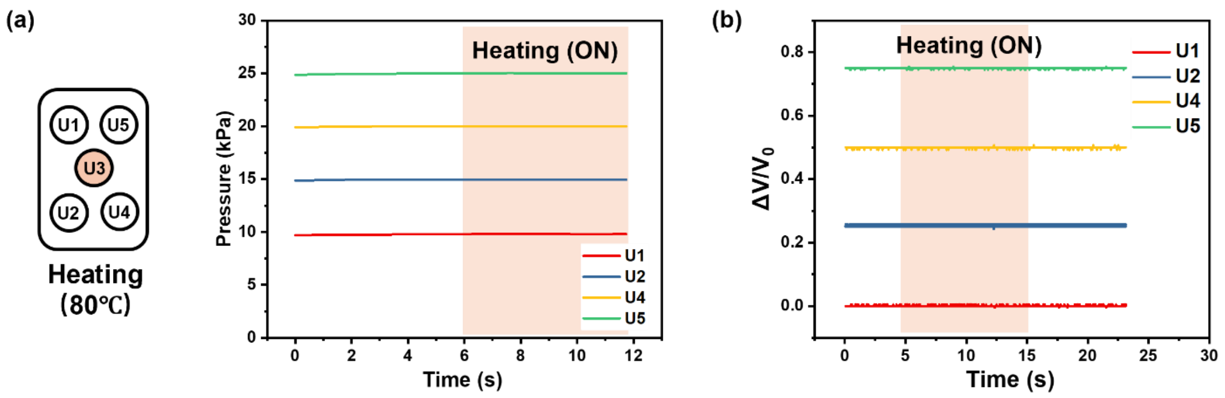

Fig. R4. **a**. Mechanical and **b.** electrical stability under temperature variations

(2) Electrical and mechanical stability under pneumatic actuations: similarly, we selected U3 as the activated unit for pneumatic feedback. As can be seen in the figure below, both the electrical signals and the internal pressures remained the same before and after inflation.

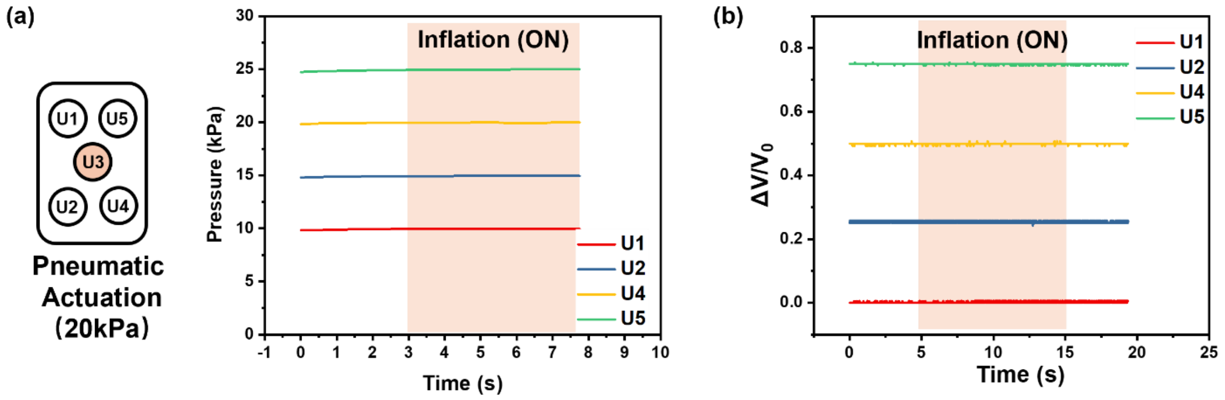

Fig. R5. **a.** Mechanical and **b.** electrical stability under pneumatic actuations.

(3) Electrical stability under pressing: We selected U2 as the activated unit for sensing the contact force. As can be seen in the figure, the liquid metal interconnector of U1 lies next to U2. The excessive contact force to U2 may cause the compression of the interconnector of U1, and lead to false detection (crosstalk) at U1. The experimental results also proved this issue. To mitigate this problem, we have designed small contact points on the pneumatic actuators. The test results with contact points indicate the significant reduction of the crosstalk. Moreover, it is clear that this type of crosstalk cannot be completely eliminated if the contact force is much larger than the designed

purpose. Thus, we may also use other materials, such as silver paste, as the interconnector, if there is higher requirement to the crosstalk issue.

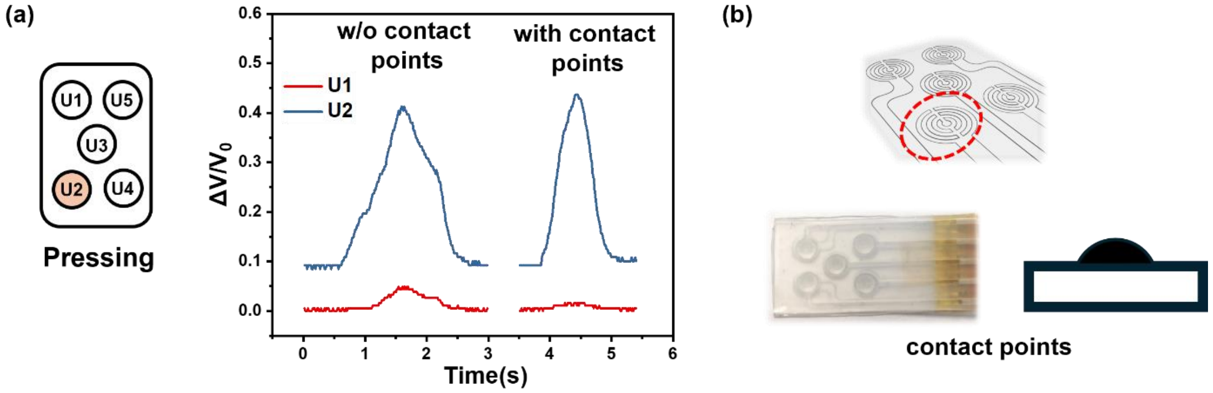

Fig. R6. **a.** Electrical stability under pressing. **b.** Schematics of pressing induced cross talk and the modified structure for reducing the crosstalk.

5.Comparative benchmarking is essential. The authors should position TACHIP's performance (force range, frequency bandwidth, sensing resolution) against state-of-the-art haptic platforms or recent multifunctional tactile devices.

We thank a lot for the comments and suggestions. We have provided both the comparison table (supplementary table 1) and the visualized positioning of haptic feedback and sensing performances of TACHIP against other haptic platforms in our previous revision (Fig. 8b). The output forces, frequency bandwidth, and sensitivities of force and temperature are compared. Most of those works were selected from the recently published papers in Science and Nature-tier journals. However, most of haptic platforms do not offer the same amount of functions, and there are only three platforms available for the comparison of force sensing resolution, one platform for temperature sensing resolution. As a double check, the corresponding figures are provided below. Please let us know if this information still needs to be improved.

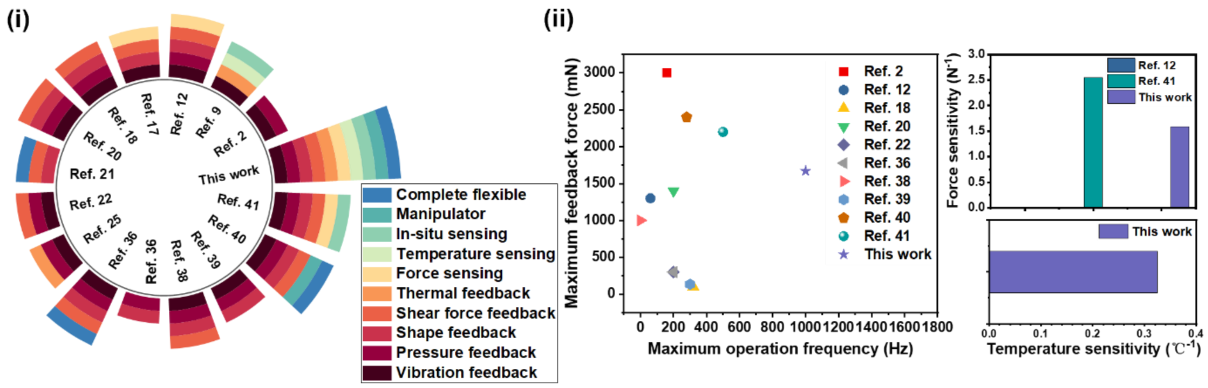

Fig. R7. a. Comparisons of (i) the functionalities and (ii) the general performances with the reported works.

6.While flexibility and modularity are highlighted, the practical aspects of large-scale fabrication, long-term reliability, and material degradation are not experimentally validated. A brief discussion or early test data should be added.

We thank a lot for the valuable comments and suggestions. We would like to give a brief discussion from two aspects.

First, in terms of salability, the main purpose of our claim is to indicate the applications of multiple TACHIP modules to achieve a certain level of scalability and distributive deployment. However, it still remains challenging to make a real large-area TACHIP with massive functional units. There are two key issues need to be solved, including the routing of pneumatic channels, and the fabrication of multi-pixel liquid metal channels with small feature sizes. Moreover, we have applied five TACHIPs to make a haptic glove to validate the scalability. Owing to the issue from the pneumatic pump, the increasing of TACHIPs will also cause the increasing of the pump module. Although the piezo pump used in our system is quite small, the massive deployment of TACHIPs may still lead to bulky size of the entire system.

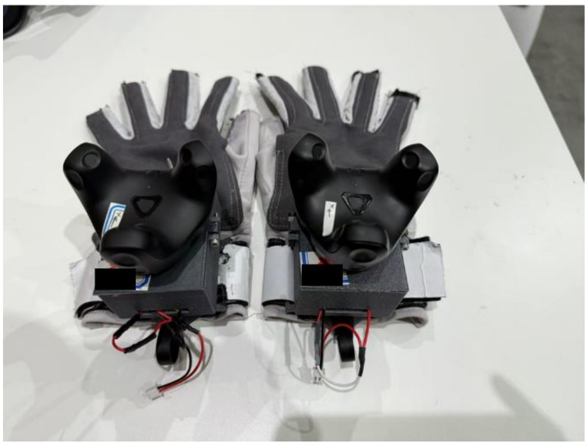

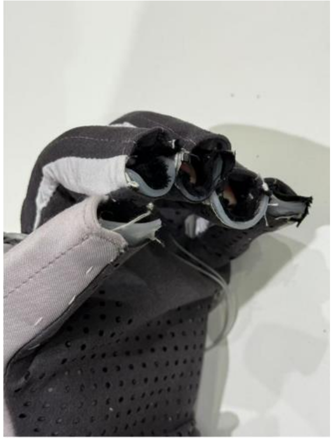

Fig. R8. Data glove developed by applying multiple TACHIPs.

Secondly, for long-term reliability and material degradation, we have selected the same TACHIP fabricated 14 months ago for conducting several reliability tests, which also have been shown in the previous revision (can be seen in supplementary figure). For the evaluation of mechanical stability, the cyclic folding and pneumatic actuation have been performed. For cyclic folding, the TACHIP has been folded under 15 N applied force for 2000 cycles. Next, the pneumatic actuation has been cycled for two months under a fixed input parameters (2 seconds per cycle, 50% input power), with the recorded internal air pressure and the real photos for observing the displacements. No obvious degradation is observed, which indicates relatively good mechanical stability. Due to the data storage capacity, to further validate its long-term durability, we have selectively recorded ~30 hours of accelerated test data (over 110000 cycles) as shown in figure below (1 second per cycle, 50% input power). For electrical stability, the sensing outputs of in-situ sensor for monitoring the pneumatic actuation shows no obvious drift of signal as well.

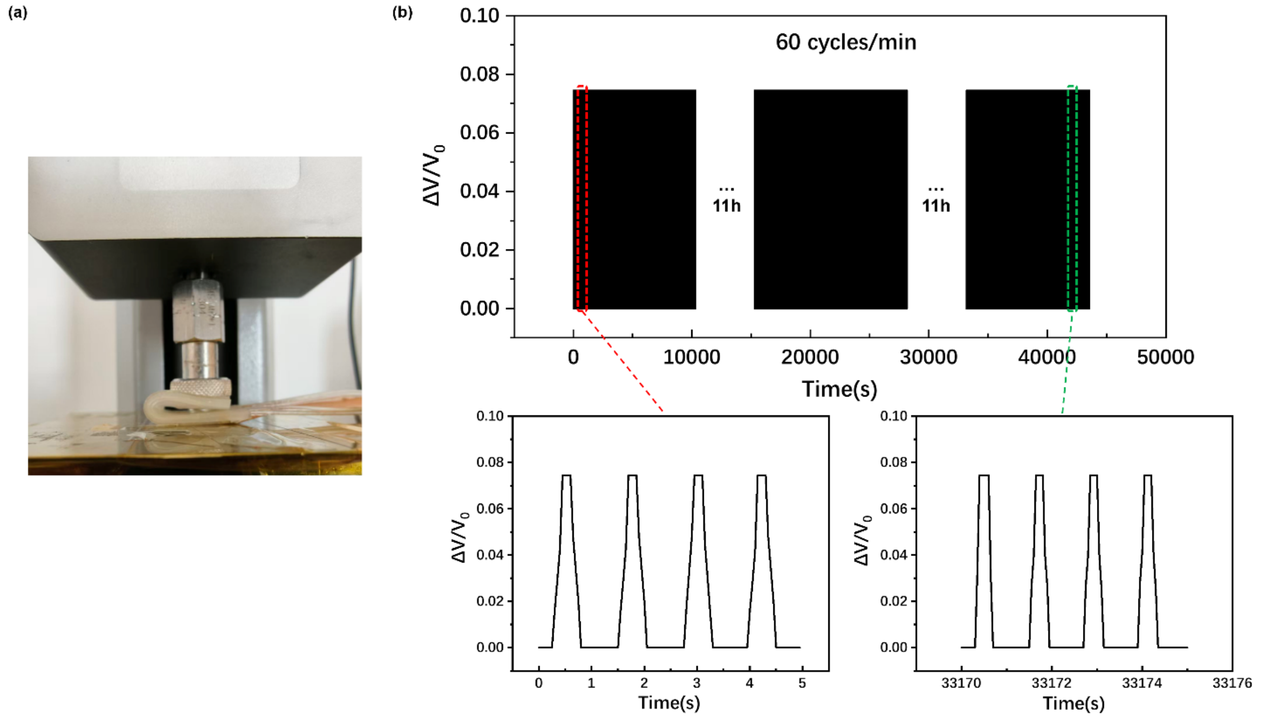

Fig. R9. **a.** Cyclic folding test under 15N applied force. **b.** Cyclic test results of pneumatic actuation and in-situ sensing within 30 hours (60 cycles/min, 50% of input power of pump).

On the other hand, it should be noted that the excessive input air pressure may cause significant material degradation in a short period of time, and affect the actuation performances immediately, or lead to the leakage of the air chamber. Hence, we have set the threshold of the pneumatic actuation with the help of in-situ sensor to prevent this situation. Meanwhile, the excessive external forces (above 30 N) applied to the TACHIP may also cause the leakage of the liquid metal and affect the sensing

performance, but the pneumatic actuator will not be damaged. In addition, as a quick plug-in module, TACHIP can serve as a fast replaceable and dispensable component which saves the maintenance time.

7.The dataset, signal-processing pipeline, and raw calibration curves need to be made publicly accessible or provided in Supplementary Information for verification.

We thank a lot for the suggestions. The main dataset, signal processing code, and raw data files have been uploaded into Springer Nature's figshare repository, including the MATLAB kinematic model, the dataset used for object recognition. The schematics of the print circuit board for the TACHIP system have been provided as well, for open source purpose. The system will generate DOI link later in data availability section.

Reviewer #3 (Remarks to the Author):

While the authors have addressed several presentation issues and added new data, a number of core concerns remain unresolved. In particular, the force fidelity, lifetime extrapolation, and micro-manipulation accuracy are still not supported by direct measurements. These issues do not necessarily preclude publication, but they require either additional validation or explicit narrowing of the claims to match the demonstrated capabilities.

1.I am not satisfied with the response to my first comment. While safety considerations may indeed necessitate force scaling (i.e., not rendering a 1:1 force magnitude), they do not support the statement that accurate quantitative force projection is only feasible within a safe force range. Fidelity is determined by the device's dynamic range, saturation, bandwidth, and nonlinearities, and accuracy should be defined relative to an explicit scaling law if such scaling is employed. Moreover, the manuscript does not describe any implemented force projection strategy (e.g., the claimed 5:1–10:1 ratios), nor does it explain how such scaling was applied in the reported experiments.

We thank a lot for the further explanation and suggestions. We now understand the major concerns of the reviewer better, with more details. We also apologize that the specific scaling law and the implemented feedback projection strategy were not discussed clearly in previous response.

First of all, we fully agree with the statement of "Fidelity is determined by the device's dynamic range, saturation, bandwidth, and nonlinearities…". In fact, we actually share a common understanding regarding the fidelity issue. This is also the basic reason for us to study the modalities of haptic feedback patterns in Fig. 5. We would like to address this comment by explaining two issues mentioned.

In terms of fidelity, the performances of TACHIP are shown in the characterization data. The pneumatic

actuator with piezo pump can provide a frequency bandwidth range from 0 to 1000 Hz, with perceivable displacement. As the perceivable frequency range of human skin is about 0 to 800 Hz, the operation frequency range shows a good match within skin's sensing range. For the output force, the maximum force generated by the pneumatic actuator is about 1.5 N, without non-linear behavior or irreversible damage. However, to avoid the influence on the control of handler and ensure the comfortability, the operation range of output forces is limited within 0 to 0.5 N. By considering the threshold of perceivable/effective mechanical stimuli during the teleoperation, the actual operation range of output forces is set as 0.1 to 0.5 N. Hence, the dynamic range of the TACHIP in our teleoperation application can be defined as F_{max}/F_{min}=500mN/100mN. Based on the above information, it is true that the dynamic range of the as-fabricated TACHIP's actuator may not match with the sensing range of the applied force. The scaling law is then required to project the applied forces into the dynamic range of force generation. Consequently, the tactile information resolution of the regenerated stimuli will decrease due to such scaling, and eventually affect the fidelity, especially for the forces which exceed the saturation value. To mitigate the possible operation failure caused by the fidelity issue, it is necessary to evaluate the key operation force range (e.g., minimum force before slipping, maximum force before break, etc.). Two different approaches to project the forces can be adopted.

(1) For complete linear projection, we set 500 mN as the saturation value. The key operation force range can be regenerated linearly from 100 mN to 500 mN. The input power for regenerating forces will not be increased after 500mN, regardless of the increased applied force.
(2) For nonlinear projection, we set 450mN as the saturation value, the key operation force range can be regenerated linearly within 100mN and 450 mN. The excessive applied forces can then be regenerated by different projection coefficient accordingly (from 450 mN to 500 mN).

On the other hand, the skin perception resolution within the dynamic range of the actuator is also essential to be determined, and this is also our ongoing work related to Fig. 5. As a result, the user can have general understanding about the operation force levels within the key operation force range, even though the smaller or larger forces are not generated, or generated non-linearly.

For implemented force projection strategy, we would like to use the integrated demonstration shown in Fig. 7 as an example. Firstly, for a specific application with a series of operations and objects, we have conducted the primary trials of manipulations to gather the general information about the range of the applied forces. In this demonstration, the complete linear projection has been implemented. The manipulations of test tube and relevant tools usually require the gripping forces range from 0.5 to 3 N. According to the dynamic range of TACHIP's actuator, we have applied 5:1 ratio for projecting the applied forces into the re-generated forces. For this light duty task, we focused more on the regeneration and the resolution of relative smaller forces. Although the maximum force will experience saturation after the applied force of 2.5 N, it will not affect the stability of manipulation in this case, the user can stop increasing the gripping force once perceive the maximum regenerated force. After the ratio of scaling was modified in the sensing and feedback program (change the coefficient between

the sensing signals and the input power of piezo pump), the operator performed a few trials to establish the projection relationship between the applied and the re-generated forces in mind, prior to the actual operations.

We highly appreciate the valuable elaboration from the reviewer, and the opportunity for us to provide detailed explanation. We hope this response can answer the reviewer's questions properly. The corresponding parts related to the haptic feedback fidelity and the implemented force projection strategy have been added into the results and the method sections, respectively.

2.The force-gauge versus TACHIP comparison further suggests that the TACHIP signal drifts relative to the actual applied force, with at least two intervals in which the externally applied force is increased to re-align with the TACHIP output before being increased again. This behavior indicates a non-trivial mismatch between sensed and applied forces. Therefore, validation using a controlled external force sensor is essential, particularly for a top-tier journal such as Nature.

We thank a lot for the careful inspection and suggestions. As mentioned in our previous response, the deviations/drifts of TACHIP's signal relative to the actual applied force were mainly caused by the structure and the method of measurement. We provide schematics in the below figure for assisting the explanation. The liquid metal-based pressure sensor in TACHIP is located under the silicone pneumatic actuator, which will affect the transmission of the applied force, and lead to noticeable drift. We also have used a controlled external force sensor to validate this phenomenon. As shown in figure, the external force sensor placed under the pneumatic actuator also shows the drifts relative to the actual applied force. In addition, we have repeated the force-gauge versus TACHIP comparison by applying the force directly onto the pure liquid-metal sensor side (flipped the TACHIP). The comparison results show a relatively good consistency between force-gauge and TACHIP.

The reason of applying force at the pneumatic actuator side for comparison is to follow the real application scenario, in which the pneumatic actuator side are placed outside for micro-manipulation, as silicone-based actuator shows better deformation capability compared to PDMS for liquid metal sensor. In our cases, the overall performance of force projection is still acceptable for improving operation success rate via haptic rendering. On the other hand, we also can flip the TACHIP to make the liquid-metal sensor to be outside, if the high consistency between sensed and applied force is required.

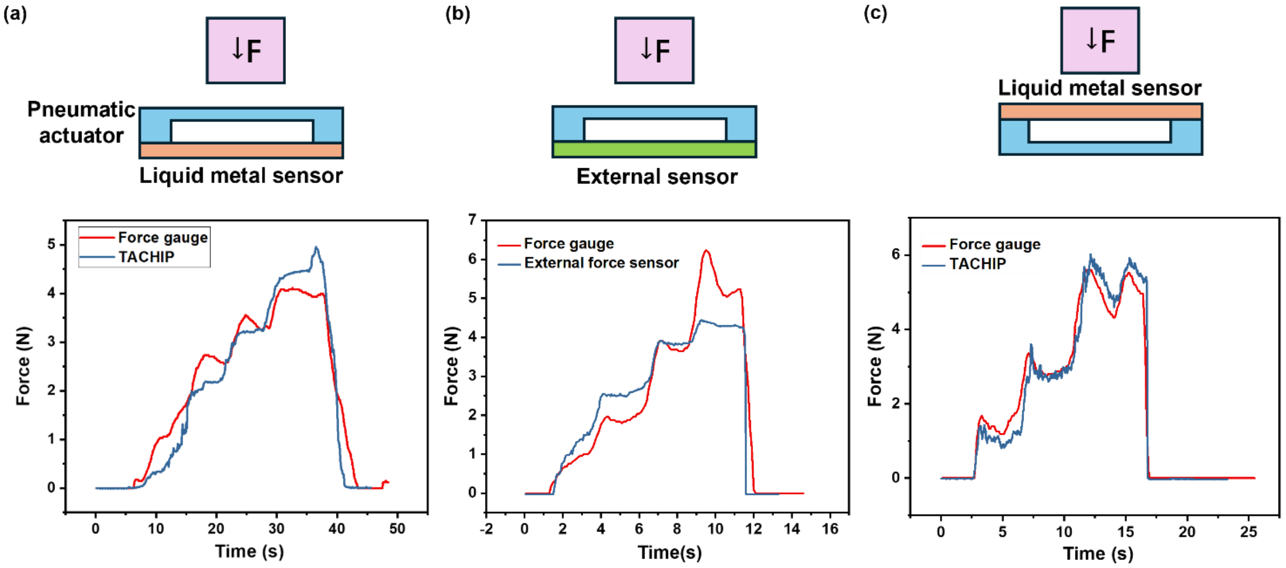

Fig. R10. Comparisons of force-gauge versus a. TACHIP with the pneumatic actuator side, b. external sensor under the pneumatic actuator, and c. TACHIP with the liquid metal sensor side.

3.The added cyclic test demonstrates short-term endurance (≈4×10^4 cycles) without obvious degradation, which is valuable. However, the conclusion that the actuators and sensors can "last for millions of cycles" is not supported by the presented data and appears to be an extrapolation without a defined degradation model, failure criterion, or accelerated testing. The manuscript should either quantify degradation metrics and justify this extrapolation or moderate the lifetime claim accordingly.

We thank a lot for the valuable comments and suggestions. We apologize for the improper description in the previous response. We are taking this comment very seriously and hope we can clarify the major concern. In fact, the given data of 4×10^4 cycles actuation was selectively recorded during the entire continuous reliability test. The cyclic test with the same TACHIP has continued for more than 6 weeks (≈2×10^4 cycles per day), and we are running the accelerated testing now as well. However, due to the data storage issue of long-term testing, we were unable to record the entire procedure. Here, to further validate its endurance, we have recorded the additional set of data (about 1×10^5 cycles) with the same TACHIP. It worth noting that this is the same TACHIP made 14 months ago, and was the TACHIP appeared in the supplementary movie.

We also agree that, it is not suitable for claiming the durability of millions of cycles without proof of the complete recorded data or the degradation model. To avoid the issue of overclaim, we would like to rephase the claim that the TACHIP can last at least a few hundred thousand of cycles, and there is no overclaimed durability in our manuscript. In addition, as a quick plug-in module, TACHIP can serve as a fast replaceable and dispensable component which saves the maintenance time. Meanwhile, we

will keep conducting the reliability test of that TACHIP to figure out the failure criterion.

Again, we sincerely appreciate the reviewer's careful and rigorous inspection which help us to further polish our manuscript.

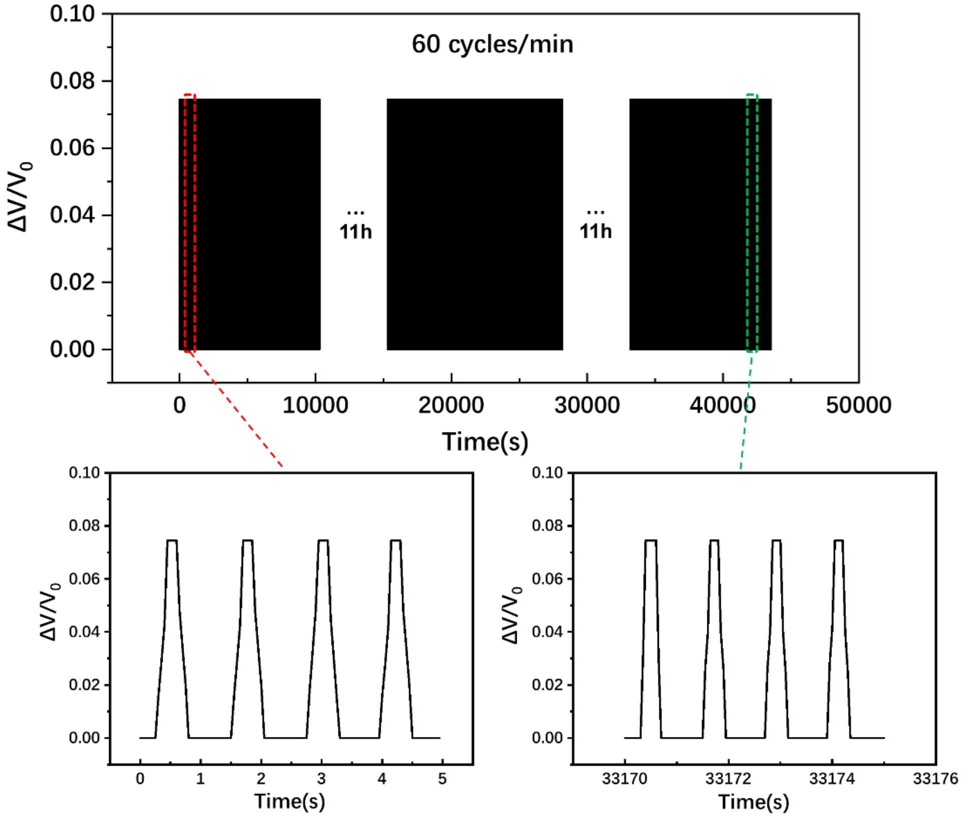

Fig. R11. Cyclic test results of pneumatic actuation and in-situ sensing within 30 hours (60 cycles/min, 50% of input power of pump).

4. Finally, the dashed trajectories in Fig. 6(d) appear to be reconstructed from actuator pressure inputs via the kinematic model rather than obtained from direct measurement of the end-effector motion. As no independent position sensing is described, these curves do not represent experimentally recorded paths. Consequently, this form of reporting cannot reveal the true physical accuracy of the manipulation and may give a misleading impression of tracking performance, since model-reconstructed trajectories inherently mask errors arising from compliance, slip, contact forces, and re-gripping.

We thank a lot for the valuable comments. We apologize for the possible misleading presentation of the real direct measured trajectories with the dashed lines. As mentioned in figure caption, all of the

dashed trajectories show the actual paths/direct measurement of the end-effector motion, and are not generated by the kinematic model. Currently, we do not have 3D space trajectory tracking equipment to record the path of the endpoint. Alternatively, we have placed the camera under the end-effector to record the video of the corresponding micro-manipulations. The dashed lines were manually drawn according to the video recorded real trajectories in previous response. As suggested by the reviewer, we have utilized visual tracking algorithm (OpenCV 2) to process the recorded micro-manipulation videos. The tip of the tool was attached with the red marker for enhancement of visual recognition. As can be seen in the figure, both the screenshots of recognized real-time trajectories and the recorded coordinates are provided by applying the output functions of OpenCV. However, it is also worth noting that the plots of the recorded coordinates show more fluctuations or deviations compared to the videos, due to the recognition accuracy of OpenCV. There are also some missing segments of the trajectories caused by the data acquisition rate.

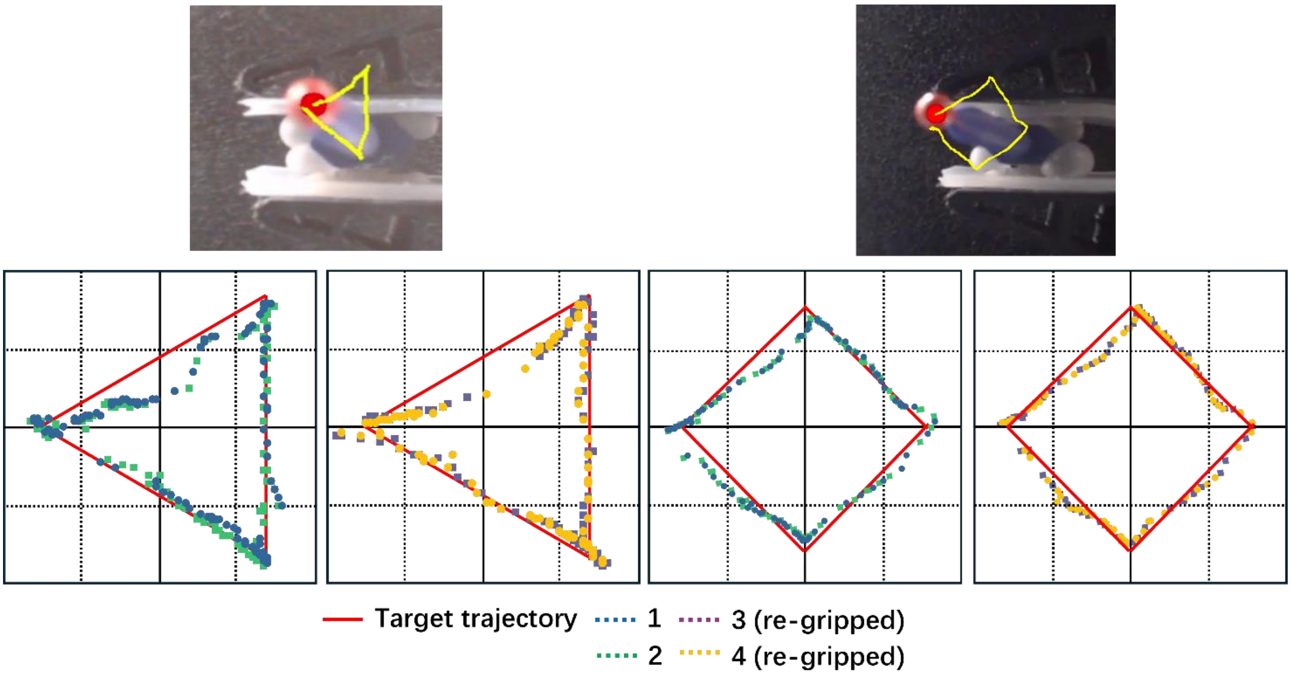

Fig. R12. Comparison of target trajectories (red line) and actual trajectories (dot line) for repeated manipulation and repeated manipulation after re-gripping.

According to the new data, the similar results mentioned in the previous response can be concluded, the real trajectories show several kinds of distortions compared to those of kinematic model (target trajectories), due to the reasons mentioned by us and the reviewer. For a single gripping, the repeated real trajectories show high consistency and repeatability (including those unwanted distortions), unless there are external forces applied to it. The action of re-gripping will indeed change the real trajectory slightly, and the following repeated cycles will also show high consistency with the new trajectory.

In addition, the kinematic model is actually generating the perfect target trajectories (also can be seen in supplementary figures), since it does not include the factors of compliance, slip, re-gripping, etc. As

further clarification, we have added the MATLAB animations of the kinematic model-based trajectories into the supplementary movie 3, prior to the real recorded clips. It can be seen that the end-effector in the kinematic model can draw a perfect triangle or square patterns on the spherical surface.

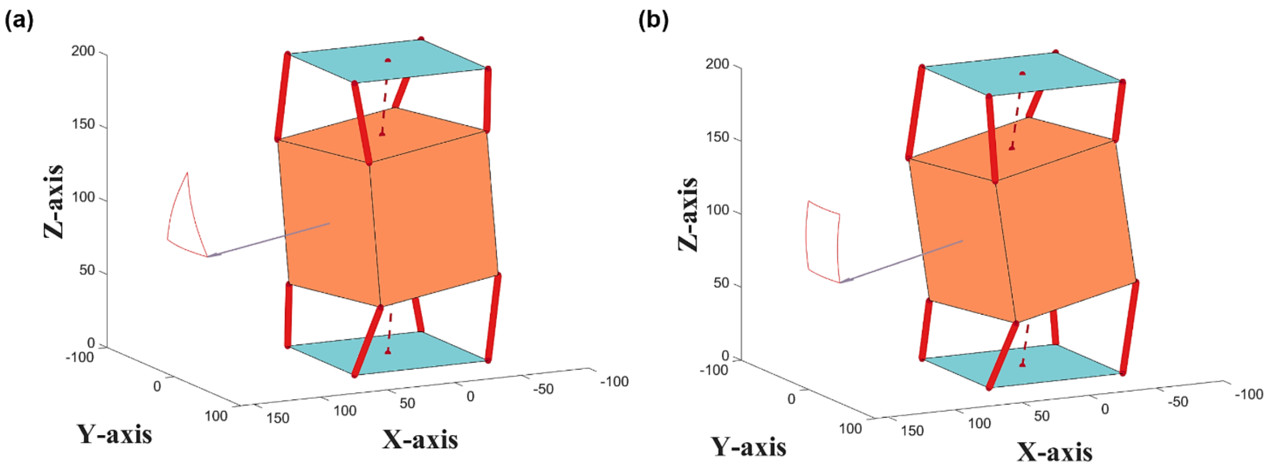

Fig. R13. Target trajectories drawn by MATLAB kinematic model.

Generally speaking, for high precision micro-manipulation, the TACHIP based manipulator has certain limitations from its materials and working principles. The main purpose of the proposed micro-manipulation function is to leverage the capabilities of haptic feedback devices for improving the motion DoFs of regular grippers, rather than making a parallel manipulator with high precision comparable to those specialized manipulators. The demonstrations of triangle and square trajectories simply serve as the proof of its motion DoFs when we need to adjust the object in a specific direction within its work space, not for the applications of drawing complicated patterns. The application scenarios focus on human-in-the-loop teleoperation, providing fine-tuning of objects without moving the main robotic arm. For this purpose, we can keep adjusting the actuators of TACHIP if the endpoint does not reach the target position at the beginning, due to the control accuracy issues. Meanwhile, we will also keep optimizing the working principle to improve the general performance of this reverse haptic feedback enabled micro-manipulation.

To avoid the possible overclaim or misunderstanding regarding our main purpose of micro-manipulation, we have modified the corresponding contents in the manuscript. The key idea remains that the reverse haptic feedback is to provide more degrees of freedom during the final stage of teleoperation, instead of utilizing the micro-manipulation to draw specific patterns. Together with kinematic model, both of two options can collaboratively support human-in-the-loop teleoperation.

Reviewer #4 (Remarks to the Author):

This revised manuscript represents a soft tactile chip (TACHIP) made by in-situ fabrication of sensors and actuators. The manuscript was revised to make clear the novel scientific contributions, haptic rendering enhanced by in-situ sensing and ulti-DOF micro-manipulation enabled through reverse haptic feedback. The authors conducted additional experiments demonstrating SA+FA perception, together with a thorough revision of texts and figures. The authors successfully mention that obtaining both subjective perception and sensing data with in-situ sensor reduces time consumption during dataset building. This reviewer raised several technical concerns as well as descriptive concerns; all of them were clarified. This reviewer is satisfied with the revision of this manuscript.

We thank a lot for the valuable time and support from the reviewer for helping us to improve the quality of the manuscript.
